# Supplementary figures and images for: Discovering molecular features of intrinsically disordered regions by using evolution for contrastive learning (part 1 of 3)
Source: PLoS Comput Biol. 2022 Jun 29;18(6):e1010238. doi: 10.1371/journal.pcbi.1010238 (PMC9275697; doi:10.1371/journal.pcbi.1010238)

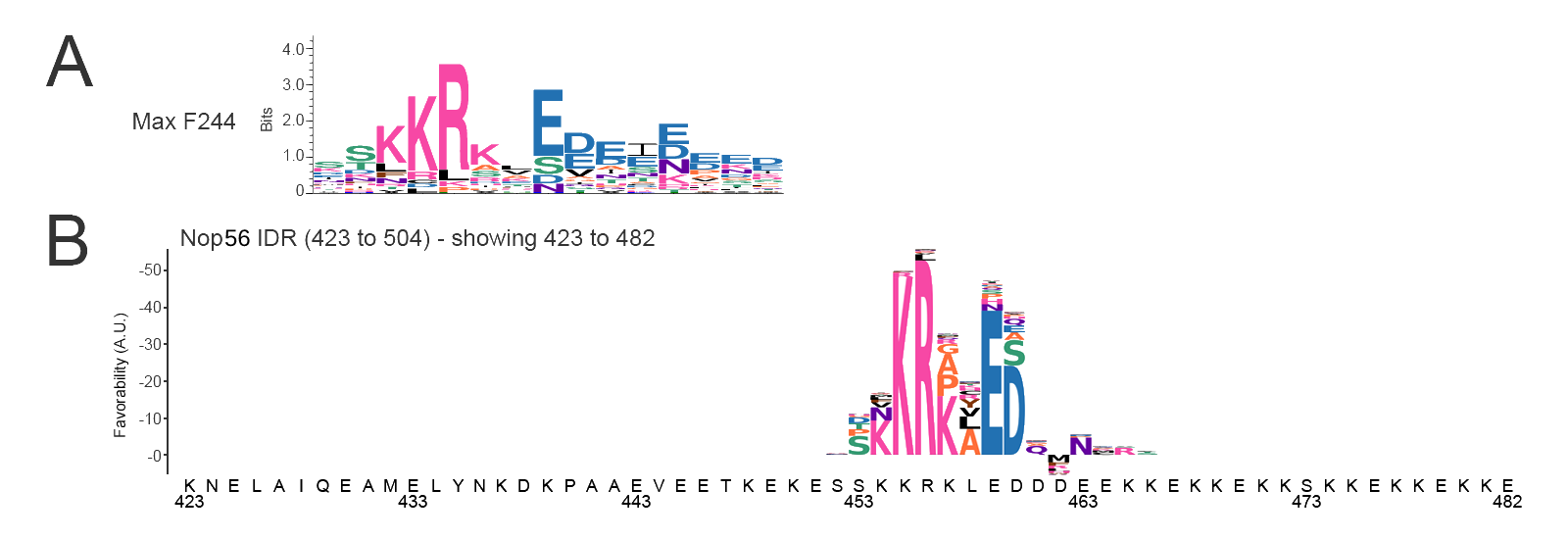

Supplement: S1 Fig — The C-terminus of yeast Nop56 contains alternating regions of positive and negative charge that activate yeast Max-pool Feature 244 A) The sequence logo for Max Feature 244 (Max F244) B) The mutation map (as in Fig 4) showing the importance of residues for activation of this feature in a section of the Nop56 C-terminal IDR. (PNG) [file pcbi.1010238.s001.png]

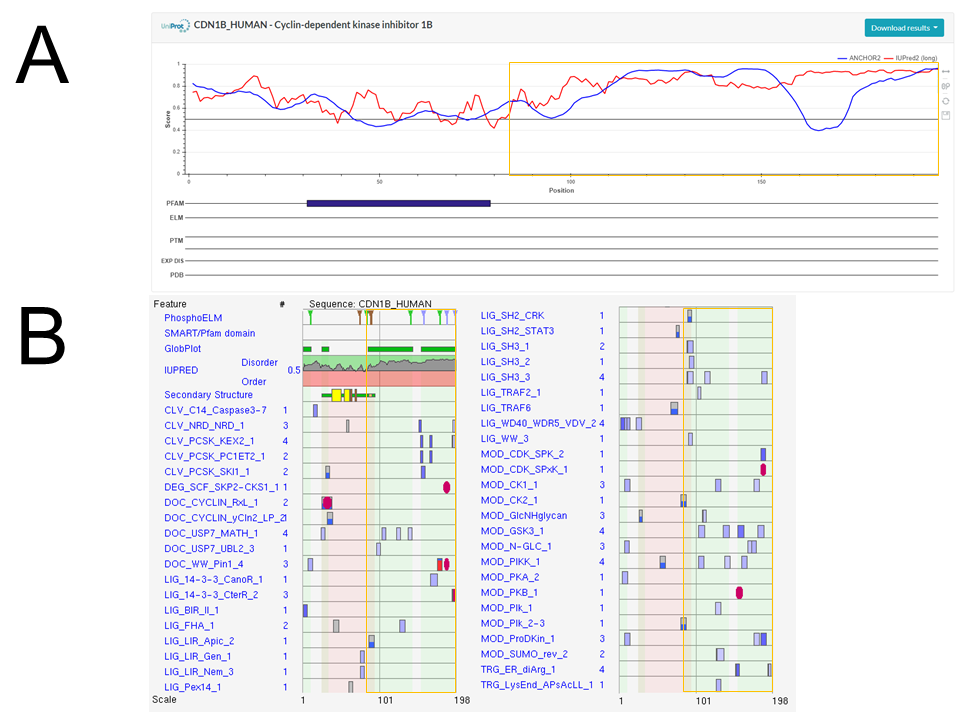

Supplement: S2 Fig — Predictions for p27 for ANCHOR2 (A) and ELM (B). For both predictions, we inputted the full protein, so we highlight the C-terminal IDR in gold. A) The blue line shows the ANCHOR2 score predicting disordered binding regions. B) The blue boxes show matches to short linear motifs within the sequence, as labeled on the left. The darker the blue, the more conserved the motif is across orthologues. The red circles indicate known instances annotated from literature. (PNG) [file pcbi.1010238.s002.png]

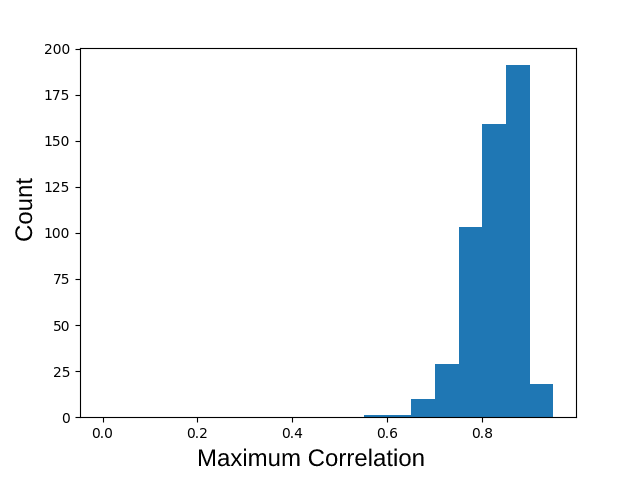

Supplement: S3 Fig — The distribution of the maximum correlation of features learned using the cropping heuristic used in the main text (128 residues at the start/end of sequence) and features learned in a model using an alternative cropping heuristic (256 in the center of sequence). Y-axis shows the number of features with that maximum correlation. Most features have a feature with at least correlation of 0.7. See Methods for more details. (PNG) [file pcbi.1010238.s003.png]

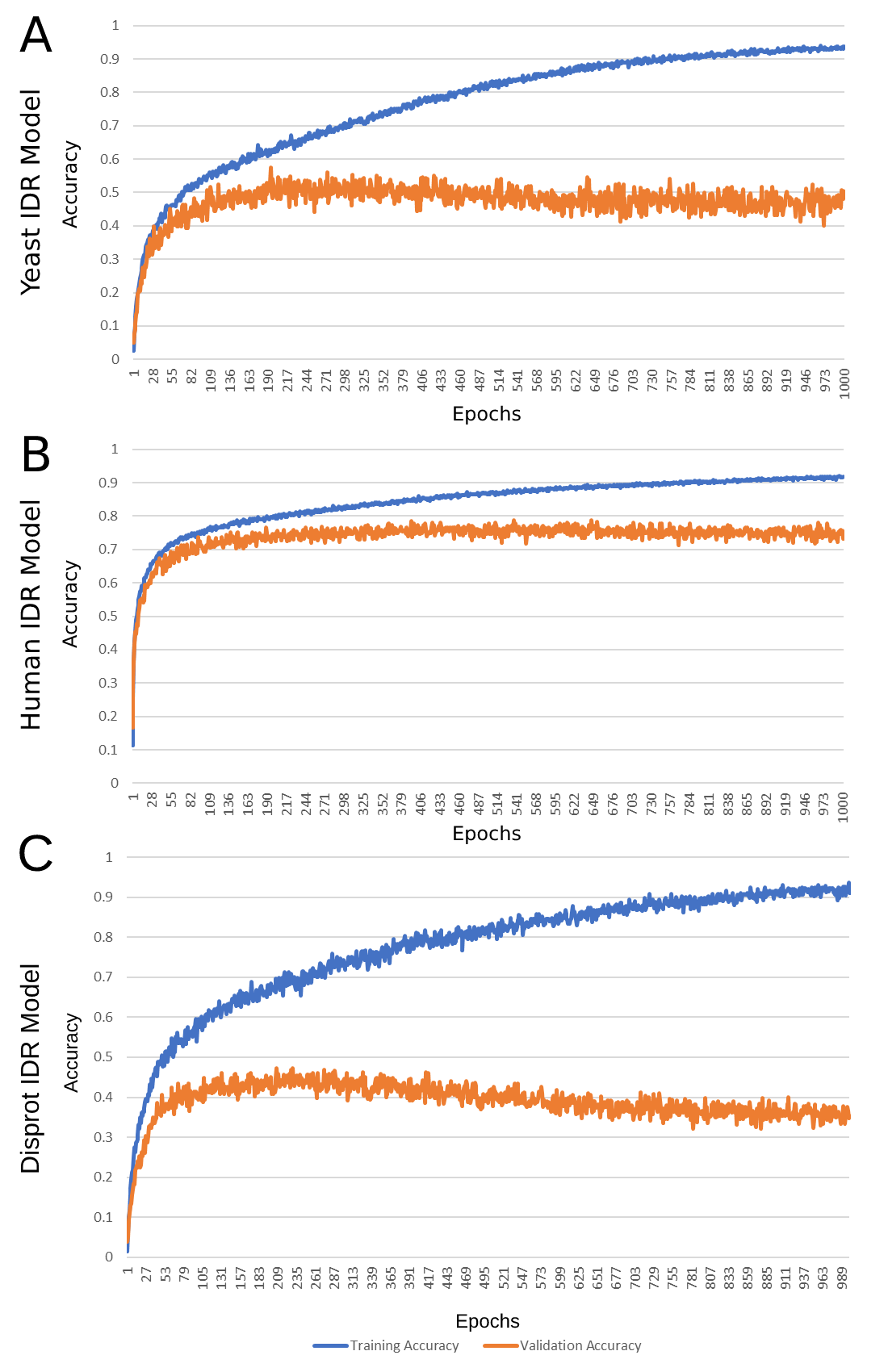

Supplement: S4 Fig — A) Model trained on yeast IDR homolog sets. B) Model trained on human homolog sets. See Methods for more details C) Model trained on experimentally characterized IDRs from DisProt. In this case, the validation set was 1000 randomly selected human homolog sets from the SPOT-Disorder human IDR predictions. (PNG) [file pcbi.1010238.s004.png]

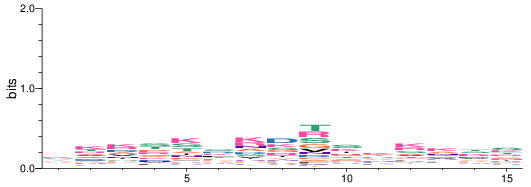

Supplement: S7 File — (ZIP) [file pcbi.1010238.s011.zip › disprot_html_table/logos/AVG_F0.png]

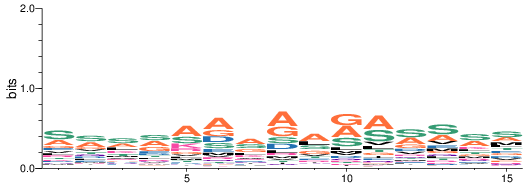

Supplement: S7 File — (ZIP) [file pcbi.1010238.s011.zip › disprot_html_table/logos/AVG_F1.png]

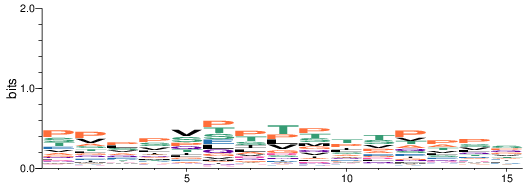

Supplement: S7 File — (ZIP) [file pcbi.1010238.s011.zip › disprot_html_table/logos/AVG_F10.png]

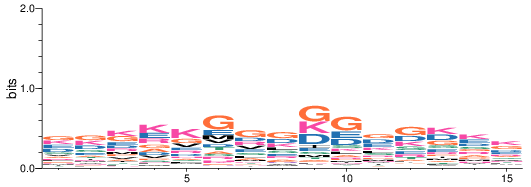

Supplement: S7 File — (ZIP) [file pcbi.1010238.s011.zip › disprot_html_table/logos/AVG_F100.png]

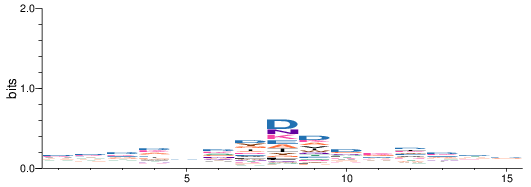

Supplement: S7 File — (ZIP) [file pcbi.1010238.s011.zip › disprot_html_table/logos/AVG_F101.png]

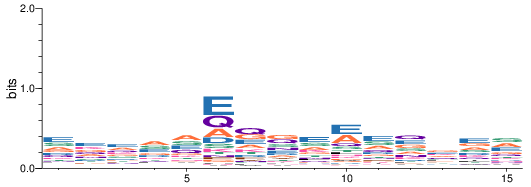

Supplement: S7 File — (ZIP) [file pcbi.1010238.s011.zip › disprot_html_table/logos/AVG_F102.png]

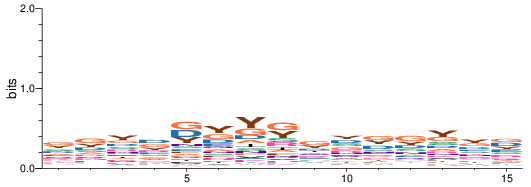

Supplement: S7 File — (ZIP) [file pcbi.1010238.s011.zip › disprot_html_table/logos/AVG_F103.png]

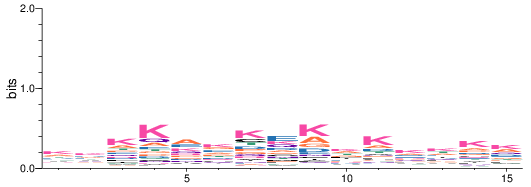

Supplement: S7 File — (ZIP) [file pcbi.1010238.s011.zip › disprot_html_table/logos/AVG_F104.png]

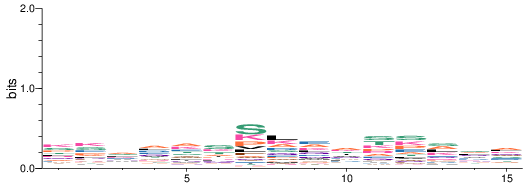

Supplement: S7 File — (ZIP) [file pcbi.1010238.s011.zip › disprot_html_table/logos/AVG_F105.png]

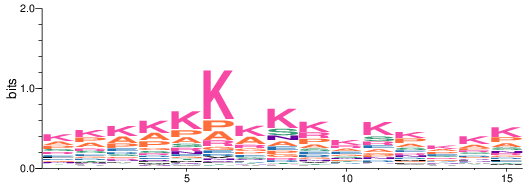

Supplement: S7 File — (ZIP) [file pcbi.1010238.s011.zip › disprot_html_table/logos/AVG_F106.png]

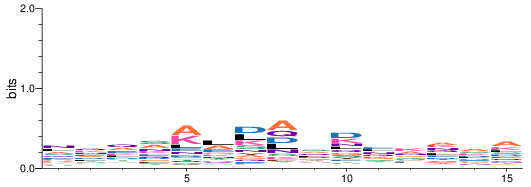

Supplement: S7 File — (ZIP) [file pcbi.1010238.s011.zip › disprot_html_table/logos/AVG_F107.png]

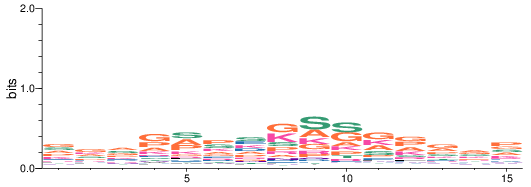

Supplement: S7 File — (ZIP) [file pcbi.1010238.s011.zip › disprot_html_table/logos/AVG_F108.png]

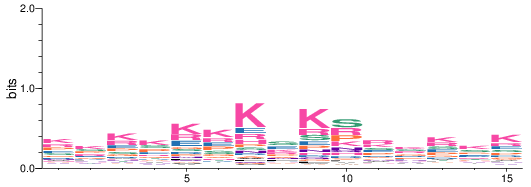

Supplement: S7 File — (ZIP) [file pcbi.1010238.s011.zip › disprot_html_table/logos/AVG_F109.png]

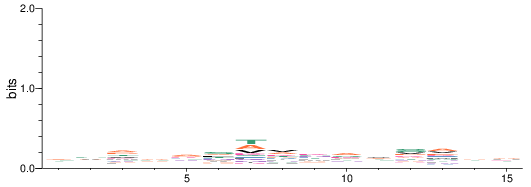

Supplement: S7 File — (ZIP) [file pcbi.1010238.s011.zip › disprot_html_table/logos/AVG_F11.png]

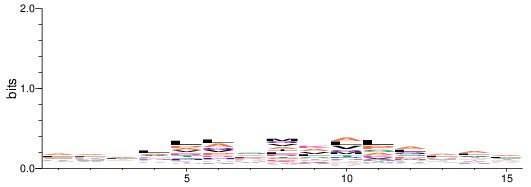

Supplement: S7 File — (ZIP) [file pcbi.1010238.s011.zip › disprot_html_table/logos/AVG_F110.png]

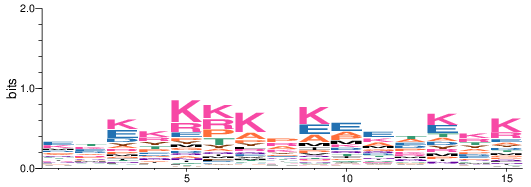

Supplement: S7 File — (ZIP) [file pcbi.1010238.s011.zip › disprot_html_table/logos/AVG_F111.png]

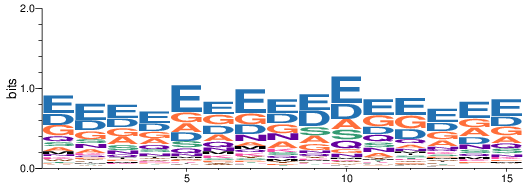

Supplement: S7 File — (ZIP) [file pcbi.1010238.s011.zip › disprot_html_table/logos/AVG_F112.png]

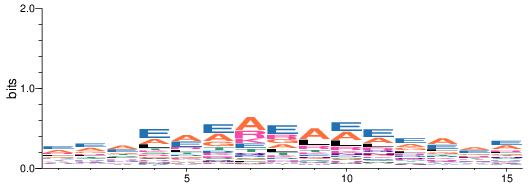

Supplement: S7 File — (ZIP) [file pcbi.1010238.s011.zip › disprot_html_table/logos/AVG_F113.png]

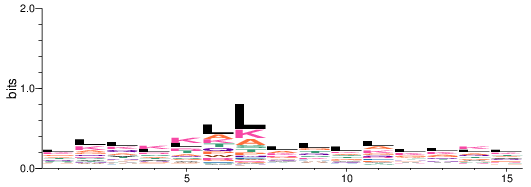

Supplement: S7 File — (ZIP) [file pcbi.1010238.s011.zip › disprot_html_table/logos/AVG_F114.png]

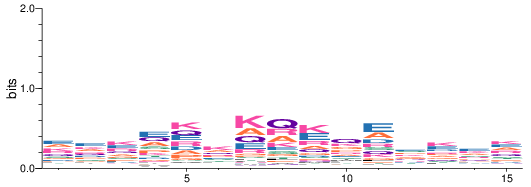

Supplement: S7 File — (ZIP) [file pcbi.1010238.s011.zip › disprot_html_table/logos/AVG_F115.png]

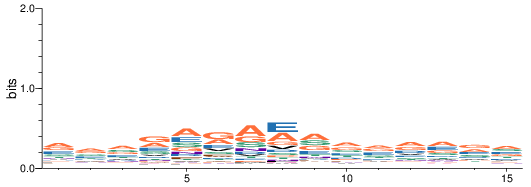

Supplement: S7 File — (ZIP) [file pcbi.1010238.s011.zip › disprot_html_table/logos/AVG_F116.png]

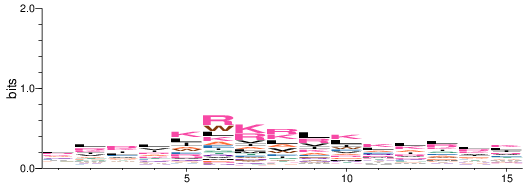

Supplement: S7 File — (ZIP) [file pcbi.1010238.s011.zip › disprot_html_table/logos/AVG_F117.png]

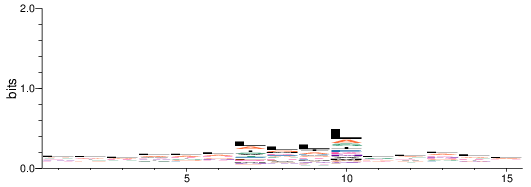

Supplement: S7 File — (ZIP) [file pcbi.1010238.s011.zip › disprot_html_table/logos/AVG_F118.png]

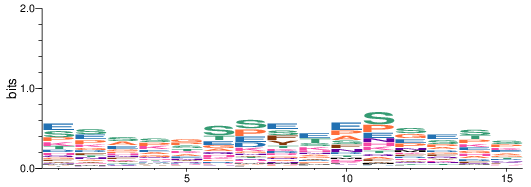

Supplement: S7 File — (ZIP) [file pcbi.1010238.s011.zip › disprot_html_table/logos/AVG_F119.png]

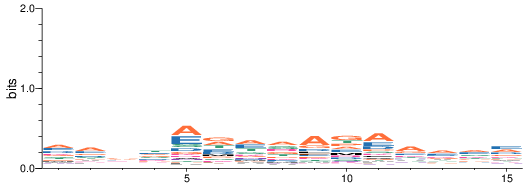

Supplement: S7 File — (ZIP) [file pcbi.1010238.s011.zip › disprot_html_table/logos/AVG_F12.png]

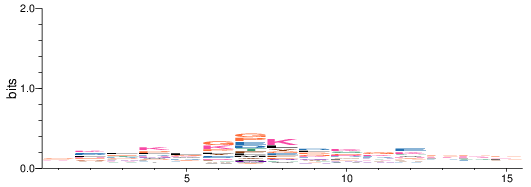

Supplement: S7 File — (ZIP) [file pcbi.1010238.s011.zip › disprot_html_table/logos/AVG_F120.png]

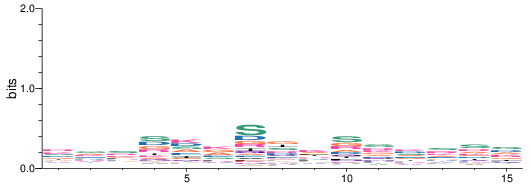

Supplement: S7 File — (ZIP) [file pcbi.1010238.s011.zip › disprot_html_table/logos/AVG_F121.png]

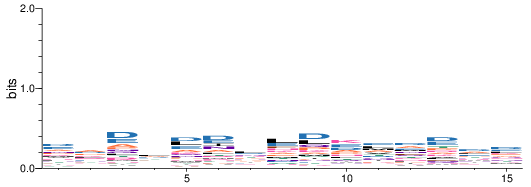

Supplement: S7 File — (ZIP) [file pcbi.1010238.s011.zip › disprot_html_table/logos/AVG_F122.png]

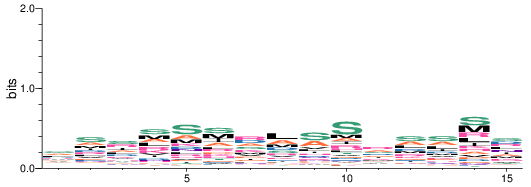

Supplement: S7 File — (ZIP) [file pcbi.1010238.s011.zip › disprot_html_table/logos/AVG_F123.png]

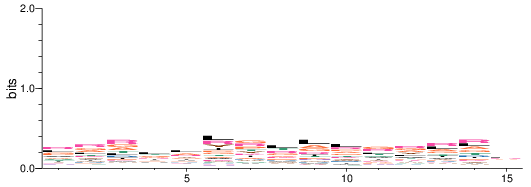

Supplement: S7 File — (ZIP) [file pcbi.1010238.s011.zip › disprot_html_table/logos/AVG_F124.png]

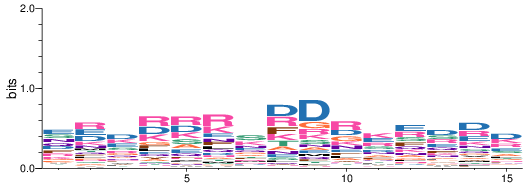

Supplement: S7 File — (ZIP) [file pcbi.1010238.s011.zip › disprot_html_table/logos/AVG_F125.png]

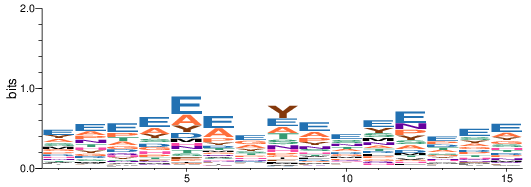

Supplement: S7 File — (ZIP) [file pcbi.1010238.s011.zip › disprot_html_table/logos/AVG_F126.png]

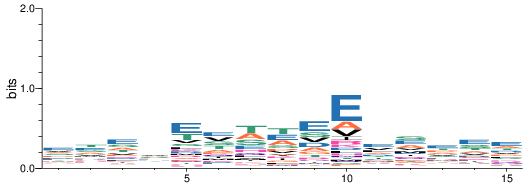

Supplement: S7 File — (ZIP) [file pcbi.1010238.s011.zip › disprot_html_table/logos/AVG_F127.png]

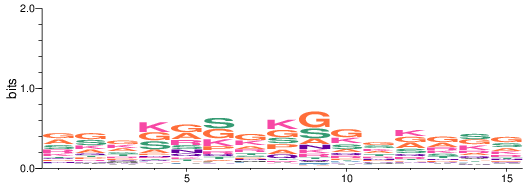

Supplement: S7 File — (ZIP) [file pcbi.1010238.s011.zip › disprot_html_table/logos/AVG_F128.png]

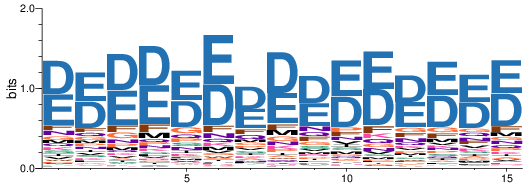

Supplement: S7 File — (ZIP) [file pcbi.1010238.s011.zip › disprot_html_table/logos/AVG_F129.png]

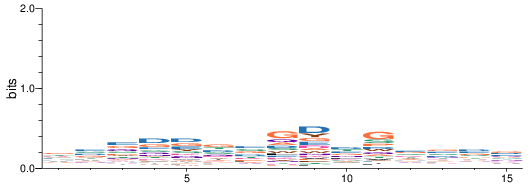

Supplement: S7 File — (ZIP) [file pcbi.1010238.s011.zip › disprot_html_table/logos/AVG_F13.png]

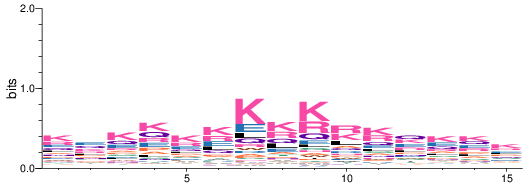

Supplement: S7 File — (ZIP) [file pcbi.1010238.s011.zip › disprot_html_table/logos/AVG_F130.png]

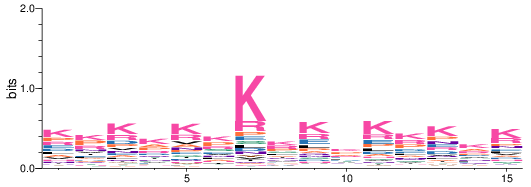

Supplement: S7 File — (ZIP) [file pcbi.1010238.s011.zip › disprot_html_table/logos/AVG_F131.png]

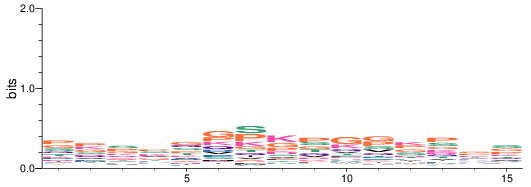

Supplement: S7 File — (ZIP) [file pcbi.1010238.s011.zip › disprot_html_table/logos/AVG_F132.png]

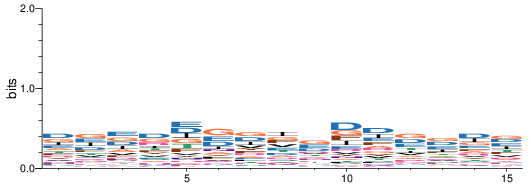

Supplement: S7 File — (ZIP) [file pcbi.1010238.s011.zip › disprot_html_table/logos/AVG_F133.png]

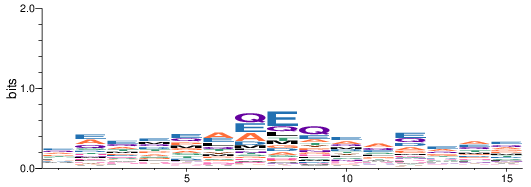

Supplement: S7 File — (ZIP) [file pcbi.1010238.s011.zip › disprot_html_table/logos/AVG_F134.png]

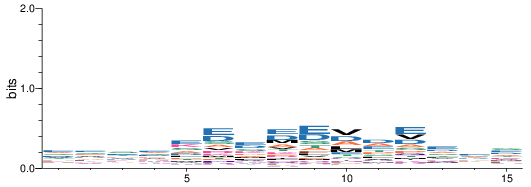

Supplement: S7 File — (ZIP) [file pcbi.1010238.s011.zip › disprot_html_table/logos/AVG_F135.png]

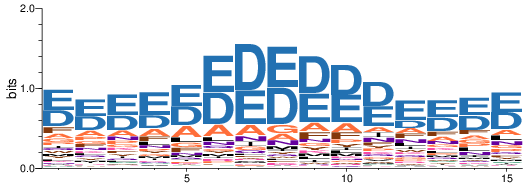

Supplement: S7 File — (ZIP) [file pcbi.1010238.s011.zip › disprot_html_table/logos/AVG_F136.png]

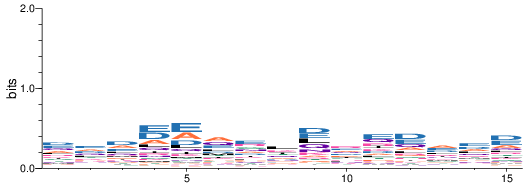

Supplement: S7 File — (ZIP) [file pcbi.1010238.s011.zip › disprot_html_table/logos/AVG_F137.png]

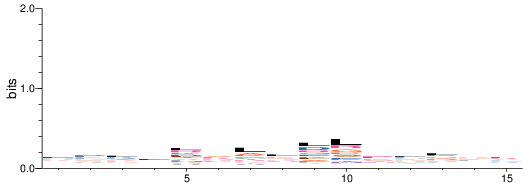

Supplement: S7 File — (ZIP) [file pcbi.1010238.s011.zip › disprot_html_table/logos/AVG_F138.png]

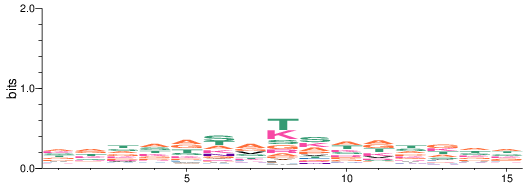

Supplement: S7 File — (ZIP) [file pcbi.1010238.s011.zip › disprot_html_table/logos/AVG_F139.png]

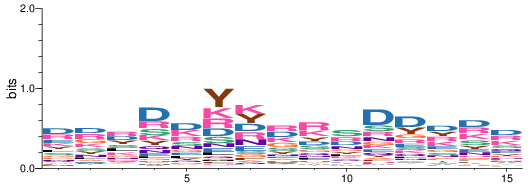

Supplement: S7 File — (ZIP) [file pcbi.1010238.s011.zip › disprot_html_table/logos/AVG_F14.png]

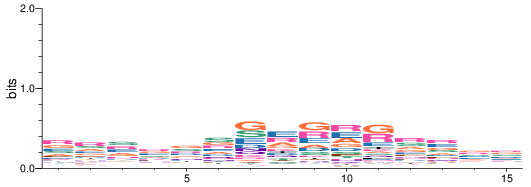

Supplement: S7 File — (ZIP) [file pcbi.1010238.s011.zip › disprot_html_table/logos/AVG_F140.png]

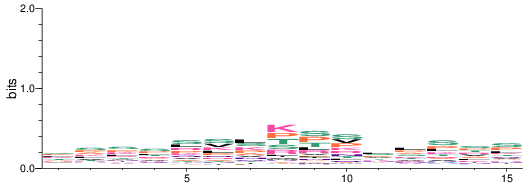

Supplement: S7 File — (ZIP) [file pcbi.1010238.s011.zip › disprot_html_table/logos/AVG_F141.png]

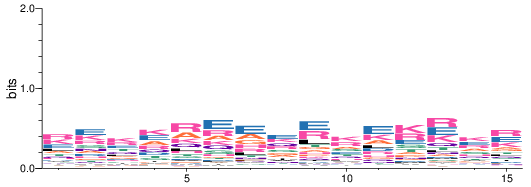

Supplement: S7 File — (ZIP) [file pcbi.1010238.s011.zip › disprot_html_table/logos/AVG_F142.png]

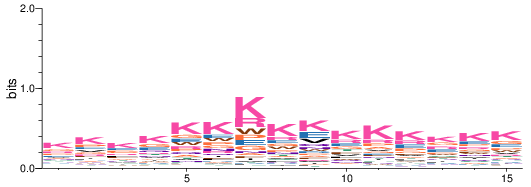

Supplement: S7 File — (ZIP) [file pcbi.1010238.s011.zip › disprot_html_table/logos/AVG_F143.png]

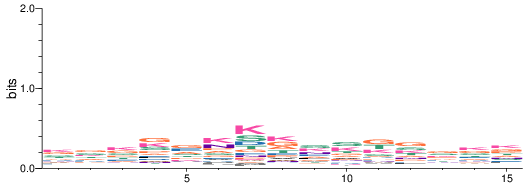

Supplement: S7 File — (ZIP) [file pcbi.1010238.s011.zip › disprot_html_table/logos/AVG_F144.png]

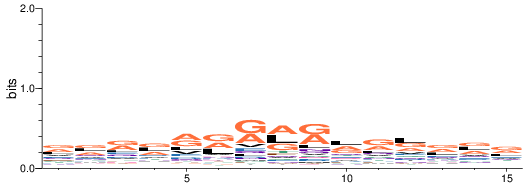

Supplement: S7 File — (ZIP) [file pcbi.1010238.s011.zip › disprot_html_table/logos/AVG_F145.png]

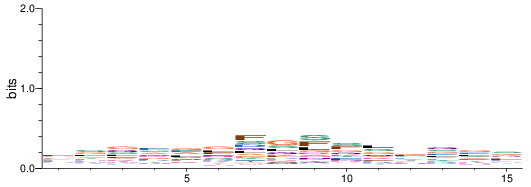

Supplement: S7 File — (ZIP) [file pcbi.1010238.s011.zip › disprot_html_table/logos/AVG_F146.png]

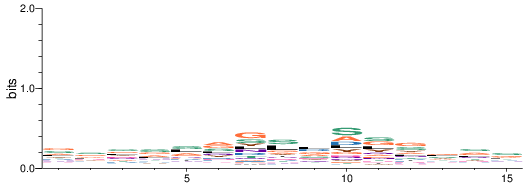

Supplement: S7 File — (ZIP) [file pcbi.1010238.s011.zip › disprot_html_table/logos/AVG_F147.png]

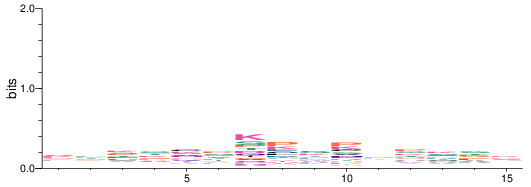

Supplement: S7 File — (ZIP) [file pcbi.1010238.s011.zip › disprot_html_table/logos/AVG_F148.png]

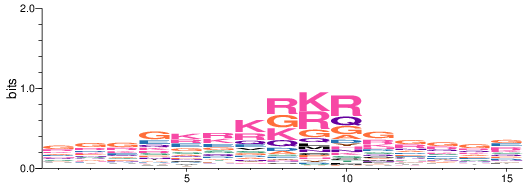

Supplement: S7 File — (ZIP) [file pcbi.1010238.s011.zip › disprot_html_table/logos/AVG_F149.png]

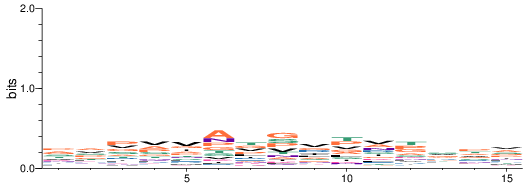

Supplement: S7 File — (ZIP) [file pcbi.1010238.s011.zip › disprot_html_table/logos/AVG_F15.png]

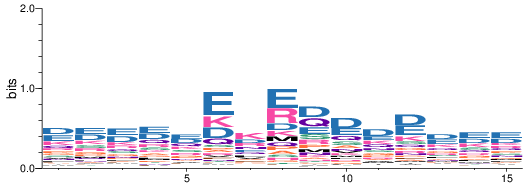

Supplement: S7 File — (ZIP) [file pcbi.1010238.s011.zip › disprot_html_table/logos/AVG_F150.png]

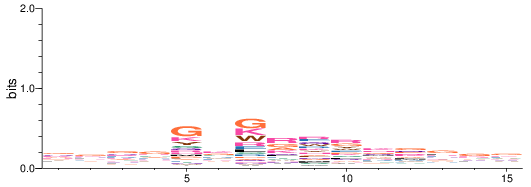

Supplement: S7 File — (ZIP) [file pcbi.1010238.s011.zip › disprot_html_table/logos/AVG_F151.png]

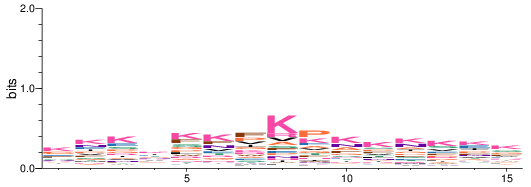

Supplement: S7 File — (ZIP) [file pcbi.1010238.s011.zip › disprot_html_table/logos/AVG_F152.png]

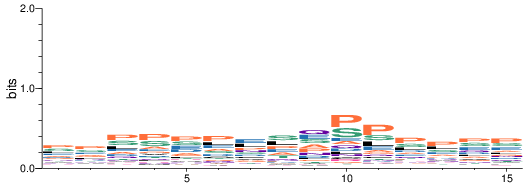

Supplement: S7 File — (ZIP) [file pcbi.1010238.s011.zip › disprot_html_table/logos/AVG_F153.png]

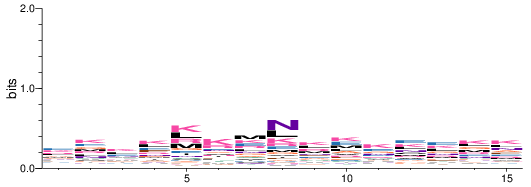

Supplement: S7 File — (ZIP) [file pcbi.1010238.s011.zip › disprot_html_table/logos/AVG_F154.png]

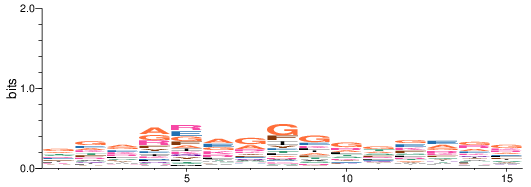

Supplement: S7 File — (ZIP) [file pcbi.1010238.s011.zip › disprot_html_table/logos/AVG_F155.png]

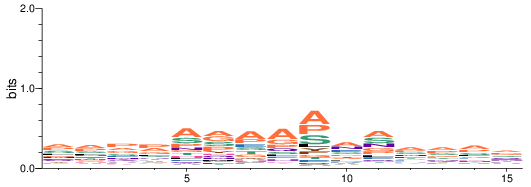

Supplement: S7 File — (ZIP) [file pcbi.1010238.s011.zip › disprot_html_table/logos/AVG_F156.png]

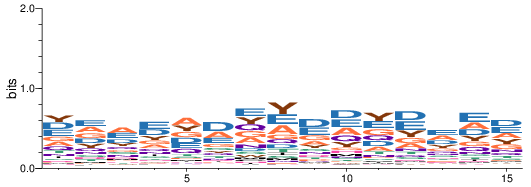

Supplement: S7 File — (ZIP) [file pcbi.1010238.s011.zip › disprot_html_table/logos/AVG_F157.png]

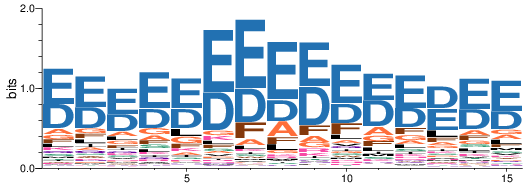

Supplement: S7 File — (ZIP) [file pcbi.1010238.s011.zip › disprot_html_table/logos/AVG_F158.png]

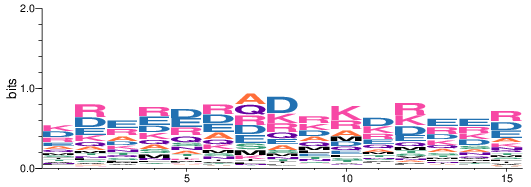

Supplement: S7 File — (ZIP) [file pcbi.1010238.s011.zip › disprot_html_table/logos/AVG_F159.png]

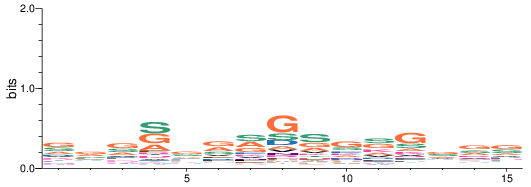

Supplement: S7 File — (ZIP) [file pcbi.1010238.s011.zip › disprot_html_table/logos/AVG_F16.png]

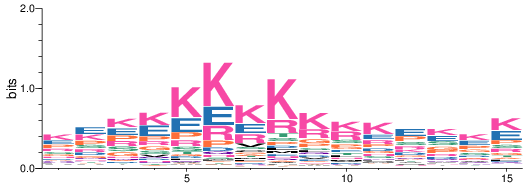

Supplement: S7 File — (ZIP) [file pcbi.1010238.s011.zip › disprot_html_table/logos/AVG_F160.png]

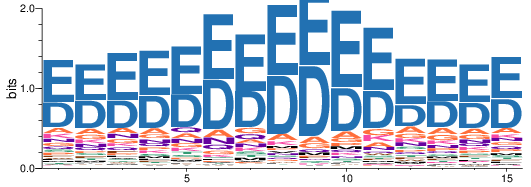

Supplement: S7 File — (ZIP) [file pcbi.1010238.s011.zip › disprot_html_table/logos/AVG_F161.png]

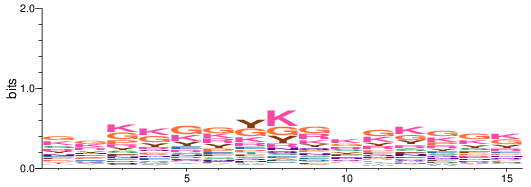

Supplement: S7 File — (ZIP) [file pcbi.1010238.s011.zip › disprot_html_table/logos/AVG_F162.png]

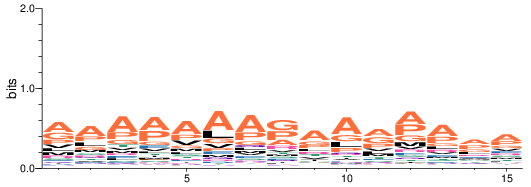

Supplement: S7 File — (ZIP) [file pcbi.1010238.s011.zip › disprot_html_table/logos/AVG_F163.png]

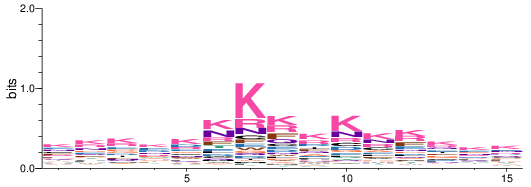

Supplement: S7 File — (ZIP) [file pcbi.1010238.s011.zip › disprot_html_table/logos/AVG_F164.png]

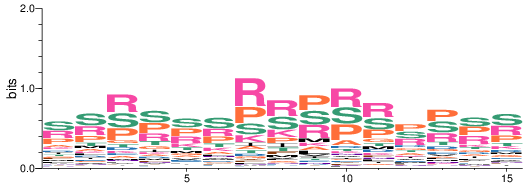

Supplement: S7 File — (ZIP) [file pcbi.1010238.s011.zip › disprot_html_table/logos/AVG_F165.png]

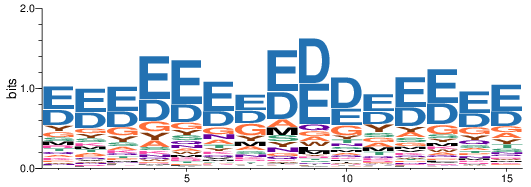

Supplement: S7 File — (ZIP) [file pcbi.1010238.s011.zip › disprot_html_table/logos/AVG_F166.png]

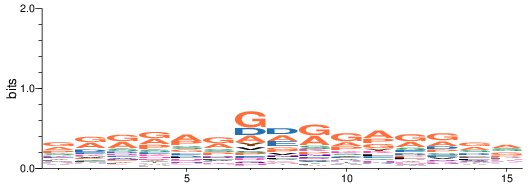

Supplement: S7 File — (ZIP) [file pcbi.1010238.s011.zip › disprot_html_table/logos/AVG_F167.png]

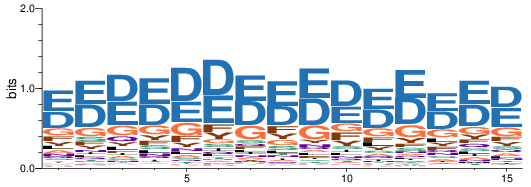

Supplement: S7 File — (ZIP) [file pcbi.1010238.s011.zip › disprot_html_table/logos/AVG_F168.png]

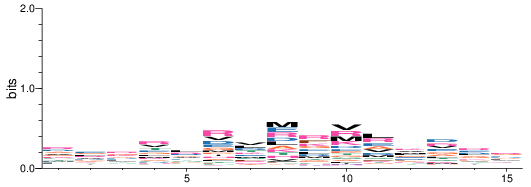

Supplement: S7 File — (ZIP) [file pcbi.1010238.s011.zip › disprot_html_table/logos/AVG_F169.png]

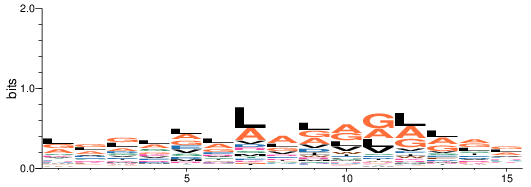

Supplement: S7 File — (ZIP) [file pcbi.1010238.s011.zip › disprot_html_table/logos/AVG_F17.png]

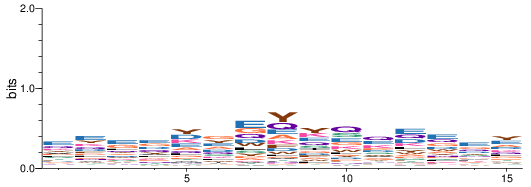

Supplement: S7 File — (ZIP) [file pcbi.1010238.s011.zip › disprot_html_table/logos/AVG_F170.png]

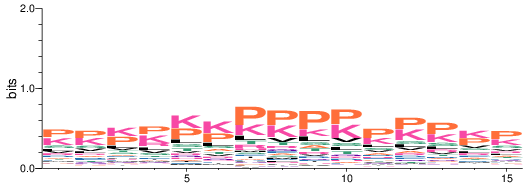

Supplement: S7 File — (ZIP) [file pcbi.1010238.s011.zip › disprot_html_table/logos/AVG_F171.png]

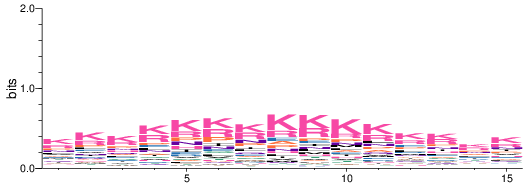

Supplement: S7 File — (ZIP) [file pcbi.1010238.s011.zip › disprot_html_table/logos/AVG_F172.png]

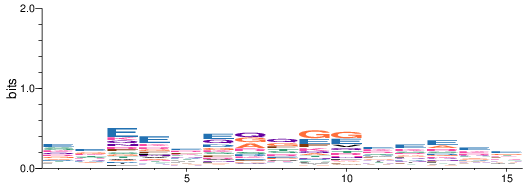

Supplement: S7 File — (ZIP) [file pcbi.1010238.s011.zip › disprot_html_table/logos/AVG_F173.png]

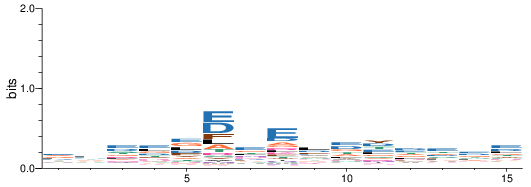

Supplement: S7 File — (ZIP) [file pcbi.1010238.s011.zip › disprot_html_table/logos/AVG_F174.png]

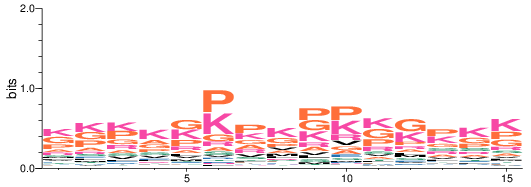

Supplement: S7 File — (ZIP) [file pcbi.1010238.s011.zip › disprot_html_table/logos/AVG_F175.png]

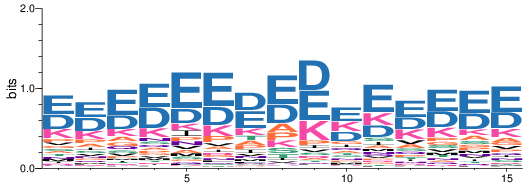

Supplement: S7 File — (ZIP) [file pcbi.1010238.s011.zip › disprot_html_table/logos/AVG_F176.png]

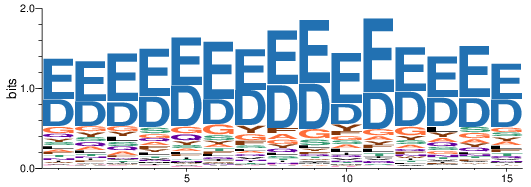

Supplement: S7 File — (ZIP) [file pcbi.1010238.s011.zip › disprot_html_table/logos/AVG_F177.png]

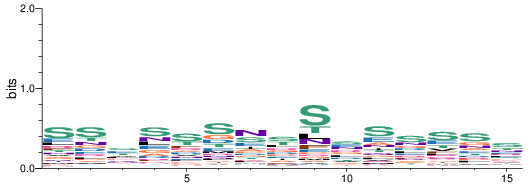

Supplement: S7 File — (ZIP) [file pcbi.1010238.s011.zip › disprot_html_table/logos/AVG_F178.png]

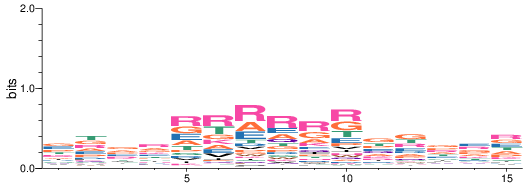

Supplement: S7 File — (ZIP) [file pcbi.1010238.s011.zip › disprot_html_table/logos/AVG_F179.png]

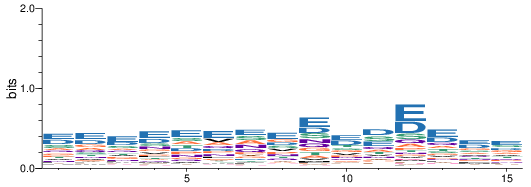

Supplement: S7 File — (ZIP) [file pcbi.1010238.s011.zip › disprot_html_table/logos/AVG_F18.png]

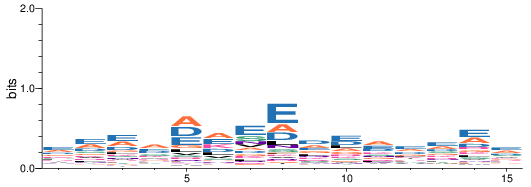

Supplement: S7 File — (ZIP) [file pcbi.1010238.s011.zip › disprot_html_table/logos/AVG_F180.png]

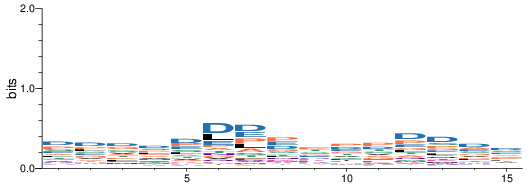

Supplement: S7 File — (ZIP) [file pcbi.1010238.s011.zip › disprot_html_table/logos/AVG_F181.png]

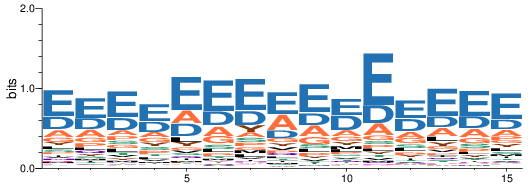

Supplement: S7 File — (ZIP) [file pcbi.1010238.s011.zip › disprot_html_table/logos/AVG_F182.png]

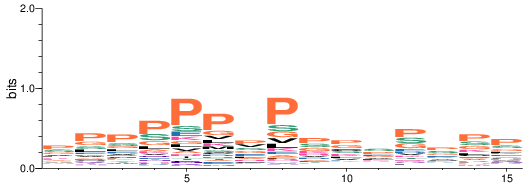

Supplement: S7 File — (ZIP) [file pcbi.1010238.s011.zip › disprot_html_table/logos/AVG_F183.png]

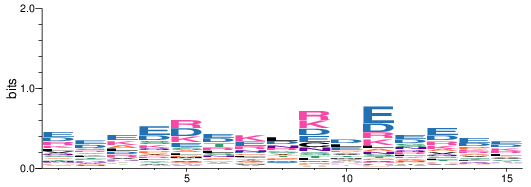

Supplement: S7 File — (ZIP) [file pcbi.1010238.s011.zip › disprot_html_table/logos/AVG_F184.png]
