# Supplementary material for: Discovering molecular features of intrinsically disordered regions by using evolution for contrastive learning
Source: PLoS Comput Biol. 2022 Jun 29;18(6):e1010238. doi: 10.1371/journal.pcbi.1010238 (PMC9275697; doi:10.1371/journal.pcbi.1010238)
Supplement: S7 File — (ZIP) [file pcbi.1010238.s011.zip › disprot_html_table/disprot_table.html]

| Feature | Max Logo | Average Logo |
| --- | --- | --- |
| **Feature 58** |  |  |
| **Feature 24** |  |  |
| **Feature 21** |  |  |
| **Feature 46** |  |  |
| **Feature 19** |  |  |
| **Feature 1** |  |  |
| **Feature 66** |  |  |
| **Feature 107** |  |  |
| **Feature 203** |  |  |
| **Feature 33** |  |  |
| **Feature 67** |  |  |
| **Feature 110** |  |  |
| **Feature 11** |  |  |
| **Feature 54** |  |  |
| **Feature 15** |  |  |
| **Feature 240** |  |  |
| **Feature 38** |  |  |
| **Feature 42** |  |  |
| **Feature 114** |  |  |
| **Feature 17** |  |  |
| **Feature 77** |  |  |
| **Feature 145** |  |  |
| **Feature 113** |  |  |
| **Feature 45** |  |  |
| **Feature 221** |  |  |
| **Feature 233** |  |  |
| **Feature 8** |  |  |
| **Feature 118** |  |  |
| **Feature 252** |  |  |
| **Feature 147** |  |  |
| **Feature 138** |  |  |
| **Feature 120** |  |  |
| **Feature 155** |  |  |
| **Feature 124** |  |  |
| **Feature 224** |  |  |
| **Feature 169** |  |  |
| **Feature 183** |  |  |
| **Feature 212** |  |  |
| **Feature 198** |  |  |
| **Feature 97** |  |  |
| **Feature 156** |  |  |
| **Feature 40** |  |  |
| **Feature 16** |  |  |
| **Feature 236** |  |  |
| **Feature 187** |  |  |
| **Feature 108** |  |  |
| **Feature 167** |  |  |
| **Feature 49** |  |  |
| **Feature 86** |  |  |
| **Feature 56** |  |  |
| **Feature 75** |  |  |
| **Feature 223** |  |  |
| **Feature 52** |  |  |
| **Feature 235** |  |  |
| **Feature 79** |  |  |
| **Feature 163** |  |  |
| **Feature 225** |  |  |
| **Feature 219** |  |  |
| **Feature 139** |  |  |
| **Feature 209** |  |  |
| **Feature 64** |  |  |
| **Feature 207** |  |  |
| **Feature 153** |  |  |
| **Feature 181** |  |  |
| **Feature 146** |  |  |
| **Feature 246** |  |  |
| **Feature 10** |  |  |
| **Feature 216** |  |  |
| **Feature 85** |  |  |
| **Feature 166** |  |  |
| **Feature 25** |  |  |
| **Feature 27** |  |  |
| **Feature 81** |  |  |
| **Feature 39** |  |  |
| **Feature 84** |  |  |
| **Feature 63** |  |  |
| **Feature 127** |  |  |
| **Feature 50** |  |  |
| **Feature 122** |  |  |
| **Feature 135** |  |  |
| **Feature 76** |  |  |
| **Feature 189** |  |  |
| **Feature 18** |  |  |
| **Feature 126** |  |  |
| **Feature 157** |  |  |
| **Feature 92** |  |  |
| **Feature 218** |  |  |
| **Feature 4** |  |  |
| **Feature 74** |  |  |
| **Feature 2** |  |  |
| **Feature 34** |  |  |
| **Feature 161** |  |  |
| **Feature 231** |  |  |
| **Feature 5** |  |  |
| **Feature 251** |  |  |
| **Feature 22** |  |  |
| **Feature 72** |  |  |
| **Feature 191** |  |  |
| **Feature 213** |  |  |
| **Feature 80** |  |  |
| **Feature 177** |  |  |
| **Feature 129** |  |  |
| **Feature 210** |  |  |
| **Feature 44** |  |  |
| **Feature 158** |  |  |
| **Feature 136** |  |  |
| **Feature 182** |  |  |
| **Feature 200** |  |  |
| **Feature 73** |  |  |
| **Feature 239** |  |  |
| **Feature 249** |  |  |
| **Feature 180** |  |  |
| **Feature 96** |  |  |
| **Feature 220** |  |  |
| **Feature 244** |  |  |
| **Feature 103** |  |  |
| **Feature 14** |  |  |
| **Feature 254** |  |  |
| **Feature 174** |  |  |
| **Feature 133** |  |  |
| **Feature 168** |  |  |
| **Feature 242** |  |  |
| **Feature 12** |  |  |
| **Feature 82** |  |  |
| **Feature 193** |  |  |
| **Feature 89** |  |  |
| **Feature 119** |  |  |
| **Feature 196** |  |  |
| **Feature 83** |  |  |
| **Feature 116** |  |  |
| **Feature 215** |  |  |
| **Feature 112** |  |  |
| **Feature 36** |  |  |
| **Feature 211** |  |  |
| **Feature 195** |  |  |
| **Feature 100** |  |  |
| **Feature 98** |  |  |
| **Feature 104** |  |  |
| **Feature 71** |  |  |
| **Feature 154** |  |  |
| **Feature 87** |  |  |
| **Feature 70** |  |  |
| **Feature 111** |  |  |
| **Feature 9** |  |  |
| **Feature 93** |  |  |
| **Feature 90** |  |  |
| **Feature 106** |  |  |
| **Feature 29** |  |  |
| **Feature 26** |  |  |
| **Feature 229** |  |  |
| **Feature 51** |  |  |
| **Feature 131** |  |  |
| **Feature 109** |  |  |
| **Feature 59** |  |  |
| **Feature 91** |  |  |
| **Feature 117** |  |  |
| **Feature 130** |  |  |
| **Feature 172** |  |  |
| **Feature 164** |  |  |
| **Feature 160** |  |  |
| **Feature 192** |  |  |
| **Feature 48** |  |  |
| **Feature 149** |  |  |
| **Feature 162** |  |  |
| **Feature 30** |  |  |
| **Feature 238** |  |  |
| **Feature 143** |  |  |
| **Feature 185** |  |  |
| **Feature 78** |  |  |
| **Feature 99** |  |  |
| **Feature 57** |  |  |
| **Feature 125** |  |  |
| **Feature 43** |  |  |
| **Feature 53** |  |  |
| **Feature 190** |  |  |
| **Feature 37** |  |  |
| **Feature 69** |  |  |
| **Feature 227** |  |  |
| **Feature 150** |  |  |
| **Feature 201** |  |  |
| **Feature 184** |  |  |
| **Feature 115** |  |  |
| **Feature 7** |  |  |
| **Feature 248** |  |  |
| **Feature 41** |  |  |
| **Feature 206** |  |  |
| **Feature 55** |  |  |
| **Feature 35** |  |  |
| **Feature 204** |  |  |
| **Feature 199** |  |  |
| **Feature 137** |  |  |
| **Feature 159** |  |  |
| **Feature 102** |  |  |
| **Feature 134** |  |  |
| **Feature 228** |  |  |
| **Feature 3** |  |  |
| **Feature 47** |  |  |
| **Feature 217** |  |  |
| **Feature 20** |  |  |
| **Feature 176** |  |  |
| **Feature 208** |  |  |
| **Feature 60** |  |  |
| **Feature 250** |  |  |
| **Feature 170** |  |  |
| **Feature 253** |  |  |
| **Feature 230** |  |  |
| **Feature 142** |  |  |
| **Feature 0** |  |  |
| **Feature 31** |  |  |
| **Feature 23** |  |  |
| **Feature 28** |  |  |
| **Feature 68** |  |  |
| **Feature 186** |  |  |
| **Feature 234** |  |  |
| **Feature 226** |  |  |
| **Feature 32** |  |  |
| **Feature 128** |  |  |
| **Feature 132** |  |  |
| **Feature 148** |  |  |
| **Feature 237** |  |  |
| **Feature 94** |  |  |
| **Feature 141** |  |  |
| **Feature 88** |  |  |
| **Feature 171** |  |  |
| **Feature 165** |  |  |
| **Feature 247** |  |  |
| **Feature 175** |  |  |
| **Feature 152** |  |  |
| **Feature 214** |  |  |
| **Feature 62** |  |  |
| **Feature 179** |  |  |
| **Feature 202** |  |  |
| **Feature 95** |  |  |
| **Feature 245** |  |  |
| **Feature 188** |  |  |
| **Feature 232** |  |  |
| **Feature 243** |  |  |
| **Feature 105** |  |  |
| **Feature 144** |  |  |
| **Feature 123** |  |  |
| **Feature 222** |  |  |
| **Feature 255** |  |  |
| **Feature 6** |  |  |
| **Feature 121** |  |  |
| **Feature 61** |  |  |
| **Feature 178** |  |  |
| **Feature 140** |  |  |
| **Feature 65** |  |  |
| **Feature 101** |  |  |
| **Feature 205** |  |  |
| **Feature 241** |  |  |
| **Feature 13** |  |  |
| **Feature 151** |  |  |
| **Feature 173** |  |  |
| **Feature 194** |  |  |
| **Feature 197** |  |  |
