# Supplementary figures and images for: Discovering molecular features of intrinsically disordered regions by using evolution for contrastive learning (part 3 of 3)
Source: PLoS Comput Biol. 2022 Jun 29;18(6):e1010238. doi: 10.1371/journal.pcbi.1010238 (PMC9275697; doi:10.1371/journal.pcbi.1010238)

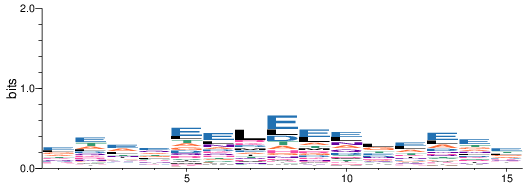

Supplement: S7 File — (ZIP) [file pcbi.1010238.s011.zip › disprot_html_table/logos/AVG_F45.png]

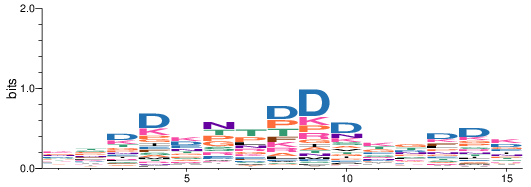

Supplement: S7 File — (ZIP) [file pcbi.1010238.s011.zip › disprot_html_table/logos/AVG_F46.png]

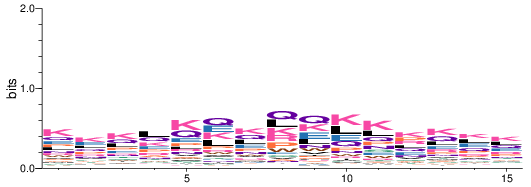

Supplement: S7 File — (ZIP) [file pcbi.1010238.s011.zip › disprot_html_table/logos/AVG_F47.png]
