# Supplementary figures and images for: Discovering molecular features of intrinsically disordered regions by using evolution for contrastive learning (part 2 of 3)
Source: PLoS Comput Biol. 2022 Jun 29;18(6):e1010238. doi: 10.1371/journal.pcbi.1010238 (PMC9275697; doi:10.1371/journal.pcbi.1010238)

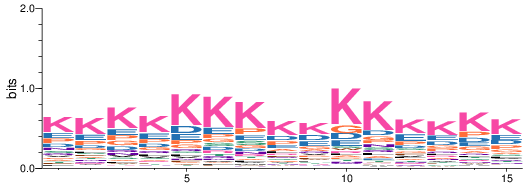

Supplement: S7 File — (ZIP) [file pcbi.1010238.s011.zip › disprot_html_table/logos/AVG_F185.png]

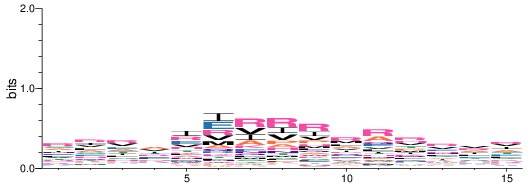

Supplement: S7 File — (ZIP) [file pcbi.1010238.s011.zip › disprot_html_table/logos/AVG_F186.png]

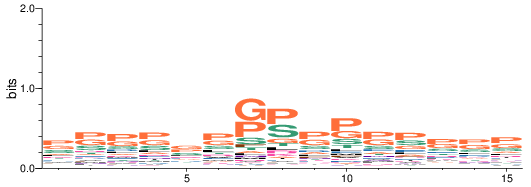

Supplement: S7 File — (ZIP) [file pcbi.1010238.s011.zip › disprot_html_table/logos/AVG_F187.png]

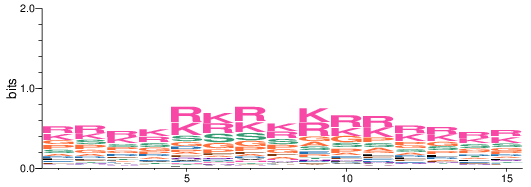

Supplement: S7 File — (ZIP) [file pcbi.1010238.s011.zip › disprot_html_table/logos/AVG_F188.png]

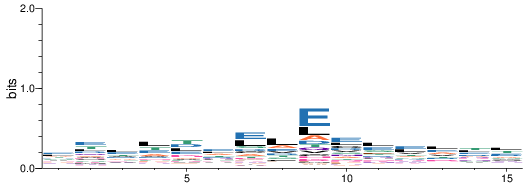

Supplement: S7 File — (ZIP) [file pcbi.1010238.s011.zip › disprot_html_table/logos/AVG_F189.png]

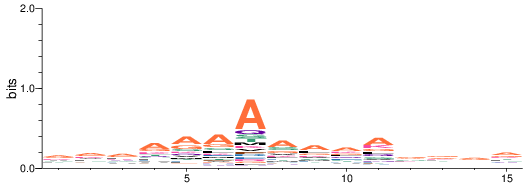

Supplement: S7 File — (ZIP) [file pcbi.1010238.s011.zip › disprot_html_table/logos/AVG_F19.png]

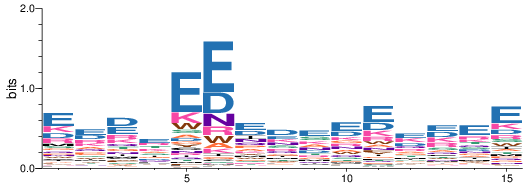

Supplement: S7 File — (ZIP) [file pcbi.1010238.s011.zip › disprot_html_table/logos/AVG_F190.png]

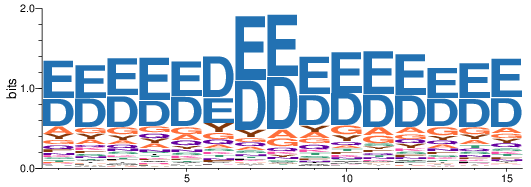

Supplement: S7 File — (ZIP) [file pcbi.1010238.s011.zip › disprot_html_table/logos/AVG_F191.png]

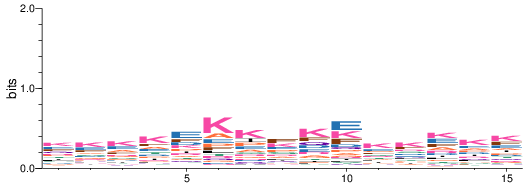

Supplement: S7 File — (ZIP) [file pcbi.1010238.s011.zip › disprot_html_table/logos/AVG_F192.png]

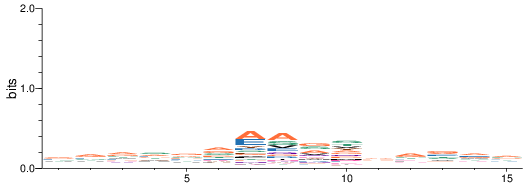

Supplement: S7 File — (ZIP) [file pcbi.1010238.s011.zip › disprot_html_table/logos/AVG_F193.png]

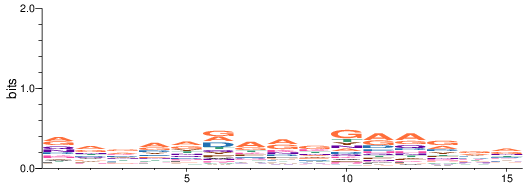

Supplement: S7 File — (ZIP) [file pcbi.1010238.s011.zip › disprot_html_table/logos/AVG_F194.png]

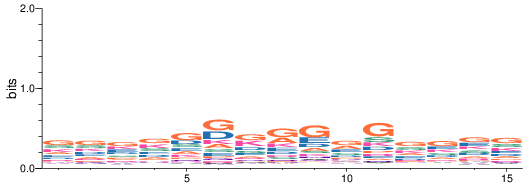

Supplement: S7 File — (ZIP) [file pcbi.1010238.s011.zip › disprot_html_table/logos/AVG_F195.png]

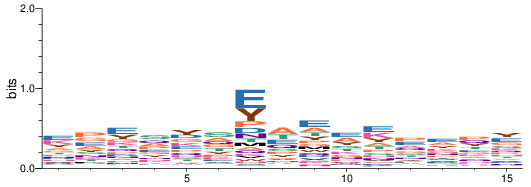

Supplement: S7 File — (ZIP) [file pcbi.1010238.s011.zip › disprot_html_table/logos/AVG_F196.png]

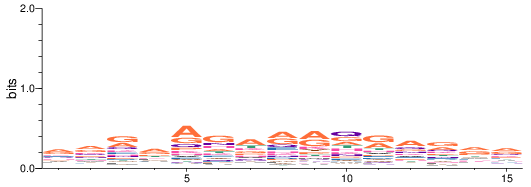

Supplement: S7 File — (ZIP) [file pcbi.1010238.s011.zip › disprot_html_table/logos/AVG_F197.png]

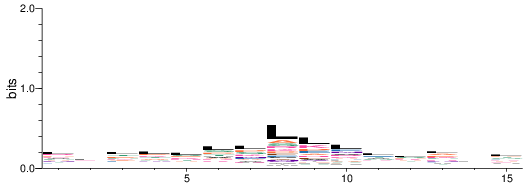

Supplement: S7 File — (ZIP) [file pcbi.1010238.s011.zip › disprot_html_table/logos/AVG_F198.png]

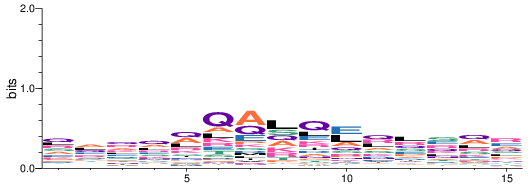

Supplement: S7 File — (ZIP) [file pcbi.1010238.s011.zip › disprot_html_table/logos/AVG_F199.png]

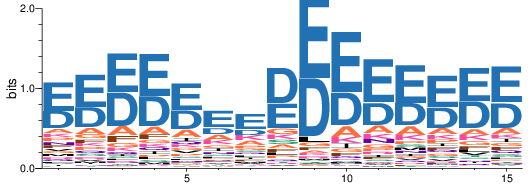

Supplement: S7 File — (ZIP) [file pcbi.1010238.s011.zip › disprot_html_table/logos/AVG_F2.png]

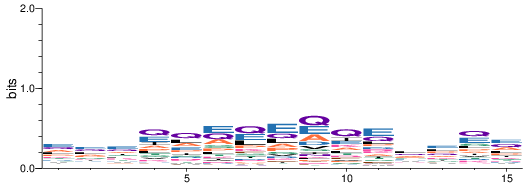

Supplement: S7 File — (ZIP) [file pcbi.1010238.s011.zip › disprot_html_table/logos/AVG_F20.png]

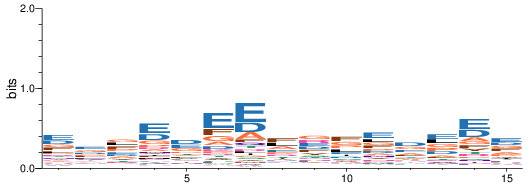

Supplement: S7 File — (ZIP) [file pcbi.1010238.s011.zip › disprot_html_table/logos/AVG_F200.png]

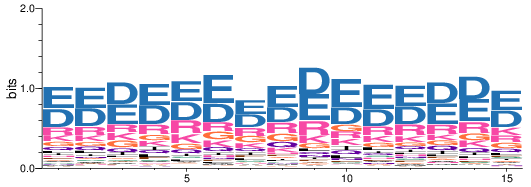

Supplement: S7 File — (ZIP) [file pcbi.1010238.s011.zip › disprot_html_table/logos/AVG_F201.png]

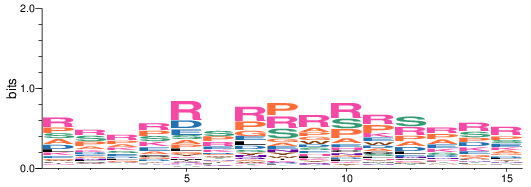

Supplement: S7 File — (ZIP) [file pcbi.1010238.s011.zip › disprot_html_table/logos/AVG_F202.png]

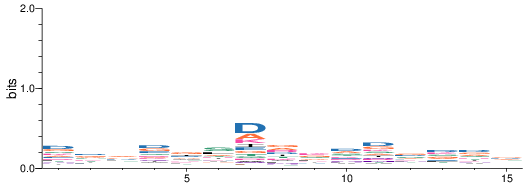

Supplement: S7 File — (ZIP) [file pcbi.1010238.s011.zip › disprot_html_table/logos/AVG_F203.png]

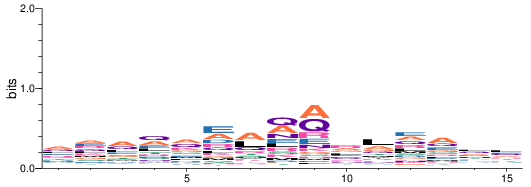

Supplement: S7 File — (ZIP) [file pcbi.1010238.s011.zip › disprot_html_table/logos/AVG_F204.png]

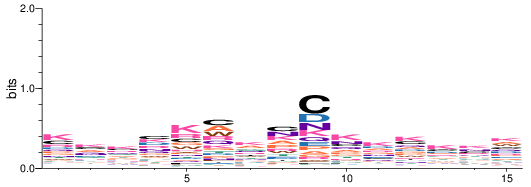

Supplement: S7 File — (ZIP) [file pcbi.1010238.s011.zip › disprot_html_table/logos/AVG_F205.png]

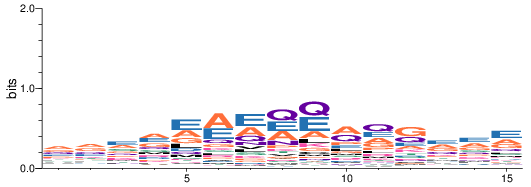

Supplement: S7 File — (ZIP) [file pcbi.1010238.s011.zip › disprot_html_table/logos/AVG_F206.png]

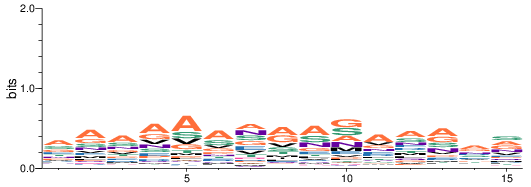

Supplement: S7 File — (ZIP) [file pcbi.1010238.s011.zip › disprot_html_table/logos/AVG_F207.png]

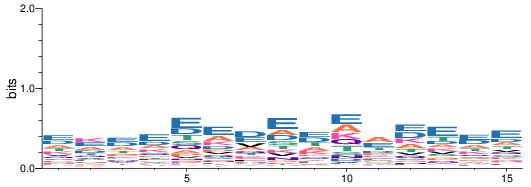

Supplement: S7 File — (ZIP) [file pcbi.1010238.s011.zip › disprot_html_table/logos/AVG_F208.png]

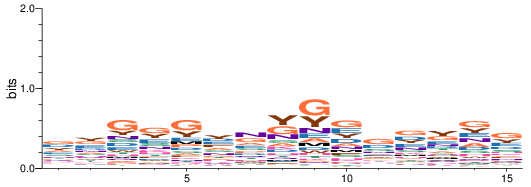

Supplement: S7 File — (ZIP) [file pcbi.1010238.s011.zip › disprot_html_table/logos/AVG_F209.png]

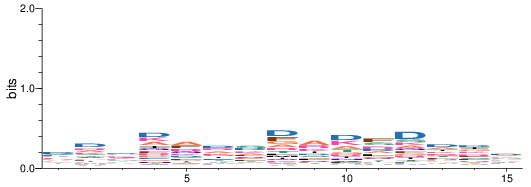

Supplement: S7 File — (ZIP) [file pcbi.1010238.s011.zip › disprot_html_table/logos/AVG_F21.png]

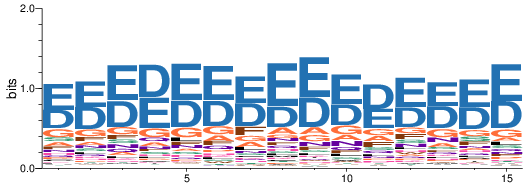

Supplement: S7 File — (ZIP) [file pcbi.1010238.s011.zip › disprot_html_table/logos/AVG_F210.png]

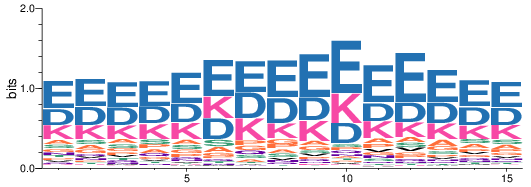

Supplement: S7 File — (ZIP) [file pcbi.1010238.s011.zip › disprot_html_table/logos/AVG_F211.png]

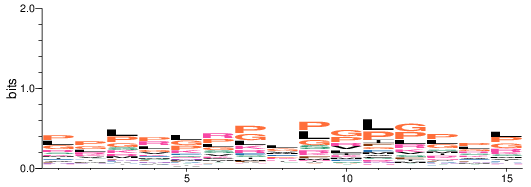

Supplement: S7 File — (ZIP) [file pcbi.1010238.s011.zip › disprot_html_table/logos/AVG_F212.png]

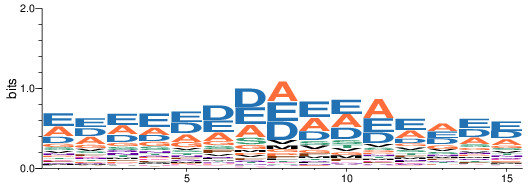

Supplement: S7 File — (ZIP) [file pcbi.1010238.s011.zip › disprot_html_table/logos/AVG_F213.png]

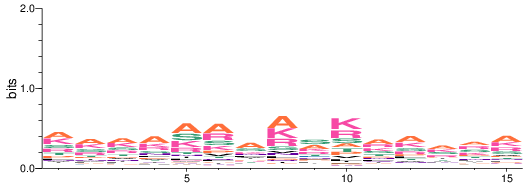

Supplement: S7 File — (ZIP) [file pcbi.1010238.s011.zip › disprot_html_table/logos/AVG_F214.png]

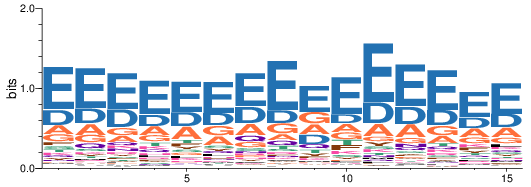

Supplement: S7 File — (ZIP) [file pcbi.1010238.s011.zip › disprot_html_table/logos/AVG_F215.png]

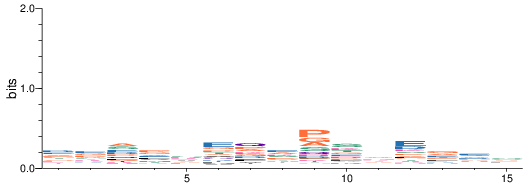

Supplement: S7 File — (ZIP) [file pcbi.1010238.s011.zip › disprot_html_table/logos/AVG_F216.png]

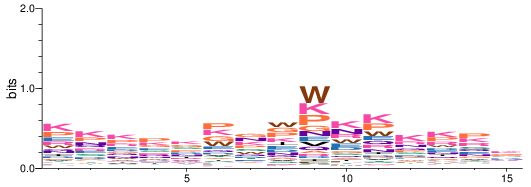

Supplement: S7 File — (ZIP) [file pcbi.1010238.s011.zip › disprot_html_table/logos/AVG_F217.png]

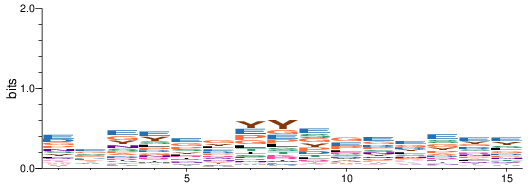

Supplement: S7 File — (ZIP) [file pcbi.1010238.s011.zip › disprot_html_table/logos/AVG_F218.png]

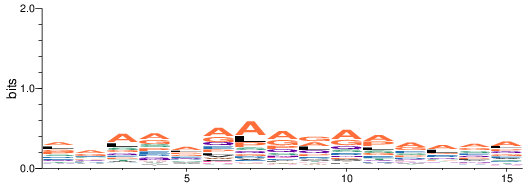

Supplement: S7 File — (ZIP) [file pcbi.1010238.s011.zip › disprot_html_table/logos/AVG_F219.png]

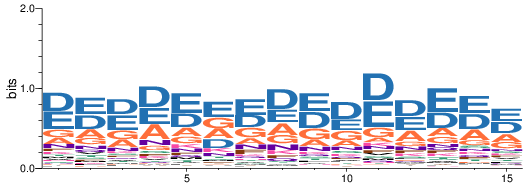

Supplement: S7 File — (ZIP) [file pcbi.1010238.s011.zip › disprot_html_table/logos/AVG_F22.png]

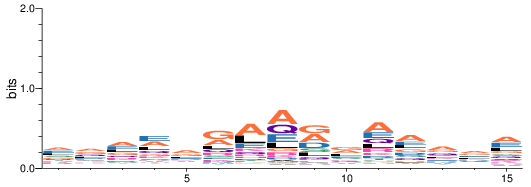

Supplement: S7 File — (ZIP) [file pcbi.1010238.s011.zip › disprot_html_table/logos/AVG_F220.png]

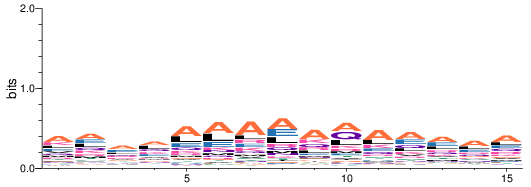

Supplement: S7 File — (ZIP) [file pcbi.1010238.s011.zip › disprot_html_table/logos/AVG_F221.png]

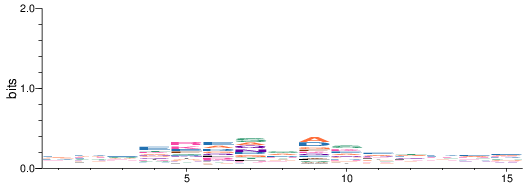

Supplement: S7 File — (ZIP) [file pcbi.1010238.s011.zip › disprot_html_table/logos/AVG_F222.png]

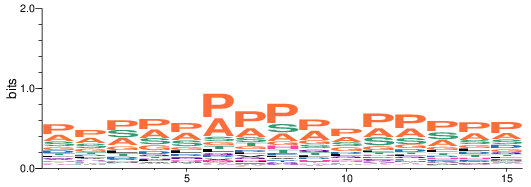

Supplement: S7 File — (ZIP) [file pcbi.1010238.s011.zip › disprot_html_table/logos/AVG_F223.png]

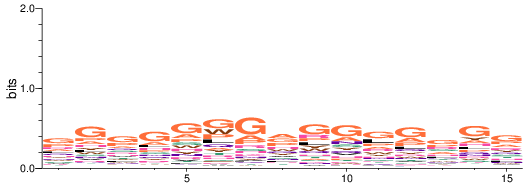

Supplement: S7 File — (ZIP) [file pcbi.1010238.s011.zip › disprot_html_table/logos/AVG_F224.png]

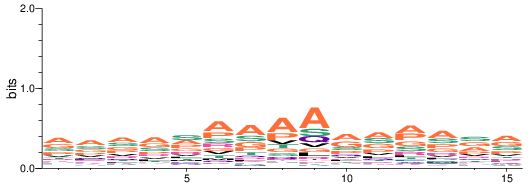

Supplement: S7 File — (ZIP) [file pcbi.1010238.s011.zip › disprot_html_table/logos/AVG_F225.png]

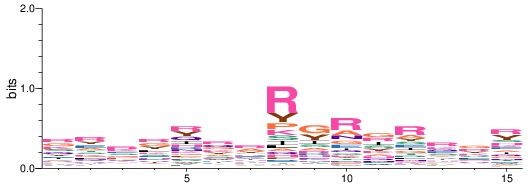

Supplement: S7 File — (ZIP) [file pcbi.1010238.s011.zip › disprot_html_table/logos/AVG_F226.png]

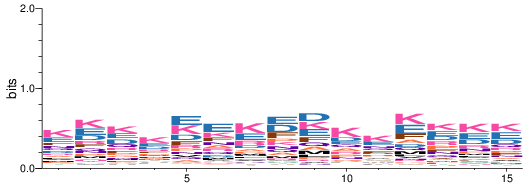

Supplement: S7 File — (ZIP) [file pcbi.1010238.s011.zip › disprot_html_table/logos/AVG_F227.png]

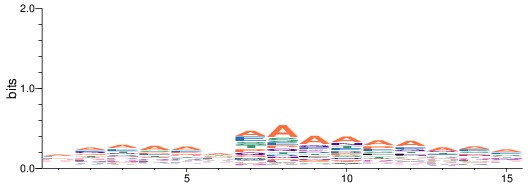

Supplement: S7 File — (ZIP) [file pcbi.1010238.s011.zip › disprot_html_table/logos/AVG_F228.png]

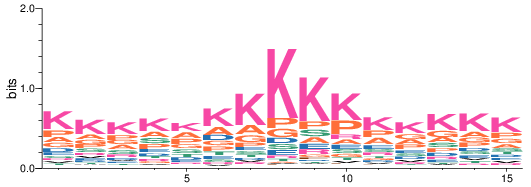

Supplement: S7 File — (ZIP) [file pcbi.1010238.s011.zip › disprot_html_table/logos/AVG_F229.png]

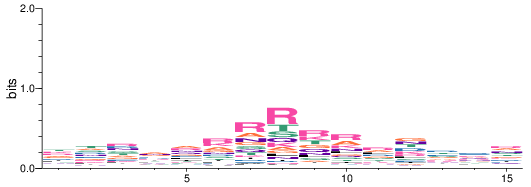

Supplement: S7 File — (ZIP) [file pcbi.1010238.s011.zip › disprot_html_table/logos/AVG_F23.png]

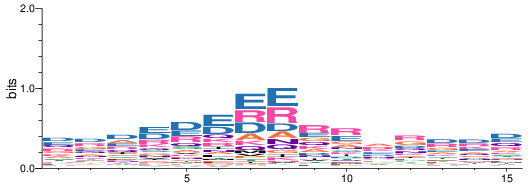

Supplement: S7 File — (ZIP) [file pcbi.1010238.s011.zip › disprot_html_table/logos/AVG_F230.png]

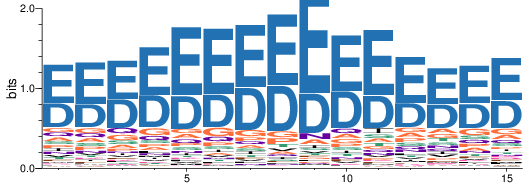

Supplement: S7 File — (ZIP) [file pcbi.1010238.s011.zip › disprot_html_table/logos/AVG_F231.png]

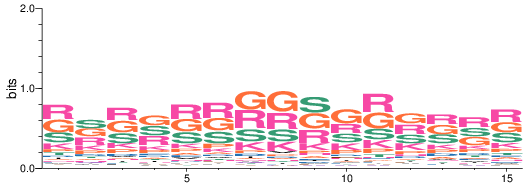

Supplement: S7 File — (ZIP) [file pcbi.1010238.s011.zip › disprot_html_table/logos/AVG_F232.png]

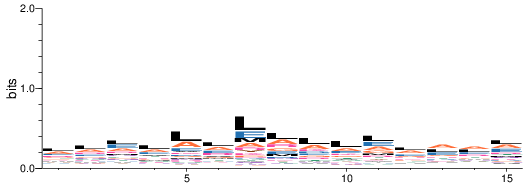

Supplement: S7 File — (ZIP) [file pcbi.1010238.s011.zip › disprot_html_table/logos/AVG_F233.png]

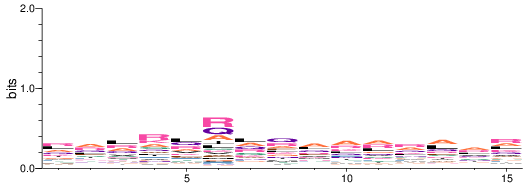

Supplement: S7 File — (ZIP) [file pcbi.1010238.s011.zip › disprot_html_table/logos/AVG_F234.png]

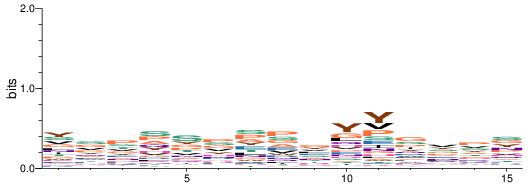

Supplement: S7 File — (ZIP) [file pcbi.1010238.s011.zip › disprot_html_table/logos/AVG_F235.png]

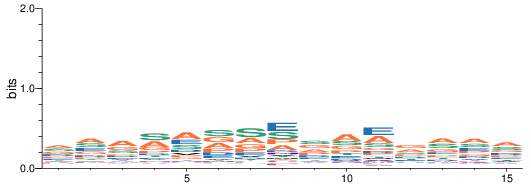

Supplement: S7 File — (ZIP) [file pcbi.1010238.s011.zip › disprot_html_table/logos/AVG_F236.png]

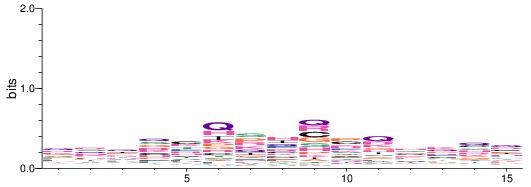

Supplement: S7 File — (ZIP) [file pcbi.1010238.s011.zip › disprot_html_table/logos/AVG_F237.png]

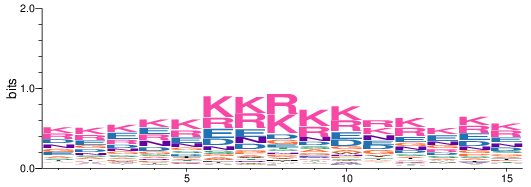

Supplement: S7 File — (ZIP) [file pcbi.1010238.s011.zip › disprot_html_table/logos/AVG_F238.png]

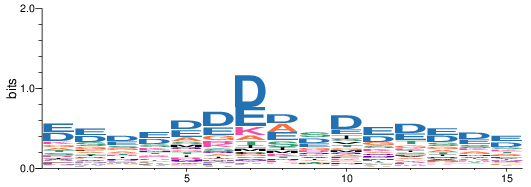

Supplement: S7 File — (ZIP) [file pcbi.1010238.s011.zip › disprot_html_table/logos/AVG_F239.png]

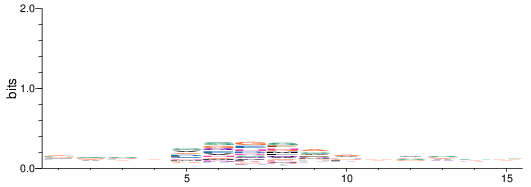

Supplement: S7 File — (ZIP) [file pcbi.1010238.s011.zip › disprot_html_table/logos/AVG_F24.png]

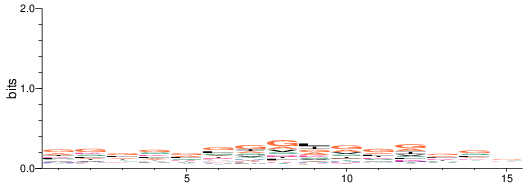

Supplement: S7 File — (ZIP) [file pcbi.1010238.s011.zip › disprot_html_table/logos/AVG_F240.png]

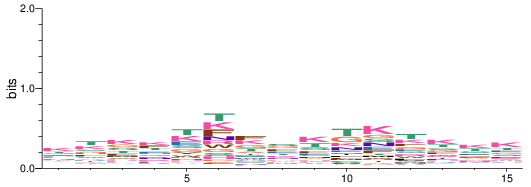

Supplement: S7 File — (ZIP) [file pcbi.1010238.s011.zip › disprot_html_table/logos/AVG_F241.png]

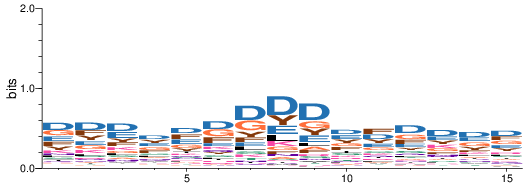

Supplement: S7 File — (ZIP) [file pcbi.1010238.s011.zip › disprot_html_table/logos/AVG_F242.png]

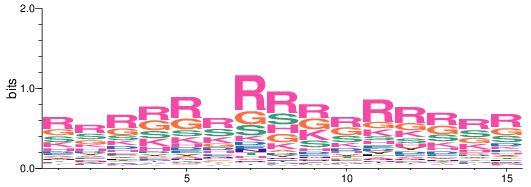

Supplement: S7 File — (ZIP) [file pcbi.1010238.s011.zip › disprot_html_table/logos/AVG_F243.png]

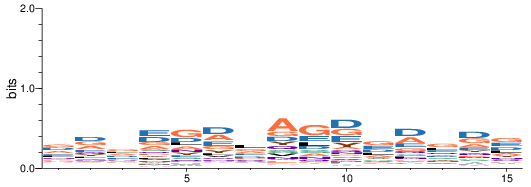

Supplement: S7 File — (ZIP) [file pcbi.1010238.s011.zip › disprot_html_table/logos/AVG_F244.png]

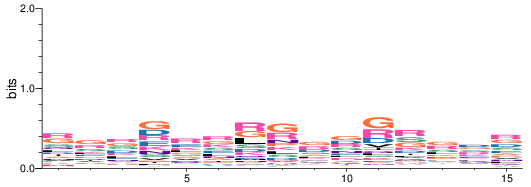

Supplement: S7 File — (ZIP) [file pcbi.1010238.s011.zip › disprot_html_table/logos/AVG_F245.png]

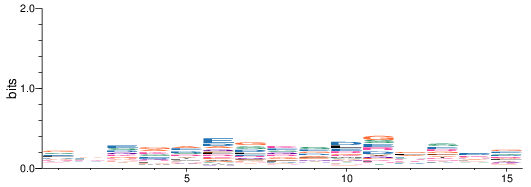

Supplement: S7 File — (ZIP) [file pcbi.1010238.s011.zip › disprot_html_table/logos/AVG_F246.png]

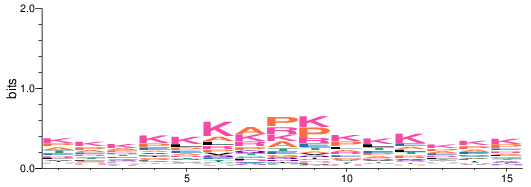

Supplement: S7 File — (ZIP) [file pcbi.1010238.s011.zip › disprot_html_table/logos/AVG_F247.png]

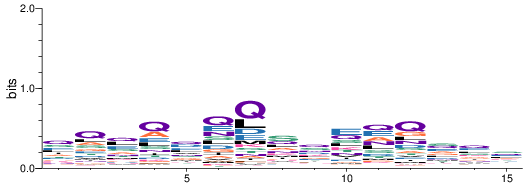

Supplement: S7 File — (ZIP) [file pcbi.1010238.s011.zip › disprot_html_table/logos/AVG_F248.png]

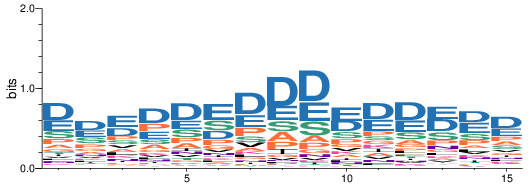

Supplement: S7 File — (ZIP) [file pcbi.1010238.s011.zip › disprot_html_table/logos/AVG_F249.png]

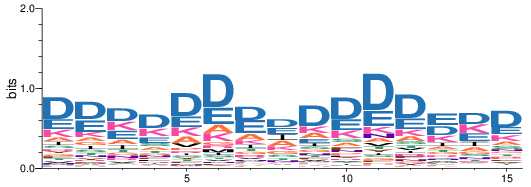

Supplement: S7 File — (ZIP) [file pcbi.1010238.s011.zip › disprot_html_table/logos/AVG_F25.png]

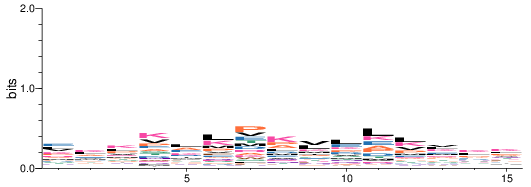

Supplement: S7 File — (ZIP) [file pcbi.1010238.s011.zip › disprot_html_table/logos/AVG_F250.png]

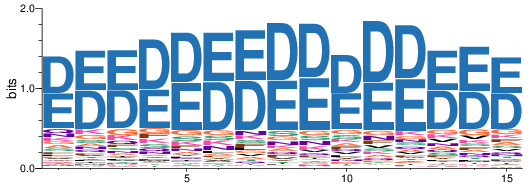

Supplement: S7 File — (ZIP) [file pcbi.1010238.s011.zip › disprot_html_table/logos/AVG_F251.png]

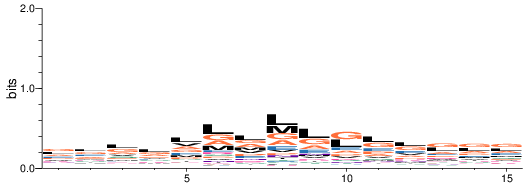

Supplement: S7 File — (ZIP) [file pcbi.1010238.s011.zip › disprot_html_table/logos/AVG_F252.png]

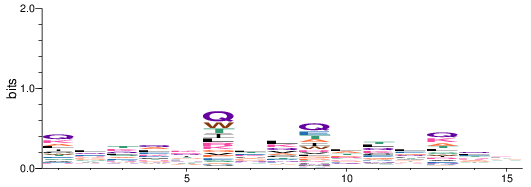

Supplement: S7 File — (ZIP) [file pcbi.1010238.s011.zip › disprot_html_table/logos/AVG_F253.png]

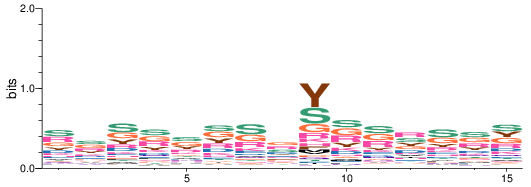

Supplement: S7 File — (ZIP) [file pcbi.1010238.s011.zip › disprot_html_table/logos/AVG_F254.png]

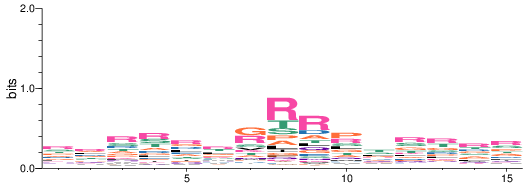

Supplement: S7 File — (ZIP) [file pcbi.1010238.s011.zip › disprot_html_table/logos/AVG_F255.png]

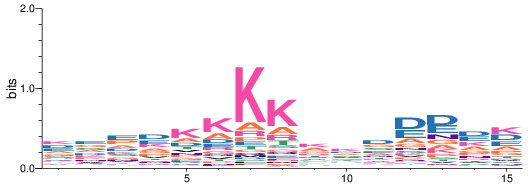

Supplement: S7 File — (ZIP) [file pcbi.1010238.s011.zip › disprot_html_table/logos/AVG_F26.png]

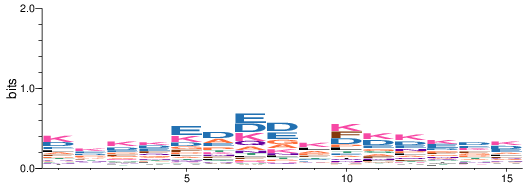

Supplement: S7 File — (ZIP) [file pcbi.1010238.s011.zip › disprot_html_table/logos/AVG_F27.png]

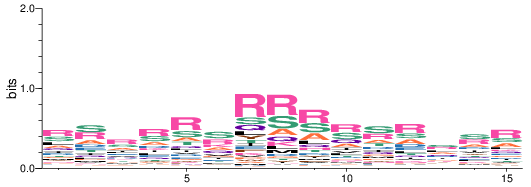

Supplement: S7 File — (ZIP) [file pcbi.1010238.s011.zip › disprot_html_table/logos/AVG_F28.png]

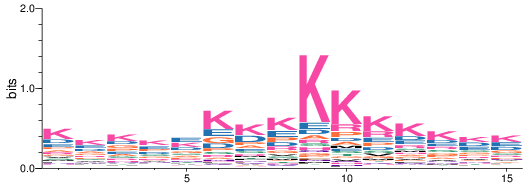

Supplement: S7 File — (ZIP) [file pcbi.1010238.s011.zip › disprot_html_table/logos/AVG_F29.png]

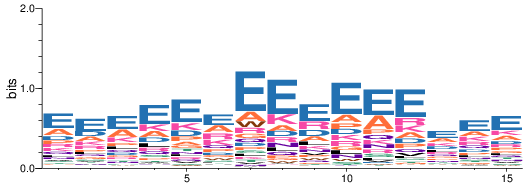

Supplement: S7 File — (ZIP) [file pcbi.1010238.s011.zip › disprot_html_table/logos/AVG_F3.png]

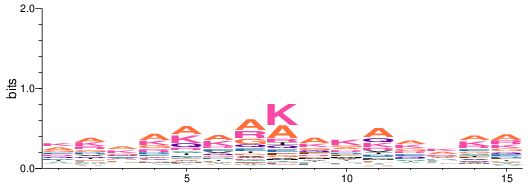

Supplement: S7 File — (ZIP) [file pcbi.1010238.s011.zip › disprot_html_table/logos/AVG_F30.png]

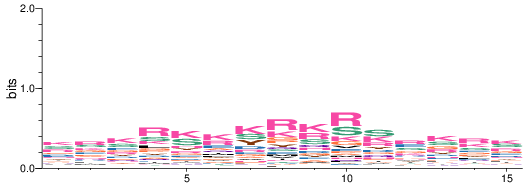

Supplement: S7 File — (ZIP) [file pcbi.1010238.s011.zip › disprot_html_table/logos/AVG_F31.png]

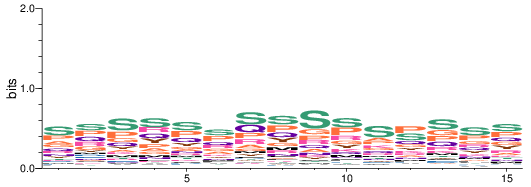

Supplement: S7 File — (ZIP) [file pcbi.1010238.s011.zip › disprot_html_table/logos/AVG_F32.png]

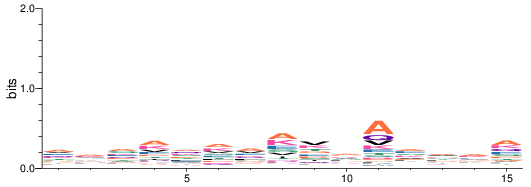

Supplement: S7 File — (ZIP) [file pcbi.1010238.s011.zip › disprot_html_table/logos/AVG_F33.png]

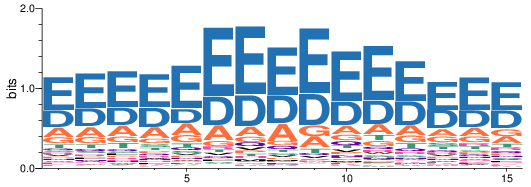

Supplement: S7 File — (ZIP) [file pcbi.1010238.s011.zip › disprot_html_table/logos/AVG_F34.png]

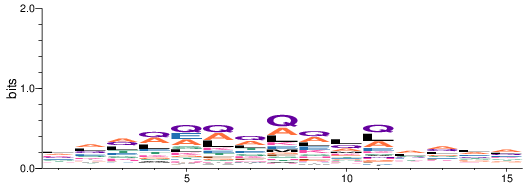

Supplement: S7 File — (ZIP) [file pcbi.1010238.s011.zip › disprot_html_table/logos/AVG_F35.png]

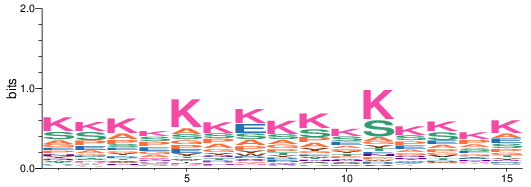

Supplement: S7 File — (ZIP) [file pcbi.1010238.s011.zip › disprot_html_table/logos/AVG_F36.png]

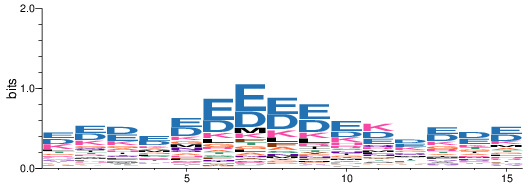

Supplement: S7 File — (ZIP) [file pcbi.1010238.s011.zip › disprot_html_table/logos/AVG_F37.png]

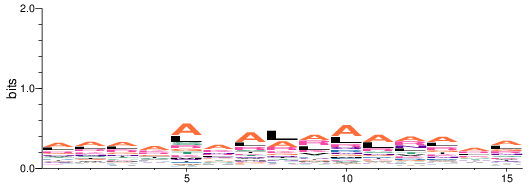

Supplement: S7 File — (ZIP) [file pcbi.1010238.s011.zip › disprot_html_table/logos/AVG_F38.png]

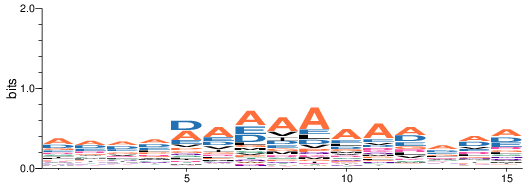

Supplement: S7 File — (ZIP) [file pcbi.1010238.s011.zip › disprot_html_table/logos/AVG_F39.png]

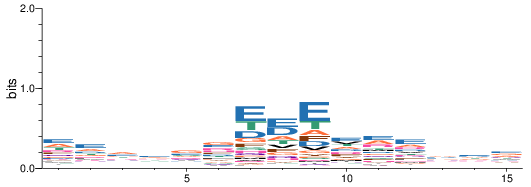

Supplement: S7 File — (ZIP) [file pcbi.1010238.s011.zip › disprot_html_table/logos/AVG_F4.png]

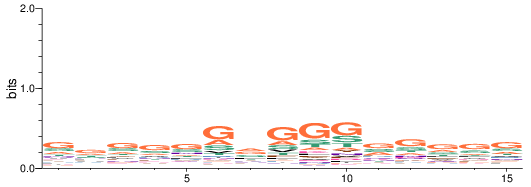

Supplement: S7 File — (ZIP) [file pcbi.1010238.s011.zip › disprot_html_table/logos/AVG_F40.png]

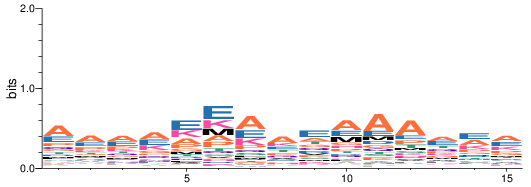

Supplement: S7 File — (ZIP) [file pcbi.1010238.s011.zip › disprot_html_table/logos/AVG_F41.png]

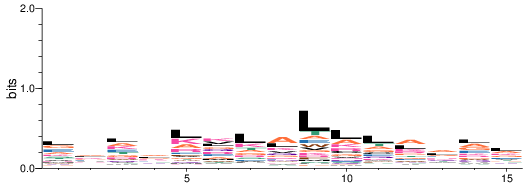

Supplement: S7 File — (ZIP) [file pcbi.1010238.s011.zip › disprot_html_table/logos/AVG_F42.png]

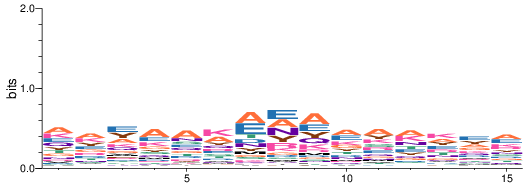

Supplement: S7 File — (ZIP) [file pcbi.1010238.s011.zip › disprot_html_table/logos/AVG_F43.png]

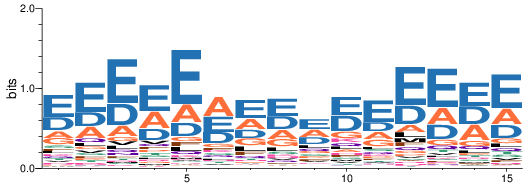

Supplement: S7 File — (ZIP) [file pcbi.1010238.s011.zip › disprot_html_table/logos/AVG_F44.png]
