# Supplementary material for: Discovering molecular features of intrinsically disordered regions by using evolution for contrastive learning
Source: PLoS Comput Biol. 2022 Jun 29;18(6):e1010238. doi: 10.1371/journal.pcbi.1010238 (PMC9275697; doi:10.1371/journal.pcbi.1010238)
Supplement: Attachment — Submitted filename: Lu_PLOS_COMP_BIO - clean copy.pdf [file pcbi.1010238.s014.pdf]

Summary

Lu et al. provide a well-written, timely, and informative method to quantify conserved sequence features via a novel machine learning approach. The scientific premise is sound, technical execution excellent, and the delivery in this manuscript a pleasure to read. I have some general comments which should be addressed prior to publication, but nothing that should impede acceptance. I congratulate the authors on yet another paper that sets the standard for applying complex statistical and machine-learning models to understand disordered protein sequences.

Major points:

- My biggest and most important suggestion is to *please* generate a set of tutorials for using and applying the code as performed in the results section. The full release of the code/data on GitHub is both essential and great, but specific, structured tutorials written in Markdown should be provided that would allow a (relative) novice to perform at least some of the analyses described in the paper. Specifically, there are two tutorials I like to see.
    - The first would be for the scenario in which someone has a set of homologous IDRs and wishes to train a new model on those IDRs. How does one go from a FASTA file of homologous IDRs to having a trained model that can score an arbitrary input IDR for homology to the training data? This should include instructions on how to select the randomly sampled IDRs.
    - The second would be the scenario in which I have a human or yeast IDR of interest and wish to ask how mutations to that IDR influence it's homology score (i.e. akin to the mutational scanning shown in figure 4B).
- I see these two tutorials (and others should the authors feel inclined) as sort of essential, in that as a methods paper making it as easy as possible for folks to use the method cleanly is critical. My hope is that this is not an overly burdensome request. The authors may wish to test-drive said tutorials using folks from outside the lab to ensure any kinks are worked out.

- Based on the methods, the authors state "*To standardize the lengths of the sequences, if the sequence was longer than 256 amino acids, we used the first and last 128 amino acids from the sequence*" - I must be misunderstanding this, because given many IDRs are longer than 256 residues this to me implies that residues outside the first/last 128 residues are discarded? This makes very little sense to me if true - why discard information in this manner? Some expansion on this, a justification for this approach if taken, and a comparative assessment of how the conclusions change if all IDRs > 256 residues are discarded seems like an important set of analyses to include? The authors justifying using CNNs, yet an LSTM-based NN would solve the variable length input problem (hence its application to biological sequence analysis). It is unclear what disadvantages an LSTM model brings to the table that would not outweigh the advantage of variable-length input. I obviously am not suggesting the authors re-do everything suing an LSTM-based NN, but, some discussion on the challenges associated with variable length sequences and a justification for the excision model I've

articulated above (if this is correct) would be important.

- A final more philosophical point. A key assumption that this method is based on is that homologous IDRs from orthologous proteins are under the same (or similar) selective pressures, such that even in the face of large-scale sequence divergence, an orthologous IDR from species A would be functionally equivalent in species B. This assumption is both reasonable and based on analogous inference made from decades of work where this assumption holds true for folded domains. That said, I think it is worth explicitly outlining this assumption - i.e. the idea that IDRs with conserved bulk properties are functionally conserved is (on a protein-by-protein basis) a hypothesis until proven otherwise. It's a very reasonable hypothesis, but I think one that should be explicitly detailed in the discussion.

Minor points:

- The authors may (and I mean may - if they do not feel it adds to the narrative absolutely no need to include) wish to include citations to a couple of additional papers that have examined sequence features as driving IDR function:
    - Schmidt, H. B., Barreau, A., & Rohatgi, R. (2019). Phase separation-deficient TDP43 remains functional in splicing. *Nature Communications*, *10*(1), 4890.
    - Dorone, Y., Boeynaems, S., Flores, E., Jin, B., Hateley, S., Bossi, F., Lazarus, E., Pennington, J. G., Michiels, E., De Decker, M., Vints, K., Baatsen, P., Bassel, G. W., Otegui, M. S., Holehouse, A. S., Exposito-Alonso, M., Sukenik, S., Gitler, A. D., & Rhee, S. Y. (2021). A prion-like protein regulator of seed germination undergoes hydration-dependent phase separation. *Cell*, *184*(16), 4284–4298.e27.
    - Boeynaems, S., Dorone, Y., Marian, A., Shabardina, V., Huang, G., Kim, G., Sanyal, A., Şen, N.-E., Docampo, R., Ruiz-Trillo, I., Lasker, K., Auburger, G., Kabashi, E., & Gitler, A. D. (2021). Poly(A)-binding protein is an ataxin-2 chaperone that emulsifies biomolecular condensates. In *bioRxiv* (p. 2021.08.23.457426). https://doi.org/10.1101/2021.08.23.457426
        - Bonus points if any of the insights described in the ataxin-2 paper can be explained using analysis here!

- The description and schematic depicted in Figure 1 is *extremely* helpful and very clearly articulates the overall approach. I love this, I wish more machine learning papers in biology provided such an accessible graphical overview of the method. This point has no rebuttal needed; I just really liked it and wanted to encourage this approach in future papers!

- "*as an example of a less characterized IDR*" - given the hnRNPA1 IDR has been assessed *in vitro* by NMR, EPR, SAXS, simulations and extensive studies of phase separation, has had evolutionary information, functional assessment, genetic disease

association examined, has had massive mutational analysis performed and use as now commonly used as a convenient IDR for synthetic systems, I would argue it is among the most well-characterized IDRs ever (Beijer et al., 2021; Bracha et al., 2018; Bremer et al., 2021; Dasmeh et al., 2021; Gui et al., 2019; Kim et al., 2013; Martin et al., 2020; Molliex et al., 2015; Ritsch, 2019; Sun et al., 2020).

- The authors should compare against recent work from the Wagner lab examining evolutionary constraints in phase separating proteins (notably for comparison with their hnRNPA1 work) (Dasmeh et al., 2021)

- "*While previous work did not analyze what amino acids are permissible as spacers between aromatic amino acids (Martin et al., 2020), we observe that in the wild-type sequence, the tyrosine residues are often flanked by serine or glycine (purple in Figure 7C)*" - more recent work has explicitly addressed this and it would be important (and interesting) to cross reference conclusions from that work(Bremer et al., 2021) against the conclusions drawn here.

- "Third, as an example of a less characterized IDR, we examined hnRNPA1, which has an Nterminal IDR from positions 183 to 372" - the IDR is C-terminal, not N-terminal!

- The authors raise "hydrophobic residues for activation domains" as being identified by Erijman et al, but the sequence feature of hydrophobic (and acidic) residues was first identified through systematic large-scale experiments by both Staller (Staller et al., 2018) and Ravarani (Ravarani et al., 2018) - a citation to these two papers would be appropriate here.
- The citation to the Zarin eLife paper appears twice (once as the preprint and once as the final article)

Beijer, D., Kim, H. J., Guo, L., O'Donovan, K., Mademan, I., Deconinck, T., Van Schil, K., Fare, C. M., Drake, L. E., Ford, A. F., Kochański, A., Kabzińska, D., Dubuisson, N., Van den Bergh, P., Voermans, N. C., Lemmers, R. J., van der Maarel, S. M., Bonner, D., Sampson, J. B., … Baets, J. (2021). Characterization of HNRNPA1 mutations defines diversity in pathogenic mechanisms and clinical presentation. *JCI Insight*, *6*(14). https://doi.org/10.1172/jci.insight.148363

Bracha, D., Walls, M. T., Wei, M.-T., Zhu, L., Kurian, M., Avalos, J. L., Toettcher, J. E., & Brangwynne, C. P. (2018). Mapping Local and Global Liquid Phase Behavior in Living Cells Using Photo-Oligomerizable Seeds. *Cell*, *175*(1-2), 1467–1480.e13.

Bremer, A., Farag, M., Borcherds, W. M., Peran, I., Martin, E. W., Pappu, R. V., & Mittag, T.

(2021). Deciphering how naturally occurring sequence features impact the phase behaviors of disordered prion-like domains. In *Cold Spring Harbor Laboratory* (p. 2021.01.01.425046). https://doi.org/10.1101/2021.01.01.425046

Dasmeh, P., Doronin, R., & Wagner, A. (2021). The length scale of multivalent interactions is evolutionarily conserved in fungal and vertebrate phase-separating proteins. In *bioRxiv* (p. 2021.05.04.442641). https://doi.org/10.1101/2021.05.04.442641

Gui, X., Luo, F., Li, Y., Zhou, H., Qin, Z., Liu, Z., Gu, J., Xie, M., Zhao, K., Dai, B., Shin, W. S., He, J., He, L., Jiang, L., Zhao, M., Sun, B., Li, X., Liu, C., & Li, D. (2019). Structural basis for reversible amyloids of hnRNPA1 elucidates their role in stress granule assembly. *Nature Communications*, *10*(1), 2006.

Kim, H. J., Kim, N. C., Wang, Y.-D., Scarborough, E. A., Moore, J., Diaz, Z., MacLea, K. S., Freibaum, B., Li, S., Molliex, A., Kanagaraj, A. P., Carter, R., Boylan, K. B., Wojtas, A. M., Rademakers, R., Pinkus, J. L., Greenberg, S. A., Trojanowski, J. Q., Traynor, B. J., … Taylor, J. P. (2013). Mutations in prion-like domains in hnRNPA2B1 and hnRNPA1 cause multisystem proteinopathy and ALS. *Nature*, *495*(7442), 467–473.

Martin, E. W., Holehouse, A. S., Peran, I., Farag, M., Incicco, J. J., Bremer, A., Grace, C. R., Soranno, A., Pappu, R. V., & Mittag, T. (2020). Valence and patterning of aromatic residues determine the phase behavior of prion-like domains. *Science*, *367*(6478), 694–699.

Molliex, A., Temirov, J., Lee, J., Coughlin, M., Kanagaraj, A. P., Kim, H. J., Mittag, T., & Taylor, J. P. (2015). Phase Separation by Low Complexity Domains Promotes Stress Granule Assembly and Drives Pathological Fibrillization. *Cell*, *163*(1), 123–133.

Ravarani, C. N. J., Erkina, T. Y., De Baets, G., Dudman, D. C., Erkine, A. M., & Madan Babu, M. (2018). High-throughput discovery of functional disordered regions: investigation of transactivation domains. *Molecular Systems Biology*, *14*(5), e8190.

Ritsch, I. (2019). *Distributions of molecular conformations and interactions revealed by EPR spectroscopy-methodology and application to hnRNPA1* [ETH Zurich].

https://www.research-collection.ethz.ch/handle/20.500.11850/417801

Staller, M. V., Holehouse, A. S., Swain-Lenz, D., Das, R. K., Pappu, R. V., & Cohen, B. A. (2018). A High-Throughput Mutational Scan of an Intrinsically Disordered Acidic Transcriptional Activation Domain. *Cell Systems*, *6*(4), 444–455.e6.

Sun, Y., Zhao, K., Xia, W., Feng, G., Gu, J., Ma, Y., Gui, X., Zhang, X., Fang, Y., Sun, B., Wang, R., Liu, C., & Li, D. (2020). The nuclear localization sequence mediates hnRNPA1 amyloid fibril formation revealed by cryoEM structure. *Nature Communications*, *11*(1), 6349.
