# Supplementary material for: Discovering molecular features of intrinsically disordered regions by using evolution for contrastive learning
Source: PLoS Comput Biol. 2022 Jun 29;18(6):e1010238. doi: 10.1371/journal.pcbi.1010238 (PMC9275697; doi:10.1371/journal.pcbi.1010238)
Supplement: Attachment — Submitted filename: reverse_homology_response2reviews.pdf [file pcbi.1010238.s015.pdf]

Department of Cell and Systems Biology
UNIVERSITY OF TORONTO · 25 HARBORD ST, TORONTO, CANADA M5S 3G5 · FAX (416) 978-8532

Dear Dr. Elofsson,

Thank you for considering our manuscript for publication in *PLOS Computational Biology*.

We were pleased that the reviewers were very interested in our new approach. However, the reviewers also had numerous concerns and suggestions that we have addressed in the revised manuscript. Specific highlights of the revisions include:

- New analysis of IDR features associated with microscopy image classes (Figure 5D,E).
- New motif-finding benchmarks (Figure 5A).
- Cross-validation experiments to assess generalization on the proxy task (Supplementary Figure 3).
- Improved description of the human IDR dataset (Methods).
- Improved web tutorials to allow others to access and train reverse homology models (See our github page at https://github.com/alexxijielu/reverse_homology/tree/main/tutorials)
- Added clearer discussion of the limitations of our approach and how it might be improved (Discussion).

We believe these and many other improvements (described below in point by point responses to the reviewers' specific comments) have greatly improved our manuscript so that it is more convincing and clear. We hope you will find it acceptable for publication in *PLoS Computational Biology*. We have highlighted the changes to the revised manuscript in yellow.

I would also like to thank you and the reviewers for your patience with the long delay in returning these revisions. As you will see from the affiliations, the lead and contributing authors have all moved on from my group, and it has been difficult to coordinate the revisions.

Sincerely,

Alan Moses, PhD
Professor and Canada Research Chair in Computational Biology
Departments of Cell & Systems Biology, and Computer Science
University of Toronto

Reviewer #1: The authors present a neural network based feature discovery method for IDRs, which they called "reverse homology" method. The model aims to exploits the evolutionary principle that functional features can be conserved in IDRs. Although the approach is very interesting, the presentation of the method is not clear and the results are not convincing enough.

We appreciate the reviewer's interest in the approach, and we have taken seriously the criticisms. We have attempted to clarify the presentation of the method and included

additional benchmarks to make the results more convincing. For example, we have added to the main figures the results of IDR function prediction benchmarks (Figure 3C) showing that the features selected by the classifier for prediction of mitochondrial targeting signals and Cdc28 substrate IDRs are readily interpretable (Figure 3D) and consistent with current understanding in these well-characterized systems. Although we emphasize that our approach is more general than motif-finding, we also now include a comparison with a state-of-the-art motif-finder on two benchmarks, and show that our global feature discovery approach yields qualitatively similar motifs (Figure 5A). Finally, we added a global analysis of features enriched in IDRs from human proteins with similar localization patterns (as assessed by unsupervised clustering of microscopy images from the human protein atlas) that reveals interesting differences in the molecular features of IDRs in the different subcellular localizations (Figure 5D,E)

Major:
Importantly, more details are needed to understand better how the reverse homology method works. For instance, it is unclear what the output of the neural network is. Also, more details about the measurement of the effect of mutations in the in-silico mutational scanning are necessary.

We have revised Figure 1 and the methods section describing the implementation details to make the method more clear. We have also clarified that output of the network is a classification of individual IDRs as “homologous” or “not” (with respect to an input set of 8 presumably homologous IDRs.) We modified Figure 1 to clarify the inputs (unaligned IDR sequences) and outputs (predictions of homology) of the network. Also, we have now clarified that the in-silico mutational scanning should be thought of as a visualization/exploration only at this stage. We clarify that have not demonstrated that the effects of the mutations in these plots are predictive of the functional effects of mutations in IDRs. Nevertheless, we believe the plots will be useful to researchers interested in generating hypotheses about the importance of residues in poorly characterized IDRs. We have clarified the details of this method and included tutorials on the github site.

Although the main purpose the method is not the detection of homologous sequences directly, it would be important the test the method's performance for the original task through cross-validation.

We thank the reviewer for this suggestion and we now include experiments that assess the performance on the proxy task on a held out validation set, which we include as supplementary figure 3. We find strong validation performance for both the yeast and human models.

The example for p27 nicely presents the capability of the method to retrieve functionally relevant features for human IDRs. However, the other examples, especially the identification of cysteine repeats in hair keratin-associated proteins

are less convincing. Presentation of other interesting human examples, like p27, could increase the biological value of this work.

We have replaced hair keratin with a poorly understood IDR from ataxin-2 (ATXN2) which was suggested by one of the reviewers (Figure 6C in the revised manuscript). We report that the neural network learns to recognize positively charged motifs and a proline-rich bulk property. The former is consistent with a recent study of this IDR, while the latter represents a new hypothesis for this IDR, which we believe is consistent with the in vivo proline-directed phosphorylation in this region.

We have also extended the discussion of the features identified in hnRNPA1 to include recently described preferences in the context of the aromatic residues (as suggested by one of the other reviewers) and some known disease mutations. As a more quantitative way to support our residue-level predictions, we also included motif-finding benchmarks of characterized human Grb2 (SH3) binding sites and yeast PKA phosphorylation sites, and found qualitatively similar motifs (but did not surpass the residue level recall of a state-of-the-art motif-finder, Figure 5A).

We acknowledge, however, that we did not demonstrate the power of the method to predict the effects of individual point mutations, and we have therefore referred to this in the revised manuscript as a visualization method.

Minor:
Small typos can be found in the manuscript:
Figure 1. legend: "D) This panel..."
Figure 6. legend: "For the heat maps..." (a bracket is absent)
Figure 6B.: Nop1 is indicated instead of Nop65
Position boundaries of Nop56 are not correct ("...K464 to D460 is the most important region...")

We have corrected these typos

Reviewer #2: Review uploaded as an attachment

Reviewer #3: The manuscript by Lu et al. describes a novel method to learn conserved sequence features in groups of homologous intrinsically disordered regions (IDRs) using unsupervised machine learning. This is potentially of interest, as more methods for the functional characterization and classification of IDRs are needed. The manuscript is unfortunately written in a style which tries to emphasize novelty by using a set of non-standard jargon terms and using many references to previous work which obfuscate rather than clarify its content. The text is also lengthy and dense, dwelling on relatively minor aspects while glossing over some key points which would help to evaluate its technical quality. It raises a number of major concerns:

Department of Cell and Systems Biology
UNIVERSITY OF TORONTO · 25 HARBORD ST, TORONTO, CANADA M5S 3G5 · FAX (416) 978-8532

*** Introduction. This presents a rather narrow view of the field, ignoring several key computational resources for IDRs and giving the impression that much work is still to do when it is really not. Specifically, the authors fail to mention and major database for IDRs, e.g. DisProt or MobiDB. These are important as a gold standard for IDRs (DisProt) and to facilitate large-scale analysis as well as functional annotation for IDR "flavors" (MobiDB). Instead, the authors largely limit their description of computational work to some self-references. This has to be corrected in order to appreciate the true novelty and impact of their contribution. Lastly, since the authors are proposing a neural network for IDRs, the recent CAID experiment is of relevance (Necci et al., Nature Methods 2021).**

We thank the reviewer for this suggestion and have added this important context to the introduction. We believe this will help clarify the distinction between our work and these major works in the field.

We have now added to the introduction:

Databases of large-scale predicted (Monzon et al. 2020) and experimentally confirmed (Hatos et al. 2020) intrinsically disordered regions are available. Prediction of intrinsically disordered regions from primary amino acid sequences is a well-developed area of research in computational biology (Necci et al. 2021). On the other hand, aside from defining a peptide region as intrinsically disordered, relatively little can be predicted about its function (Van Der Lee et al. 2014; Lindorff-Larsen and Kragelund 2021), although there has been considerable effort to identify binding sites (Jones and Cozzetto 2015; Mészáros, Erdős, and Dosztányi 2018), conditionally folded regions (Katuwawala et al. 2019) and more recently, to predict disordered region functions (Hu et al. 2021; Zarin et al. 2021). This is in contrast to the situation for folded protein regions, where highly specific predictions of function based on sequence using such universal resources as BLAST (Altschul et al. 1997)and Pfam(El-Gebali et al. 2019).

Other approaches have been devised to classify intrinsically disordered regions into functional groups, initially into a small number of groups based on predicted biophysical properties (Necci, Piovesan, and Tosatto 2016; R. K. Das, Ruff, and Pappu 2015). Using larger numbers of molecular features (including biophysical properties, matches to short motifs and repeats, and residue composition) and their patterns of evolution (Zarin et al. 2019) we showed strong association between ~20 biological functions and the molecular features of intrinsically disordered regions.

*** Methods.**

1. Terminology. While the use of "reverse homology" may overstate the novelty but can be justified, several other "inventions" do not: "letter map" has been called sequence logo or enrichment plot by the authors of the software used (LogoMaker); "in silico mutational scanning" really means a sequence permutation. These jargon terms should be replaced by standard ones to make the paper easier to understand.

In the revised manuscript, we have done our best to use terminology that is consistent with the literature.

We use the term “sequence logo” in our paper consistent with how it is used in the literature (and how it was proposed by Schneider et al. 1990) to display the information content in a column of a multiple alignment. Throughout our paper we used sequence logos to visually interpret the convolutional filters learned by our model. We have ensured in the revised version that this is consistent.

Alipanahi et al. 2015 used the term “mutation map” to describe the prediction of impacts of all single point mutations in a sequence, which they represented as a heatmap. Subsequently, this has been referred to in the literature as “in-silico mutagenesis” (examples include Koo & Eddy 2019) and this term is now in wide usage: for example, the paper entitled “In silico saturation mutagenesis of cancer genes” in *Nature* 2021.

In the revised manuscript, we have now reduced the use of this terminology and referred to the computational exploration of single amino acid residue substitutions as “mutation maps” consistent with the original Alipanahi et al. 2015 usage. We also use the term “letter map” to describe the visualization of the impact of single point changes at each position using letter sizes. These representations do not indicate information content (or any other measure of sequence variability) and therefore we believe it would be confusing (and inconsistent with the current usage) to refer to them as “sequence logos.” Nevertheless, we have reduced the use of “letter map” in the revised manuscript and made sure to clarify each terminology as it was introduced.

2. Implementation of reverse homology. This section is awkward to read. There is both a lengthy description of previous work, which does not belong in a materials and methods section, while several crucial elements are missing. If this reviewer understood correctly, the authors are proposing a plan convolutional neural network (CNN) trained to recognize IDRs. The details on this CNN should be spelled out clearly, e.g. using the recent DOME recommendations (Walsh et al., Nature Methods 2021). Last but not least, the sequence encoding hinted at in Figure 1D is not explained. Without further details it is impossible to evaluate the technical merit of this implementation.

We have clarified the description of the implementation details. We have included references to previous work because they guided the choices of many implementation details (such as architecture, query size, etc.). We have clearly stated that “The output of the final fully connected layer is considered the feature representation.”

We carefully reviewed the DOME specifications, and we made several revisions to ensure that all the applicable elements are found in the revised manuscript: we added the number of parameters, the run-time for generation of a mutation map, confidence

intervals for the evaluation metrics and more clearly explained the source and processing of the data.

Here is a summary of the DOME elements and where each can be found in our manuscript:
Data:
-Provenance: Explained in Methods
-Data splits: NOT APPLICABLE – UNSUPERVISED
-Redundancy between datasets: NOT APPLICABLE – UNSUPERVISED
-Availability of data: Uploaded on Zenodo

Optimization
-Algorithm: Adam, cited and parameters given.
-Meta-predictions: We rely on an IDR predictor, explained in methods.
-Data encoding: Explained in Methods sections on preprocessing and training
-Parameters: Explained in Methods (total number of parameters 1,479,384)
-Features: NOT APPLICABLE – raw sequences, not features were used
-Fitting: NOT APPLICABLE – model is being deployed unsupervised
-Regularization: No regularization to stop overfitting was used – see “Fitting”
-Availability of configuration: Yes – these are available in the Github repository

Model
-Interpretability: We describe our interpretability techniques under “Interpretation”
-Output: Described under “Implementation of reverse homology.”
-Execution time: we report that mutation maps for ATXN2 on our GPU take 209 seconds
-Availability of software: Github linked

Evaluation
-Evaluation method: Explained under benchmarks – multiple evaluations
-Performance measures: Explained in each section – enrichment, AUC, precision, etc.
-Comparison: other embedding methods, knowledge-based features, random features and sequence similarity
-Confidence: confidence intervals provided
-Availability of evaluation: Classification datasets provided in Zenodo

3. Training datasets. This section contains too many references to previous work, which hamper understanding and may obfuscate crucial details. E.g. what was the pairwise sequence identity within and between IDR groups? How do the authors treat "jumpy" alignments, with either lots of gaps or widely varying predicted IDRs across homologs? Is the presence of a bona fide IDR across homologs even checked? The choice of two different IDR predictors for the two datasets also needs an explanation (even though CAID, see above, may help).

Department of Cell and Systems Biology
UNIVERSITY OF TORONTO · 25 HARBORD ST, TORONTO, CANADA M5S 3G5 · FAX (416) 978-8532

We have clarified in the revised manuscript that no sequence alignments are used as input to the model. Homologous sets of IDRs are provided "unaligned". This is now clearly illustrated in the revised figure 1.

The set of IDRs used for the yeast analysis has been previously published and details about the sequence similarity, IDR predictions, etc., were provided in the original publication. We have now included additional details in our revised manuscript, but would like to avoid a full presentation of a previously published dataset.

We have now provided more details about the human dataset, which was prepared for this paper. We used SPOTdisorder because it was the best performing method in the evaluations at the time we prepared this dataset. In the revised manuscript we report the results of an all by all blastp search on the human reference IDRs, and as expected, more than 80% have no detectible homology to any other sequence in the set. During the course of our research, we did some experiments, and as expected, filtering out similar sequences makes little difference to the results of this approach. We note that because our proxy task is to tell apart homologous families of IDRs, sequence similarity between classes of IDRs will make the task harder, as the model will be forced to learn to distinguish between these similar sequences.

We used the OMA ortholog database because they were the easiest to download, and we did not check for the presence of IDRs across homologs. Although ideally we would rerun SPOTdisorder on all the species, in practice it is too slow. It is known that most IDRs are present in homologs over long evolutionary distances (Colak et al) and we find that because we are learning global features for the whole collection of IDRs, the approach is relatively insensitive to a small proportion of annotation errors/lineage-specific evolution.

Because the numbers of homologs vary considerably for each human protein in the OMA database, in the revised manuscript, we used a series of heuristics to filter the homologs to obtain a relatively similar total evolutionary distance in each homolog set, which was chosen to match the typical total evolutionary distances found in the yeast dataset. Although in practice, we found that this filtering step made little difference to the global features learned, we have included this filtering step in the revised manuscript to make the human dataset more uniform and comparable to the yeast dataset. We have now made the dataset public on zenodo, and described in detail the filtering heuristics applied in the Methods section. Although the heuristics do attempt to remove "redundant" homologs (sequences that are very similar to others in the homolog set) we do expect to include sequences with detectible sequence similarity (alignability) within these homolog sets (e.g., among orthologous mammalian IDRs). Although sequence similarity will tend to increase the accuracy on the proxy task, we do not expect it to generate much "learning signal" for the model: in contrastive learning, any feature representation will classify identical data points correctly (even randomly initialized features). This is different than the situation in supervised discriminative learning where identical sequences can simply be "memorized" by the model.

To verify this empirically, we have now performed some experiments on held-out validation data (Supplementary Figure 4). In these experiments, to rule out the effects of sequence similarity on the reported accuracy, we used blastp to remove any sequences from the validation sets that had detectible sequence similarity with sequences in the training set.

4. Preprocessing and training. The effect of cutting up the IDR to the first and last 128 residues has to be investigated. While the reasoning to avoid length differences is clear, the knock-on effects caused by this are not. This has to be spelled out clearly. What is being learned? Is the CNN "just another" IDR predictor for which single internal neurons are interpreted? This would be both novel (interpretation) and not so novel (predictor) at the same time.

We have reported the results of an experiment to demonstrate the effect of the choice of 256 residue windows. We find that the features learned by using different cropping heuristics are highly correlated. This analysis is reported in supplementary figure 3. We also note in the revised manuscript that the number of IDRs >256 residues comprise a small fraction of the dataset (6.2% for yeast and 13% for human) and this likely also explains why this makes relatively little impact on the global features.

We thank the reviewer for the interesting idea to interpret the neurons of an IDR predictor, which would reveal what features are important for telling apart ordered and disordered sequences. We believe this analysis is outside of the scope of this paper (which is already too long). We stress that this is *very* different than what we are doing here.

Once again, we emphasize that we are *not* learning a model to predict IDRs, and that this is a key misunderstanding of our contribution. Instead, we *start* with IDRs from a state-of-the-art predictor, and train a model to distinguish between 1000s of sets of homologous IDRs (where homology is established based on sequence similarity of folded regions and gene order conservation, but this information is not provided to the model). Because this model learns to tell one IDR from another, the features it learns must be related to the conserved functions of the IDRs. It is in this context that we wish to interpret these features.

5. Correlation with literature-curated features. How do the authors derive these? from single publications and only for the test cases described in the text? A more systematic approach is needed in order to have meaningful benchmarking, e.g. by comparing with DisProt annotations. This limitation makes Figure 3 unnecessarily difficult to evaluate and understand.

We have used a previously published set of 66 literature curated features. We have clarified in the revised manuscript that these include 37 ELM motifs, 7 physicochemical properties, 8 amino acid frequencies and 14 repeats. A complete list of these with

references is available as Table S1 from Zarin et al. 2019 and the correlations are reported in Supplementary File 3. The 37 ELM motifs in this set were chosen based on the number of characterized instances in the yeast proteome. To our knowledge, this is the largest available set of molecular features that have been described for IDRs.

The correlations are between the matches to literature curated features at each position in all the IDRs with the activations of the features discovered using reverse homology (described in the methods section “Correlation with literature-curated features”). We clarified this in the revised manuscript.

6. Benchmarking and comparison to other methods. This is completely glossed over. Why is there no comparison to other methods? This is easy enough to arrange and at the very least some baseline approaches are needed. E.g. how well does the method perform compared to BLAST or even simple pairwise sequence alignment? How does plain sequence conservation work on the IDRs? How well do motif-based methods work, e.g. ELM or Dilimot? The latter is for de novo motif identification, which is clearly what the authors are doing as well. It is surprising to see that no attempt was made to benchmark the method.

We strongly disagree with the reviewer’s comment that no attempt has been made to benchmark the method. We included three types of benchmarks: 1) We reported the correlation with a previously published set of 66 literature-curated features that includes 37 ELM motifs to show how well our method does at recovering these features. 2) We showed that the features learned by our method are comparable to literature-curated and deep-learning features for prediction of CDK phosphorylation sites mitochondrial targeting signals. 3) We performed a series of more general functional prediction tasks (based on GO annotations) against state-of-the-art protein feature learning methods. All of these benchmarks are included in Figure 3 and supplementary file 4, (tables 1 and 2), along with descriptions of the classification datasets in supplementary file 4.

Nevertheless, we have followed the reviewer’s suggestions as best we could in order to clarify and improve the benchmarking in the revised manuscript. We have:
1) Moved the CDK target and mitochonidral targeting signal analysis to Figure 3
2) Now included pairwise sequence identity from BLAST as a baseline for nearest neighbour benchmarks (as expected, the performance of sequence similarity on all of our IDR benchmarks is well below other methods)
3) Now included two motif-finding benchmarks against a state-of-the-art motif finder, DALEL. (Although in both cases we did find a feature that closely resembles the real motif as the most enriched, performance on the benchmarks does not surpass the state-of-the-art.)

7. Sequence features identified. The authors claim that their method learns "functional features", with "motifs" as well as "bulk properties". However, from the figures it is only possible to discern what look remarkably like sequence motifs, albeit not regular expressions.

Department of Cell and Systems Biology
UNIVERSITY OF TORONTO · 25 HARBORD ST, TORONTO, CANADA M5S 3G5 · FAX (416) 978-8532

We have clarified in the revised manuscript that the average pooling layer in our architecture allows the neural network to learn bulk properties that are distributed across the entire IDRs. We emphasize that the length of the sequence logos for these average pooled features is arbitrarily chosen for visualization purposes, but that the entire sequence is used when we compute them.

To further support this claim, we show that the features used for prediction of the yeast mitochondrial targeting signals are all “average pooled” features recognizing hydrophobic and positive charged residues. This is consistent with previous reports that these targeting signals contain these bulk features, but that they are distributed across the entire sequence.

In addition, we have now included a global analysis of features enriched in subcellular human proteins clustered based on their subcellular localization in microscopy images. We find that the various classes of membrane targetted/trafficked proteins show various types of bulk hydrophobic properties, while sub-compartments of the nucleus tend to show bulk charged features.

Finally, we have now added Supplementary File 1 comparing the average- and max-pooled top activating peptides for each feature. As expected they look very different.

8. In silico mutational studies. If this reviewer understood correctly, this somewhat hyperbolically named analysis presents different amino acid compibantions to the CNN and records the drop in scores. This is useful but should be described for what it is. It is NOT an experimental validation and can, in principle, be carried out on any neural network-based IDR predictor. The case for its usefulness should therefore be more clearly argued and/or the statements regarding it toned down (the latter applies widely throughout the text).

We have reduced the focus on the mutation maps in the revised manuscript, and clarified that they should be considered a visualization tool.

We take the reviewer’s point that this could be carried out with any neural network IDR predictor, but this is not what we have done here, and we believe it is beyond the scope of our work.

9. Biological examples. These are probably too much of a good thing and distract from the method. Rather than using a few case studies to argue in detail the nuances of their predictor, the authors should focus on validating the method at large. In light of this, the enthusiastic tone of the text should also be toned down.

We removed some of the specific biological examples and replaced them with statistical summaries and benchmarks in figures 3 and 5. We have reduced the optimistic tone throughout the manuscript.

Department of Cell and Systems Biology
UNIVERSITY OF TORONTO · 25 HARBORD ST, TORONTO, CANADA M5S 3G5 · FAX (416) 978-8532

10. Preprints. Several key statements in the manuscript are backed up by citations of preprints, including several self-citations. While this can sporadically be accepted, and indeed contribute to the "timeliness" of the paper, in its current form it is beyond the pale. This reviewer has counted a dozen preprints in the reference section. Checking these, in combination with the previously mentioned tendency to obfuscate the meaning with jargon and lack of key details, places an undue burden on unpaid reviewers trying to districate the true contribution of this manuscript.

We have tried to replace the preprints with their now published citations where possible. We note that some of these preprints were contributions to conferences that do not publish the proceedings or are not yet published. Therefore, the preprint version is what we have.

Citation of preprints is now common practice in bioinformatics and computational biology, and until some guidelines for this are developed, we are simply following the current standard in the field. While we take the reviewer's point that the citation of unpublished (and therefore not adequately peer-reviewed) scientific work is problematic, we believe this is a discussion that is beyond the scope of the current reviews.

11. Discussion. Statements referring to "excitement" and "urgent needs" need to be toned down in accordance with the previous remarks. Also, the authors have built a predictor and not made a "comparative proteomics" study.

We believe this comment (and the overall lack of enthusiasm for our work) stems from the reviewer's misunderstanding that we have developed an IDR predictor.

To clarify once again why work is very exciting (for computational biology in general, even beyond IDRs): To our knowledge, it is the first time a deep-learning approach has been designed to learn features of biological sequences that are conserved over evolution. We have added a clear statement to this effect at the end of the introduction.

Nevertheless, we have reduced the optimistic tone throughout the manuscript.

Specific points

a. Introduction. Tools and databases are not cited, e.g. BLAST and Pfam.

We have corrected these omissions.

b. Sequence "homology". This should be referred to as either "homology" or "sequence similaritty/identity". Homology is not a quantitative term.

We thank the reviewer for pointing out this mistake. We have corrected this error.

Department of Cell and Systems Biology
UNIVERSITY OF TORONTO · 25 HARBORD ST, TORONTO, CANADA M5S 3G5 · FAX (416) 978-8532

Have the authors made all data and (if applicable) computational code underlying the findings in their manuscript fully available?
The PLOS Data policy requires authors to make all data and code underlying the findings described in their manuscript fully available without restriction, with rare exception (please refer to the Data Availability Statement in the manuscript PDF file). The data and code should be provided as part of the manuscript or its supporting information, or deposited to a public repository. For example, in addition to summary statistics, the data points behind means, medians and variance measures should be available. If there are restrictions on publicly sharing data or code —e.g. participant privacy or use of data from a third party— those must be specified.

Reviewer #1: None
Reviewer #2: Yes
Reviewer #3: No: The authors state that "all data will be made available at the author website".

PLOS authors have the option to publish the peer review history of their article (what does this mean?). If published, this will include your full peer review and any attached files.

If you choose "no", your identity will remain anonymous but your review may still be made public.

Do you want your identity to be public for this peer review? For information about this choice, including consent withdrawal, please see our Privacy Policy.

Reviewer #1: No
Reviewer #2: No
Reviewer #3: No

Summary Lu et al. provide a well-written, timely, and informative method to quantify conserved sequence features via a novel machine learning approach. The scientific premise is sound, technical execution excellent, and the delivery in this manuscript a pleasure to read. I have some general comments which should be addressed prior to publication, but nothing that should impede acceptance. I congratulate the authors on yet another paper that sets the standard for applying complex statistical and machine-learning models to understand disordered protein sequences.
Major points:
● My biggest and most important suggestion is to please generate a set of tutorials for using and applying the code as performed in the results section. The

full release of the code/data on GitHub is both essential and great, but specific, structured tutorials written in Markdown should be provided that would allow a (relative) novice to perform at least some of the analyses described in the paper. Specifically, there are two tutorials I like to see.
○ The first would be for the scenario in which someone has a set of homologous IDRs and wishes to train a new model on those IDRs. How does one go from a FASTA file of homologous IDRs to having a trained model that can score an arbitrary input IDR for homology to the training data? This should include instructions on how to select the randomly sampled IDRs.
○ The second would be the scenario in which I have a human or yeast IDR of interest and wish to ask how mutations to that IDR influence it's homology score (i.e. akin to the mutational scanning shown in figure 4B).

● I see these two tutorials (and others should the authors feel inclined) as sort of essential, in that as a methods paper making it as easy as possible for folks to use the method cleanly is critical. My hope is that this is not an overly burdensome request. The authors may wish to test-drive said tutorials using folks from outside the lab to ensure any kinks are worked out.

We thank the reviewer for these suggestions. We have now done our best to provide "easy to follow" instructions to train models and visualize the key residues in IDRs. These are available at our github site:
https://github.com/alexxijielu/reverse_homology/tree/main/tutorials

We note, however, that we do not believe the current implementation/analysis represents the "final" version of IDR analysis using "reverse homology" or contrastive learning more generally. This paper/approach is the result of more than 3 years of experimentation and refinement. For practical reasons (personnel moving on), we decided it was time for us wrap up this first phase of the project. We hope that intrepid IDR bioinformaticians will continue to improve on these approaches. We have included these sentiments as a preamble to the tutorial provided. To clarify this limitation, we have avoided referring to our work as a "method" in the revised manuscript.

● Based on the methods, the authors state "To standardize the lengths of the sequences, if the sequence was longer than 256 amino acids, we used the first and last 128 amino acids from the sequence" - I must be misunderstanding this, because given many IDRs are longer than 256 residues this to me implies that residues outside the first/last 128 residues are discarded? This makes very little sense to me if true - why discard information in this manner? Some expansion on this, a justification for this approach if taken, and a comparative assessment of how the conclusions change if all IDRs > 256 residues are discarded seems like an important set of analyses to include? The authors justifying using CNNs, yet an LSTM-based NN would solve the variable length input problem (hence its application to biological sequence analysis). It is unclear what disadvantages an LSTM model brings to the table that would not outweigh the advantage of variable-length input. I obviously am not suggesting the authors re-do everything

Department of Cell and Systems Biology
UNIVERSITY OF TORONTO · 25 HARBORD ST, TORONTO, CANADA M5S 3G5 · FAX (416) 978-8532

suing an LSTM-based NN, but, some discussion on the challenges associated with variable length sequences and a justification for the excision model I've articulated above (if this is correct) would be important.

The reviewer is right that future versions of reverse homology will use transformers or other state-of-the-art variable length sequence encoders. When we started this project, unfortunately, transformers were not widely implemented and interpretation techniques for them were not widely available. Again, we hope that this is the first paper about "reverse homology" or similar evolutionarily inspired deep learning methods, not the last.

We do not consider LSTMs here because the features they learn are generally not interpretable, and our goal was to identify features of IDRs that we could interpret and visualize. We have clarified our focus on interpretability and visualization in the revised manuscript.

We have included some experiments to test the effect of clipping the IDRs to 256 residues (Supplementary figure 3). We find that the majority of features the model learns using the 128 residues at either ends of the IDR (for IDRs with length >256) are highly correlated with features the model learns when using the 256 central residues (for IDRs with length >256). Because we are learning features as a proteome-wide scale, it's not critical that we actually include all of the sequence from each IDR. If we miss some of the features in one IDR, we believe we will pick them up in another. We also note in the revised manuscript that the IDRs >256 residues comprise a small fraction of the dataset (6.2% for yeast and 13% for human) and this likely also explains why this makes relatively little impact on the global features.

Practically, increasing the length of the sequence doesn't help the learning much because the neural network can already mostly solve the proxy task with 256 residues (supplementary figure 4). So there isn't much additional learning signal provided if we make the sequences longer. Once again, we acknowledge that this is a limitation of our approach, and in this sense the masked token prediction used to train language models is superior. However, the benefits of language models come at a high computational cost and challenges with interpretability. We are confident that these will be addressed in future work, but we believe it is outside of the scope of our current paper.

- **A final more philosophical point. A key assumption that this method is based on is that homologous IDRs from orthologous proteins are under the same (or similar) selective pressures, such that even in the face of large-scale sequence divergence, an orthologous IDR from species A would be functionally equivalent in species B. This assumption is both reasonable and based on analogous inference made from decades of work where this assumption holds true for folded domains. That said, I think it is worth explicitly outlining this assumption - i.e. the idea that IDRs with conserved bulk properties are functionally conserved**

is (on a protein-by-protein basis) a hypothesis until proven otherwise. It's a very reasonable hypothesis, but I think one that should be explicitly detailed in the discussion.

This is an important point, as this is the assumption that underlies all comparative genomics approaches. We have added this to the Discussion in the revised manuscript.

Minor points:

- **The authors may (and I mean may - if they do not feel it adds to the narrative absolutely no need to include) wish to include citations to a couple of additional papers that have examined sequence features as driving IDR function:**
  - **Schmidt, H. B., Barreau, A., & Rohatgi, R. (2019). Phase separation-deficient TDP43 remains functional in splicing. Nature Communications, 10(1), 4890.**
  - **Dorone, Y., Boeynaems, S., Flores, E., Jin, B., Hateley, S., Bossi, F., Lazarus, E., Pennington, J. G., Michiels, E., De Decker, M., Vints, K., Baatsen, P., Bassel, G. W., Otegui, M. S., Holehouse, A. S., Exposito-Alonso, M., Sukenik, S., Gitler, A. D., & Rhee, S. Y. (2021). A prion-like protein regulator of seed germination undergoes hydration-dependent phase separation. Cell, 184(16), 4284–4298.e27.**
  - **Boeynaems, S., Dorone, Y., Marian, A., Shabardina, V., Huang, G., Kim, G., Sanyal, A., Şen, N.-E., Docampo, R., Ruiz-Trillo, I., Lasker, K., Auburger, G., Kabashi, E., & Gitler, A. D. (2021). Poly(A)-binding protein is an ataxin-2 chaperone that emulsifies biomolecular condensates. In bioRxiv (p. 2021.08.23.457426). https://doi.org/10.1101/2021.08.23.457426**

■ Bonus points if any of the insights described in the ataxin-2 paper can be explained using analysis here!

We have now included IDR2 from ataxin-2 (ATXN2) as an example in Figure 6C (replacing hair keratin). We report that the neural network learns to recognize positively charged motifs and a proline-rich bulk property. The former is consistent with the findings of Boeynaems et al, while the latter represents a new hypothesis for this IDR, which we believe is consistent with the in vivo proline-directed phosphorylation in this region.

- **The description and schematic depicted in Figure 1 is extremely helpful and very clearly articulates the overall approach. I love this, I wish more machine learning papers in biology provided such an accessible graphical overview of the method. This point has no rebuttal needed; I just really liked it and wanted to encourage this approach in future papers!**

We thank the reviewer for the supportive comment.

- **"as an example of a less characterized IDR" - given the hnRNPA1 IDR has been assessed in vitro by NMR, EPR, SAXS, simulations and extensive studies of phase separation, has had evolutionary information, functional assessment,**

Department of Cell and Systems Biology
UNIVERSITY OF TORONTO · 25 HARBORD ST, TORONTO, CANADA M5S 3G5 · FAX (416) 978-8532

genetic disease association examined, has had massive mutational analysis performed and use as now commonly used as a convenient IDR for synthetic systems, I would argue it is among the most well-characterized IDRs ever (Beijer et al., 2021; Bracha et al., 2018; Bremer et al., 2021; Dasmeh et al., 2021; Gui et al., 2019; Kim et al., 2013; Martin et al., 2020; Molliex et al., 2015; Ritsch, 2019; Sun et al., 2020).

We have changed the description of hnRNAPA1 and added discussion of Bremer et al's work (see below). We have now added ataxin-2 as an example of a less characterized IDR.

- **The authors should compare against recent work from the Wagner lab examining evolutionary constraints in phase separating proteins (notably for comparison with their hnRNPA1 work) (Dasmeh et al., 2021)**

We have added a references to the work from Dasmeh & Wagner. However, we did not find their hnRNPA1 work (the reference given, which is the paper we know, concerns the FET family and yeast Dcp2)

- **"While previous work did not analyze what amino acids are permissible as spacers between aromatic amino acids (Martin et al., 2020), we observe that in the wild-type sequence, the tyrosine residues are often flanked by serine or glycine (purple in Figure 7C)" - more recent work has explicitly addressed this and it would be important (and interesting) to cross reference conclusions from that work(Bremer et al., 2021) against the conclusions drawn here.**

We thank the reviewer for pointing out the analysis of Bremer et al, of which we were not aware. That work did a large-scale evolutionary analysis of hnRNPA2 and found that the composition of homologous IDRs was generally conserved and suggested several interesting insights. 1) R is favoured over K, 2) Y and F are negatively correlated, suggesting that they perform similar functions, but Y is much more commonly observed and is a stronger "sticker" in experiments 3) that the charge of the region is constrained to be very slightly positive. Remarkably, some of these observations are born out in the "letter map" for average feature 32. R does appear to be favoured, while K is not. Also, negatively charged residues appear disfavoured, consistent with the idea that they destabilize the important interactions. Finally, the preference for G and S matches with the idea that these represent the "spacers". We note also, that not all Gs and Ss are equally preferred by the neural network feature, which seems to specifically favour Gs and Ss adjacent to the aromatic residues. We also note that, intriguingly, some ALS-causing mutations fall immediately adjacent to aromatic residues in this region. We have now discussed this briefly in the revised manuscript.

- **"Third, as an example of a less characterized IDR, we examined hnRNPA1, which has an Nterminal IDR from positions 183 to 372" - the IDR is C-terminal, not N-terminal!**

We have corrected this mistake.

● The authors raise "hydrophobic residues for activation domains" as being identified by Erijman et al, but the sequence feature of hydrophobic (and acidic) residues was first identified through systematic large-scale experiments by both Staller (Staller et al., 2018) and Ravarani (Ravarani et al., 2018) - a citation to these two papers would be appropriate here.

We have added these references and remove the attribution of discovery

● The citation to the Zarin eLife paper appears twice (once as the preprint and once as the final article)

We have corrected this mistake

Beijer, D., Kim, H. J., Guo, L., O'Donovan, K., Mademan, I., Deconinck, T., Van Schil, K., Fare, C. M., Drake, L. E., Ford, A. F., Kochański, A., Kabzińska, D., Dubuisson, N., Van den Bergh, P., Voermans, N. C., Lemmers, R. J., van der Maarel, S. M., Bonner, D., Sampson, J. B., … Baets, J. (2021). Characterization of HNRNPA1 mutations defines diversity in pathogenic mechanisms and clinical presentation. JCI Insight, 6(14). https://doi.org/10.1172/jci.insight.148363 Bracha, D., Walls, M. T., Wei, M.-T., Zhu, L.,

Kurian, M., Avalos, J. L., Toettcher, J. E., & Brangwynne, C. P. (2018). Mapping Local and Global Liquid Phase Behavior in Living Cells Using Photo-Oligomerizable Seeds. Cell, 175(1-2), 1467–1480.e13. Bremer, A., Farag, M., Borcherds, W. M., Peran, I., Martin, E. W.,

Pappu, R. V., & Mittag, T. (2021). Deciphering how naturally occurring sequence features impact the phase behaviors of disordered prion-like domains. In Cold Spring Harbor Laboratory (p. 2021.01.01.425046). https://doi.org/10.1101/2021.01.01.425046 Dasmeh, P.,

Doronin, R., & Wagner, A. (2021). The length scale of multivalent interactions is evolutionarily conserved in fungal and vertebrate phase-separating proteins. In bioRxiv (p. 2021.05.04.442641). https://doi.org/10.1101/2021.05.04.442641

Gui, X., Luo, F., Li, Y., Zhou, H., Qin, Z., Liu, Z., Gu, J., Xie, M., Zhao, K., Dai, B., Shin, W. S., He, J., He, L., Jiang, L., Zhao, M., Sun, B., Li, X., Liu, C., & Li, D. (2019). Structural basis for reversible amyloids of hnRNPA1 elucidates their role in stress granule assembly. Nature Communications, 10(1), 2006.

Kim, H. J., Kim, N. C., Wang, Y.-D., Scarborough, E. A., Moore, J., Diaz, Z., MacLea, K. S., Freibaum, B., Li, S., Molliex, A., Kanagaraj, A. P., Carter, R., Boylan, K. B., Wojtas, A. M., Rademakers, R., Pinkus, J. L., Greenberg, S. A., Trojanowski,

J. Q., Traynor, B. J., … Taylor, J. P. (2013). Mutations in prion-like domains in hnRNPA2B1 and hnRNPA1 cause multisystem proteinopathy and ALS. Nature, 495(7442), 467–473.

Martin, E. W., Holehouse, A. S., Peran, I., Farag, M., Incicco, J. J., Bremer, A., Grace, C. R., Soranno, A., Pappu, R. V., & Mittag, T. (2020). Valence and patterning of aromatic residues determine the phase behavior of prion-like domains. Science, 367(6478), 694–699.

Molliex, A., Temirov, J., Lee, J., Coughlin, M., Kanagaraj, A. P., Kim, H. J., Mittag, T., & Taylor, J. P. (2015). Phase Separation by Low Complexity Domains Promotes Stress Granule Assembly and Drives Pathological Fibrillization. Cell, 163(1), 123–133.

Ravarani, C. N. J., Erkina, T. Y., De Baets, G., Dudman, D. C., Erkine, A. M., & Madan Babu, M. (2018). High-throughput discovery of functional disordered regions: investigation of transactivation domains. Molecular Systems Biology, 14(5), e8190. Ritsch, I. (2019). Distributions of molecular conformations and interactions revealed by EPR spectroscopy-methodology and application to hnRNPA1 [ETH Zurich]. https://www.research-collection.ethz.ch/handle/20.500.11850/417801 Staller, M. V.,

Holehouse, A. S., Swain-Lenz, D., Das, R. K., Pappu, R. V., & Cohen, B. A. (2018). A High-Throughput Mutational Scan of an Intrinsically Disordered Acidic Transcriptional Activation Domain. Cell Systems, 6(4), 444–455.e6.

Sun, Y., Zhao, K., Xia, W., Feng, G., Gu, J., Ma, Y., Gui, X., Zhang, X., Fang, Y., Sun, B., Wang, R., Liu, C., & Li, D. (2020). The nuclear localization sequence mediates hnRNPA1 amyloid fibril formation revealed by cryoEM structure. Nature Communications, 11(1), 6349.
