# Supplementary material for: Discovering molecular features of intrinsically disordered regions by using evolution for contrastive learning
Source: PLoS Comput Biol. 2022 Jun 29;18(6):e1010238. doi: 10.1371/journal.pcbi.1010238 (PMC9275697; doi:10.1371/journal.pcbi.1010238)
Supplement: Attachment — Submitted filename: reverse_homology_response2reviews2.pdf [file pcbi.1010238.s016.pdf]

Department of Cell and Systems Biology

UNIVERSITY OF TORONTO · 25 HARBORD ST, TORONTO, CANADA M5S 3G5 · FAX (416) 978-8532

Reviewer #1: The Authors have done significant improvements in the revised manuscript that have made the study more convincing. They have revised the explanation of how their neural network based method works, which is one of the most important parts of the work. Taken together, the improved version is clear, the results are easier to interpret now.

Minor points:
• The method should be also tested on experimentally verified disordered regions (e.g. Disprot database)

We thank the reviewer from this interesting suggestion and have now used reverse homology to learn molecular features for experimentally verified IDRs from the DisProt database. We found that even with this smaller dataset, we were able to discover interesting and diverse features, many of which appear similar to those identified in the proteome-wide analyses. Further, we show that the features learned from DisProt perform relatively well at classifying human IDR homolog sets predicted by SPOT-Disorder, and are correlated with literature curated features. We have added these results to our manuscript (Supplementary Files 7 and 8 and Supplementary Figure 4C)

• Small typos:
"Overall, we find that while some GO Slim categories are highly enriched with both feature sets
(e.g. "Cell wall organization", "Translation" or "Ribosome biogenesis", highlighted in Figure
3A)…" - figure reference is not correct.

We have corrected this error

"Examination of the of the most activating"

We have corrected this error

Reviewer #2: The authors have done an excellent job addressing the reviewer comments (some of which I felt were somewhat counterproductive). I have no further comments and strongly support publication.

Reviewer #3: The authors have significantly improved the manuscript after revision. All concerns have been answered extensively and satisfactorily. Good job.
