# Supplementary material for: Discovering molecular features of intrinsically disordered regions by using evolution for contrastive learning
Source: PLoS Comput Biol. 2022 Jun 29;18(6):e1010238. doi: 10.1371/journal.pcbi.1010238 (PMC9275697; doi:10.1371/journal.pcbi.1010238)
Supplement: S2 File — (TAR.GZ) [file pcbi.1010238.s006.tar.gz › s2/yeast_table.html]

yeast table annotated


|  |  |  |  |  |  |  |
| --- | --- | --- | --- | --- | --- | --- |
| **Feature** | **Max Logo** | **Max Enrichments** | **Max Annotation** | **Average Logo** | **Average Enrichments** | **Average Annotation** |
| **Feature 42** |  |  |  |  |  |  |
| **Feature 77** |  |  |  |  |  |  |
| **Feature 40** |  | nuclear protein-containing complex; 10/73; 6.5808E-04 |  |  |  |  |
| **Feature 255** |  |  |  |  | cytoplasm; 5/19; 5.7485E-03 |  |
| **Feature 43** |  |  |  |  |  | A-repeats |
| **Feature 230** |  |  |  |  | RNA binding; 3/20; 9.2650E-03 |  |
| **Feature 90** |  | carbohydrate:cation symporter activity; 3/206; 3.4525E-03  carbohydrate transmembrane transporter activity; 3/206; 3.4525E-03  carbohydrate:proton symporter activity; 3/206; 3.4525E-03  DNA repair; 5/206; 7.7677E-03 |  |  | intrinsic component of membrane; 11/135; 3.2713E-03  integral component of membrane; 10/135; 6.0516E-03 |  |
| **Feature 33** |  | proteolysis; 5/86; 3.7203E-03 |  |  | alpha-amino acid metabolic process; 4/82; 7.5252E-05  acyltransferase activity; 4/82; 2.7647E-04  small molecule catabolic process; 3/82; 4.4651E-04  carboxylic acid catabolic process; 3/82; 4.4651E-04 |  |
| **Feature 109** |  | kinase activity; 13/297; 4.9203E-04  transferase activity; 23/297; 6.7912E-04  transferase activity, transferring phosphorus-containing groups; 15/297; 8.2898E-04  protein serine/threonine kinase activity; 9/297; 8.7834E-04 |  |  |  |  |
| **Feature 39** |  | regulation of biological process; 35/329; 8.3606E-04  regulation of cellular process; 32/329; 1.3660E-03  RNA biosynthetic process; 7/329; 1.9081E-03  cellular component organization or biogenesis; 32/329; 2.4575E-03 |  |  | plasma membrane; 4/19; 3.7785E-04  cellular\_component; 15/19; 4.4330E-03 |  |
| **Feature 41** |  | supramolecular complex; 5/161; 1.0779E-03  cytoplasmic ribonucleoprotein granule; 3/161; 5.4085E-03  ribonucleoprotein granule; 3/161; 5.4085E-03  site of polarized growth; 4/161; 6.5524E-03 |  |  |  |  |
| **Feature 91** |  | dephosphorylation; 3/103; 8.7650E-04  negative regulation of cellular macromolecule biosynthetic process; 4/103; 2.1855E-03  negative regulation of macromolecule biosynthetic process; 4/103; 2.1855E-03  negative regulation of nitrogen compound metabolic process; 4/103; 2.7785E-03 | Proline-rich binding site (e.g., SH3/PxxP) |  |  |  |
| **Feature 206** |  | cellular macromolecule metabolic process; 10/52; 1.6362E-03  protein phosphorylation; 3/52; 2.3088E-03  cellular protein metabolic process; 6/52; 3.3195E-03  phosphorylation; 3/52; 3.3616E-03 | Proline-rich binding site (e.g., SH3/PxxP) |  |  |  |
| **Feature 75** |  |  |  |  | cell wall; 3/51; 1.0966E-03  external encapsulating structure; 3/51; 1.0966E-03 |  |
| **Feature 14** |  | regulation of cell cycle; 4/114; 5.0139E-03  transcription regulator activity; 6/114; 6.1311E-03 | MAPK consensus site (proline directed phosphorylation site) |  |  |  |
| **Feature 140** |  |  | Probable basophilic phosphorylation site |  |  | S/T repeats |
| **Feature 24** |  |  |  |  |  | FG repeats |
| **Feature 147** |  | regulation of GTPase activity; 4/95; 7.6649E-05  regulation of hydrolase activity; 4/95; 6.8717E-04  catalytic activity, acting on DNA; 4/95; 1.2454E-03  catalytic activity, acting on a nucleic acid; 5/95; 7.2714E-03 | Diphenylalanine repeat  �(ER to Golgi transport signal) |  |  |  |
| **Feature 159** |  | positive regulation of catalytic activity; 3/40; 5.3427E-04  positive regulation of molecular function; 3/40; 5.3427E-04  regulation of catalytic activity; 4/40; 7.3237E-04  regulation of molecular function; 4/40; 7.3237E-04 |  |  | plasma membrane; 4/19; 3.7785E-04  membrane; 5/19; 3.7535E-03  cellular anatomical entity; 13/19; 6.1862E-03 |  |
| **Feature 78** |  | cellular bud neck; 3/110; 7.6018E-03 |  |  |  |  |
| **Feature 162** |  | posttranscriptional regulation of gene expression; 5/82; 6.2797E-05  regulation of cellular protein metabolic process; 5/82; 6.2131E-04  regulation of protein metabolic process; 5/82; 6.2131E-04  regulation of mRNA catabolic process; 3/82; 7.6499E-04 |  |  | cellular\_component; 16/20; 2.5297E-03  membrane-bounded organelle; 7/20; 5.9643E-03  intracellular membrane-bounded organelle; 7/20; 5.9643E-03 |  |
| **Feature 114** |  |  | GF-rich region (associated with nucleo-cytoplasmic transport) |  | protein binding; 3/20; 8.0931E-03 |  |
| **Feature 124** |  | transcription regulator activity; 17/418; 4.2070E-05  cellular metabolic process; 27/418; 6.1517E-05  metabolic process; 31/418; 1.8569E-04  macromolecule metabolic process; 20/418; 1.9092E-04 |  |  | nucleic acid binding; 6/28; 1.1403E-03  organic cyclic compound binding; 6/28; 2.4634E-03  heterocyclic compound binding; 6/28; 2.4634E-03 | N/Q rich repeats |
| **Feature 149** |  | phosphotransferase activity, alcohol group as acceptor; 8/138; 9.1443E-04  kinase activity; 8/138; 1.2509E-03  protein kinase activity; 7/138; 2.0451E-03  transferase activity, transferring phosphorus-containing groups; 8/138; 7.2078E-03 |  |  | cytosol; 3/20; 3.8254E-03 |  |
| **Feature 26** |  | monocarboxylic acid transmembrane transporter activity; 3/265; 8.2396E-04  negative regulation of cellular component organization; 3/265; 3.0657E-03  regulation of small GTPase mediated signal transduction; 3/265; 3.0657E-03  regulation of Ras protein signal transduction; 3/265; 3.0657E-03 |  |  | regulation of nitrogen compound metabolic process; 4/20; 5.3810E-03  regulation of primary metabolic process; 4/20; 5.9564E-03  regulation of cellular metabolic process; 4/20; 6.1571E-03  regulation of transcription by RNA polymerase II; 3/20; 7.3640E-03 |  |
| **Feature 222** |  | phosphotransferase activity, alcohol group as acceptor; 9/175; 1.0050E-03  kinase activity; 9/175; 1.4176E-03  protein kinase activity; 8/175; 1.8473E-03  transferase activity, transferring phosphorus-containing groups; 10/175; 2.8946E-03 |  |  |  |  |
| **Feature 60** |  | signal transduction; 7/203; 6.4884E-03  intracellular signal transduction; 6/203; 7.2205E-03 |  |  | cytosol; 3/27; 9.0619E-03 |  |
| **Feature 99** |  |  |  |  |  |  |
| **Feature 167** |  | peptide metabolic process; 3/39; 2.7546E-04  cellular amide metabolic process; 3/39; 4.9539E-04 |  |  |  | A/S/T-repeats |
| **Feature 171** |  | cell wall; 4/116; 1.0459E-03  external encapsulating structure; 4/116; 1.0459E-03  cell surface; 3/116; 1.2429E-03  lipid binding; 3/116; 2.1099E-03 | A-repeats |  |  | A-repeats |
| **Feature 55** |  | protein phosphorylation; 5/177; 1.6574E-03  phosphorylation; 5/177; 3.0805E-03  phosphate-containing compound metabolic process; 8/177; 4.4981E-03  phosphorus metabolic process; 8/177; 6.0032E-03 |  |  |  |  |
| **Feature 83** |  | negative regulation of macromolecule metabolic process; 6/182; 3.3828E-03  negative regulation of metabolic process; 6/182; 6.3255E-03  negative regulation of gene expression; 4/182; 7.5958E-03  positive regulation of nitrogen compound metabolic process; 7/182; 8.2183E-03 |  |  | protein glycosylation; 3/45; 2.0184E-04  macromolecule glycosylation; 3/45; 2.0184E-04  glycosylation; 3/45; 4.2291E-04  organonitrogen compound metabolic process; 7/45; 7.7830E-03 |  |
| **Feature 117** |  | nuclear-transcribed mRNA catabolic process, deadenylation-dependent decay; 3/212; 4.2067E-04  external encapsulating structure; 5/212; 1.1773E-03  cell wall; 5/212; 1.1773E-03  mRNA destabilization; 3/212; 1.5889E-03 |  |  | cell wall; 8/82; 1.6479E-10  external encapsulating structure; 8/82; 1.6479E-10  fungal-type cell wall; 6/82; 9.6983E-08  peptide biosynthetic process; 3/82; 7.6499E-04 |  |
| **Feature 25** |  | ion transport; 3/43; 8.5215E-04  chromosome organization; 3/43; 2.7083E-03 | Ypk1 consensus site RxRxxS/T |  |  |  |
| **Feature 107** |  |  |  |  | ribonucleoprotein complex; 3/20; 8.4733E-03 |  |
| **Feature 119** |  | rRNA processing; 5/88; 1.0394E-03  rRNA metabolic process; 5/88; 1.4783E-03  cytoplasm; 13/88; 3.4749E-03  ncRNA processing; 5/88; 3.6111E-03 |  |  | mitochondrial inner membrane; 3/52; 8.8217E-04  organelle inner membrane; 3/52; 1.1609E-03  mitochondrial membrane; 3/52; 3.3616E-03  structural molecule activity; 3/52; 8.0977E-03 |  |
| **Feature 32** |  | supramolecular complex; 3/76; 6.7988E-03 |  |  |  | S-repeat |
| **Feature 169** |  | movement of cell or subcellular component; 3/100; 4.1232E-04 | Related to basophilic S/T phosphorylation consensus site |  |  |  |
| **Feature 187** |  |  | Related to basophilic S/T phosphorylation consensus site |  | mitochondrion; 6/43; 1.6245E-04  small molecule metabolic process; 5/43; 3.6570E-04  carboxylic acid metabolic process; 3/43; 5.3642E-03  cellular lipid metabolic process; 3/43; 6.7461E-03 |  |
| **Feature 165** |  |  |  |  |  |  |
| **Feature 118** |  |  | Ypk1 consensus site RxRxxS/T |  |  |  |
| **Feature 36** |  | intracellular signal transduction; 3/33; 2.8273E-03  signal transduction; 3/33; 5.7661E-03 | AGC docking motif |  |  |  |
| **Feature 15** |  | organophosphate biosynthetic process; 3/82; 8.4009E-03 |  |  |  |  |
| **Feature 197** |  |  |  |  |  |  |
| **Feature 148** |  | ncRNA processing; 3/35; 8.0793E-03 |  |  |  |  |
| **Feature 210** |  |  |  |  |  |  |
| **Feature 93** |  | positive regulation of catalytic activity; 3/62; 1.9393E-03  positive regulation of molecular function; 3/62; 1.9393E-03 |  |  |  |  |
| **Feature 101** |  |  |  |  |  |  |
| **Feature 245** |  | mitotic cell cycle checkpoint signaling; 3/105; 4.7670E-04  regulation of cell cycle process; 4/105; 4.9160E-04  mitotic cell cycle process; 5/105; 6.4848E-04  regulation of cell cycle phase transition; 3/105; 9.2756E-04 |  |  |  |  |
| **Feature 174** |  |  |  |  |  |  |
| **Feature 22** |  | histone deacetylase complex; 3/82; 2.4615E-03 |  |  | glycosylation; 4/20; 3.7811E-07  protein glycosylation; 3/20; 1.6768E-05  macromolecule glycosylation; 3/20; 1.6768E-05  protein metabolic process; 5/20; 9.0908E-04 |  |
| **Feature 38** |  | chromatin remodeling; 3/50; 1.0346E-03  chromatin organization; 4/50; 2.4605E-03  cellular component organization; 8/50; 5.8505E-03  cellular component organization or biogenesis; 8/50; 7.3140E-03 |  |  | protein metabolic process; 4/19; 5.9652E-03  cellular protein modification process; 3/19; 9.4891E-03  protein modification process; 3/19; 9.4891E-03 |  |
| **Feature 103** |  | regulation of transcription, DNA-templated; 6/58; 2.9767E-03  regulation of nucleic acid-templated transcription; 6/58; 2.9767E-03  regulation of RNA biosynthetic process; 6/58; 3.1961E-03  biological regulation; 11/58; 3.7924E-03 | Similar to WxxLF motif in acidic transcription activation domains (https://www.pnas.org/content/111/34/E3506.short) |  |  | Glutamine repeat |
| **Feature 219** |  | regulation of nucleobase-containing compound metabolic process; 14/196; 1.2568E-03  regulation of RNA metabolic process; 13/196; 2.6393E-03  positive regulation of nucleobase-containing compound metabolic process; 7/196; 3.6443E-03  dephosphorylation; 3/196; 5.6710E-03 |  |  | cellular anatomical entity; 13/20; 8.6360E-03 |  |
| **Feature 48** |  | vesicle-mediated transport; 6/96; 2.6018E-03  endocytosis; 3/96; 2.7755E-03  organelle localization; 3/96; 3.8675E-03  negative regulation of gene expression; 3/96; 8.5566E-03 |  |  |  |  |
| **Feature 216** |  | protein kinase activity; 4/58; 7.0913E-03 |  |  |  |  |
| **Feature 102** |  |  |  |  |  |  |
| **Feature 246** |  | intracellular signal transduction; 8/177; 8.9087E-05  signal transduction; 9/177; 9.9159E-05  transferase activity, transferring phosphorus-containing groups; 12/177; 2.1574E-04  phosphotransferase activity, alcohol group as acceptor; 10/177; 2.3164E-04 |  |  |  |  |
| **Feature 37** |  | catalytic activity, acting on DNA; 5/152; 8.2796E-04  enzyme activator activity; 5/152; 2.6888E-03  molecular function regulator; 5/152; 9.4516E-03  enzyme regulator activity; 5/152; 9.4516E-03 |  |  | mitochondrion; 3/20; 6.6758E-03 |  |
| **Feature 56** |  | transferase activity, transferring phosphorus-containing groups; 5/53; 4.5123E-03  phosphotransferase activity, alcohol group as acceptor; 4/53; 8.0120E-03  kinase activity; 4/53; 9.4089E-03 |  |  | plasma membrane; 7/66; 2.0813E-04 |  |
| **Feature 49** |  |  |  |  | mitochondrion; 6/20; 1.4702E-06  membrane-bounded organelle; 8/20; 1.2231E-03  intracellular membrane-bounded organelle; 8/20; 1.2231E-03  organelle; 8/20; 3.8689E-03 |  |
| **Feature 215** |  |  |  |  | carbohydrate derivative biosynthetic process; 3/20; 1.6768E-05  carbohydrate derivative metabolic process; 3/20; 4.8738E-05  catalytic activity; 6/20; 3.6247E-03  protein metabolic process; 4/20; 7.2292E-03 |  |
| **Feature 146** |  | sulfur compound metabolic process; 3/157; 2.9993E-03 |  |  | mitochondrion; 5/37; 6.9515E-04  cellular anatomical entity; 23/37; 1.4438E-03  cellular\_component; 25/37; 7.7098E-03  membrane-bounded organelle; 10/37; 8.6114E-03 |  |
| **Feature 193** |  | chromatin organization; 7/172; 3.0871E-03  chromatin remodeling; 4/172; 4.5110E-03  cellular process; 59/172; 4.7414E-03  cellular response to nutrient levels; 3/172; 9.9719E-03 |  |  |  |  |
| **Feature 64** |  | chromosome organization; 3/46; 3.2889E-03 |  |  | catalytic activity; 9/44; 6.7189E-03 |  |
| **Feature 2** |  | mRNA binding; 4/85; 1.7102E-03  RNA binding; 7/85; 2.4975E-03  protein ubiquitination; 3/85; 3.6722E-03  protein modification by small protein conjugation; 3/85; 3.6722E-03 |  |  |  |  |
| **Feature 253** |  | endosome; 4/107; 1.4675E-03  vesicle; 4/107; 4.9066E-03  cytoplasmic vesicle; 4/107; 4.9066E-03  intracellular vesicle; 4/107; 4.9066E-03 |  |  | mitochondrion; 5/21; 4.1368E-05 |  |
| **Feature 63** |  | positive regulation of catalytic activity; 5/111; 5.5137E-05  positive regulation of molecular function; 5/111; 5.5137E-05  regulation of catalytic activity; 7/111; 1.1721E-04  regulation of molecular function; 7/111; 1.1721E-04 |  |  | ribosomal subunit; 5/58; 1.3070E-06  mitochondrion; 8/58; 1.2979E-05  small ribosomal subunit; 3/58; 3.2677E-05  intrinsic component of mitochondrial membrane; 3/58; 4.3128E-04 |  |
| **Feature 85** |  | RNA binding; 15/254; 3.2456E-04  exocyst; 3/254; 6.3162E-03  cellular anatomical entity; 110/254; 8.9067E-03  regulation of biological quality; 9/254; 9.1011E-03 |  |  |  | RGG-repeat |
| **Feature 68** |  | lipid biosynthetic process; 3/59; 6.6631E-03 |  |  | mitochondrial protein-containing complex; 5/74; 3.8070E-05  membrane protein complex; 6/74; 6.3804E-05  inner mitochondrial membrane protein complex; 3/74; 3.2897E-04  organic acid biosynthetic process; 3/74; 1.3050E-03 |  |
| **Feature 23** |  |  |  |  | organelle membrane; 3/20; 4.0677E-03  membrane; 5/20; 4.7729E-03 |  |
| **Feature 71** |  |  |  |  |  |  |
| **Feature 189** |  | glycosylation; 3/92; 3.4251E-03  regulation of transcription, DNA-templated; 7/92; 7.6223E-03  regulation of nucleic acid-templated transcription; 7/92; 7.6223E-03  regulation of RNA biosynthetic process; 7/92; 8.2227E-03 |  |  | catalytic activity; 6/20; 3.6247E-03 |  |
| **Feature 92** |  |  |  |  |  |  |
| **Feature 28** |  | rRNA processing; 4/59; 1.8397E-03  rRNA metabolic process; 4/59; 2.4421E-03  ncRNA processing; 4/59; 5.0373E-03  nucleic acid metabolic process; 8/59; 8.5343E-03 |  |  | organelle membrane; 3/20; 4.0677E-03  membrane; 5/20; 4.7729E-03 |  |
| **Feature 52** |  |  |  |  | mitochondrion; 6/25; 6.2209E-06  cellular metabolic process; 8/25; 7.0059E-03 |  |
| **Feature 0** |  |  |  |  | protein glycosylation; 3/26; 3.7937E-05  macromolecule glycosylation; 3/26; 3.7937E-05  glycosylation; 3/26; 8.0301E-05  mitochondrion; 4/26; 1.5129E-03 |  |
| **Feature 84** |  | protein metabolic process; 19/183; 8.2623E-05  glycosylation; 5/183; 2.1134E-04  organonitrogen compound metabolic process; 21/183; 4.2240E-04  metabolic process; 40/183; 5.1615E-04 |  |  | protein glycosylation; 4/20; 1.2719E-07  macromolecule glycosylation; 4/20; 1.2719E-07  glycosylation; 4/20; 3.7811E-07  cellular protein metabolic process; 6/20; 1.3185E-05 |  |
| **Feature 65** |  | protein binding; 8/113; 2.3373E-03  histone binding; 3/113; 4.4239E-03  cytoplasm; 15/113; 5.4461E-03  binding; 19/113; 5.4631E-03 | Di-arginine |  | cytoplasm; 5/20; 7.2727E-03 | R/G repeats |
| **Feature 87** |  | histone binding; 3/51; 4.3513E-04 | Double PKA phosphorylation sequence |  | glycosylation; 3/20; 3.5607E-05  mitochondrion; 4/20; 5.3584E-04 |  |
| **Feature 141** |  |  |  |  | mitochondrion; 5/20; 3.2001E-05  intracellular membrane-bounded organelle; 7/20; 5.9643E-03  membrane-bounded organelle; 7/20; 5.9643E-03  protein metabolic process; 4/20; 7.2292E-03 |  |
| **Feature 53** |  |  |  |  | protein glycosylation; 3/44; 1.8864E-04  macromolecule glycosylation; 3/44; 1.8864E-04  glycosylation; 3/44; 3.9545E-04  organonitrogen compound metabolic process; 8/44; 1.6206E-03 |  |
| **Feature 115** |  | mitochondrion; 8/141; 5.9526E-03 |  |  | mitochondrion; 4/20; 5.3584E-04  ribonucleoprotein complex; 3/20; 8.4733E-03 |  |
| **Feature 195** |  |  | RxL cyclin docking site |  |  |  |
| **Feature 131** |  | regulation of GTPase activity; 3/86; 1.3773E-03  glycosylation; 3/86; 2.8231E-03  regulation of hydrolase activity; 3/86; 6.2960E-03 |  |  | mitochondrion; 3/20; 6.6758E-03 |  |
| **Feature 31** |  | carbohydrate derivative biosynthetic process; 3/120; 3.6089E-03  phosphotransferase activity, alcohol group as acceptor; 6/120; 8.8202E-03  carbohydrate derivative metabolic process; 3/120; 9.6799E-03 |  |  |  |  |
| **Feature 7** |  | catalytic activity, acting on a protein; 13/181; 8.7534E-04  protein glycosylation; 4/181; 9.4112E-04  macromolecule glycosylation; 4/181; 9.4112E-04  ubiquitin-like protein transferase activity; 3/181; 9.9508E-04 |  |  | organonitrogen compound metabolic process; 6/20; 3.8458E-04  protein metabolic process; 5/20; 9.0908E-04  protein modification process; 4/20; 1.0632E-03  cellular protein modification process; 4/20; 1.0632E-03 |  |
| **Feature 57** |  | nitrogen compound metabolic process; 12/53; 5.0981E-03  integral component of organelle membrane; 3/53; 6.5842E-03  intrinsic component of organelle membrane; 3/53; 7.5233E-03 | D-box APC destruction motif |  | glycosylation; 3/20; 3.5607E-05  mitochondrion; 4/20; 5.3584E-04  protein metabolic process; 4/20; 7.2292E-03  cellular macromolecule metabolic process; 5/20; 7.9749E-03 |  |
| **Feature 247** |  | glycosylation; 4/159; 1.5707E-03  protein glycosylation; 3/159; 8.0080E-03  macromolecule glycosylation; 3/159; 8.0080E-03 |  |  | glycosylation; 4/20; 3.7811E-07  protein glycosylation; 3/20; 1.6768E-05  macromolecule glycosylation; 3/20; 1.6768E-05  protein metabolic process; 6/20; 8.9271E-05 |  |
| **Feature 86** |  |  |  |  | protein glycosylation; 3/20; 1.6768E-05  macromolecule glycosylation; 3/20; 1.6768E-05  glycosylation; 3/20; 3.5607E-05  protein modification process; 5/20; 7.7003E-05 |  |
| **Feature 13** |  | Golgi apparatus; 3/124; 3.9635E-03  glycosylation; 3/124; 7.9600E-03 |  |  | Golgi apparatus; 3/20; 1.6768E-05  intracellular membrane-bounded organelle; 7/20; 5.9643E-03  membrane-bounded organelle; 7/20; 5.9643E-03 |  |
| **Feature 62** |  |  | Matches part of Pex3 docking motif |  | glycosylation; 3/56; 8.0922E-04 |  |
| **Feature 132** |  | mitochondrial matrix; 3/84; 2.4509E-04  membrane-enclosed lumen; 3/84; 8.2129E-04  organelle lumen; 3/84; 8.2129E-04  intracellular organelle lumen; 3/84; 8.2129E-04 | Matches part of Pex3 docking motif |  | protein glycosylation; 3/20; 1.6768E-05  macromolecule glycosylation; 3/20; 1.6768E-05  glycosylation; 3/20; 3.5607E-05  protein metabolic process; 6/20; 8.9271E-05 |  |
| **Feature 157** |  | cellular protein metabolic process; 4/20; 2.1642E-03  organic substance metabolic process; 7/20; 6.0653E-03  cellular metabolic process; 7/20; 6.6989E-03  protein metabolic process; 4/20; 7.2292E-03 |  |  | glycosylation; 4/20; 3.7811E-07  protein glycosylation; 3/20; 1.6768E-05  macromolecule glycosylation; 3/20; 1.6768E-05  protein metabolic process; 6/20; 8.9271E-05 |  |
| **Feature 235** |  | cell wall macromolecule metabolic process; 3/62; 9.8449E-05  cell wall macromolecule biosynthetic process; 3/62; 9.8449E-05  cellular component macromolecule biosynthetic process; 3/62; 9.8449E-05  glycosylation; 3/62; 1.0917E-03 |  |  | protein glycosylation; 3/20; 1.6768E-05  carbohydrate derivative biosynthetic process; 3/20; 1.6768E-05  macromolecule glycosylation; 3/20; 1.6768E-05  glycosylation; 3/20; 3.5607E-05 |  |
| **Feature 225** |  | cell wall macromolecule metabolic process; 3/93; 3.3215E-04  cell wall macromolecule biosynthetic process; 3/93; 3.3215E-04  cellular component macromolecule biosynthetic process; 3/93; 3.3215E-04  biological\_process; 45/93; 5.7030E-04 |  |  | protein glycosylation; 3/20; 1.6768E-05  carbohydrate derivative biosynthetic process; 3/20; 1.6768E-05  macromolecule glycosylation; 3/20; 1.6768E-05  glycosylation; 3/20; 3.5607E-05 |  |
| **Feature 134** |  |  |  |  | mitochondrion; 5/44; 1.5578E-03  integral component of membrane; 6/44; 1.6719E-03  intrinsic component of membrane; 6/44; 2.3874E-03  integral component of organelle membrane; 3/44; 3.8866E-03 |  |
| **Feature 168** |  | catalytic activity; 23/151; 1.8182E-03  protein-containing complex; 5/151; 5.8682E-03 |  |  | organonitrogen compound metabolic process; 10/44; 5.9151E-05  protein glycosylation; 3/44; 1.8864E-04  macromolecule glycosylation; 3/44; 1.8864E-04  protein metabolic process; 8/44; 2.7681E-04 |  |
| **Feature 243** |  | protein glycosylation; 4/86; 5.1666E-05  macromolecule glycosylation; 4/86; 5.1666E-05  glycosylation; 4/86; 1.4788E-04  organonitrogen compound metabolic process; 11/86; 4.4338E-03 |  |  | mitochondrion; 4/35; 4.6398E-03  organonitrogen compound metabolic process; 6/35; 8.4169E-03 |  |
| **Feature 145** |  | structural constituent of ribosome; 3/20; 8.3715E-05  structural molecule activity; 3/20; 4.9538E-04 |  |  | membrane protein complex; 3/20; 8.9580E-04 |  |
| **Feature 116** |  | cellular response to stimulus; 6/120; 9.8653E-03 |  |  | mitochondrion; 3/20; 6.6758E-03 |  |
| **Feature 233** |  |  |  |  | intrinsic component of organelle membrane; 3/34; 2.1158E-03  membrane protein complex; 3/34; 4.2837E-03 |  |
| **Feature 94** |  | ATPase-dependent activity; 4/69; 1.1882E-03  GTPase activator activity; 3/69; 2.6415E-03  GTPase regulator activity; 3/69; 3.3740E-03  nucleoside-triphosphatase regulator activity; 3/69; 3.3740E-03 |  |  | mitochondrion; 8/54; 7.4925E-06  mitochondrial matrix; 3/54; 6.4847E-05  inner mitochondrial membrane protein complex; 3/54; 1.2793E-04  mitochondrial protein-containing complex; 4/54; 1.8518E-04 |  |
| **Feature 166** |  | actin cytoskeleton organization; 3/117; 2.7108E-04  actin filament-based process; 3/117; 6.5716E-04  catalytic activity, acting on a protein; 10/117; 9.7423E-04  catalytic activity; 19/117; 2.4211E-03 |  |  | organonitrogen compound metabolic process; 6/20; 3.8458E-04  organic substance metabolic process; 7/20; 6.0653E-03  cellular metabolic process; 7/20; 6.6989E-03  metabolic process; 7/20; 9.3227E-03 |  |
| **Feature 214** |  |  |  |  | organonitrogen compound metabolic process; 10/46; 8.8777E-05  protein glycosylation; 3/46; 2.1563E-04  carbohydrate derivative biosynthetic process; 3/46; 2.1563E-04  macromolecule glycosylation; 3/46; 2.1563E-04 |  |
| **Feature 144** |  |  |  |  | membrane protein complex; 4/21; 4.1659E-05  mitochondrion; 3/21; 7.6797E-03 |  |
| **Feature 177** |  | cytoplasmic ribonucleoprotein granule; 3/106; 1.6235E-03  ribonucleoprotein granule; 3/106; 1.6235E-03  transcription regulator activity; 6/106; 4.2854E-03  DNA-binding transcription factor activity, RNA polymerase II-specific; 4/106; 8.2078E-03 |  |  | ribosomal subunit; 5/67; 2.7102E-06  mitochondrial protein-containing complex; 5/67; 2.3359E-05  biological\_process; 8/67; 2.6718E-04  mitochondrion; 7/67; 2.8878E-04 |  |
| **Feature 139** |  | phosphate-containing compound metabolic process; 7/94; 4.4390E-04  phosphorus metabolic process; 7/94; 5.9192E-04  transcription regulator complex; 4/94; 2.4840E-03  glycerophospholipid metabolic process; 3/94; 3.6421E-03 |  |  | mitochondrial membrane; 4/65; 4.9051E-04  cellular amino acid metabolic process; 4/65; 6.1978E-04  alpha-amino acid metabolic process; 3/65; 8.9225E-04  carboxylic acid metabolic process; 4/65; 1.9393E-03 |  |
| **Feature 192** |  | transcription factor TFIID complex; 3/176; 9.1572E-04  RNA polymerase II transcription regulator complex; 5/176; 1.6155E-03  protein kinase activity; 8/176; 1.9164E-03  cellular bud neck; 4/176; 3.4345E-03 |  |  | mitochondrial membrane; 4/58; 3.1552E-04 |  |
| **Feature 73** |  | mRNA transport; 3/83; 1.8229E-03  mRNA export from nucleus; 3/83; 1.8229E-03  RNA transport; 3/83; 2.5490E-03  RNA export from nucleus; 3/83; 2.5490E-03 |  |  | protein metabolic process; 4/20; 7.2292E-03 |  |
| **Feature 30** |  | DNA-binding transcription factor activity, RNA polymerase II-specific; 4/68; 1.6370E-03  DNA-binding transcription factor activity; 4/68; 3.5863E-03 |  |  | mitochondrion; 3/20; 6.6758E-03 |  |
| **Feature 34** |  | glycosylation; 4/120; 5.3906E-04  protein glycosylation; 3/120; 3.6089E-03  macromolecule glycosylation; 3/120; 3.6089E-03 |  |  | glycosylation; 3/21; 4.1464E-05 |  |
| **Feature 98** |  | cell wall macromolecule metabolic process; 3/51; 5.4533E-05  cell wall macromolecule biosynthetic process; 3/51; 5.4533E-05  cellular component macromolecule biosynthetic process; 3/51; 5.4533E-05  small molecule binding; 4/51; 5.4831E-04 |  |  | catalytic activity; 6/20; 3.6247E-03 |  |
| **Feature 50** |  | mannan polymerase complex; 3/51; 5.5955E-06  mannosyltransferase complex; 3/51; 5.5955E-06  membrane protein complex; 5/51; 1.1434E-04  cellular biosynthetic process; 6/51; 2.0250E-03 |  |  |  |  |
| **Feature 120** |  | glycosylation; 4/59; 3.3212E-05  protein glycosylation; 3/59; 4.5377E-04  macromolecule glycosylation; 3/59; 4.5377E-04 |  |  |  |  |
| **Feature 35** |  |  |  |  |  |  |
| **Feature 121** |  | hydrolase activity; 9/95; 8.4659E-04  glycosylation; 3/95; 3.7538E-03  fungal-type cell wall organization; 3/95; 8.3116E-03 |  |  |  |  |
| **Feature 130** |  | glycerophospholipid biosynthetic process; 3/74; 5.6483E-04  lipid biosynthetic process; 4/74; 1.2614E-03  glycerolipid biosynthetic process; 3/74; 1.3050E-03  phospholipid biosynthetic process; 3/74; 1.3050E-03 |  |  |  |  |
| **Feature 238** |  | catalytic activity; 11/54; 2.9052E-03  catalytic activity, acting on a protein; 6/54; 2.9166E-03  hydrolase activity; 6/54; 2.9166E-03 |  |  |  |  |
| **Feature 161** |  | glycosylation; 6/76; 6.1004E-08  protein glycosylation; 4/76; 3.1579E-05  macromolecule glycosylation; 4/76; 3.1579E-05  transferase complex; 5/76; 5.4099E-03 |  |  | protein metabolic process; 5/20; 9.0908E-04  organonitrogen compound metabolic process; 5/20; 2.9630E-03  cellular macromolecule metabolic process; 5/20; 7.9749E-03 |  |
| **Feature 200** |  | catalytic activity, acting on a protein; 6/58; 4.1966E-03  catalytic activity; 11/58; 5.1960E-03 |  |  | catalytic activity; 6/20; 3.6247E-03 |  |
| **Feature 218** |  | glycosylation; 5/135; 4.8751E-05  protein modification process; 9/135; 4.0735E-03  cellular protein modification process; 9/135; 4.0735E-03  protein glycosylation; 3/135; 5.0484E-03 |  |  | catalytic activity; 6/20; 3.6247E-03  protein metabolic process; 4/20; 7.2292E-03 |  |
| **Feature 199** |  | negative regulation of gene expression; 5/118; 1.1649E-04  nuclear-transcribed mRNA catabolic process; 4/118; 1.7986E-04  mRNA catabolic process; 4/118; 3.1330E-04  negative regulation of biological process; 7/118; 9.5410E-04 |  |  | membrane protein complex; 3/20; 8.9580E-04  catalytic activity; 6/20; 3.6247E-03 |  |
| **Feature 201** |  | transcription regulator activity; 3/20; 2.9459E-03  protein metabolic process; 4/20; 7.2292E-03 | Acidic-phenylalanine stretches associated with �γ-ear interaction� (https://doi.org/10.1038/ncb901) |  |  |  |
| **Feature 172** |  | Golgi apparatus; 3/110; 2.8114E-03  transmembrane transporter activity; 7/110; 8.7285E-03 |  |  |  |  |
| **Feature 158** |  | Golgi apparatus; 3/75; 9.2240E-04  protein modification process; 6/75; 8.0145E-03  cellular protein modification process; 6/75; 8.0145E-03  protein metabolic process; 8/75; 9.3428E-03 | Lysomal/Golgi trafficking signal |  | carbohydrate derivative biosynthetic process; 3/31; 6.5150E-05  carbohydrate derivative metabolic process; 3/31; 1.8770E-04  catalytic activity; 9/31; 4.7804E-04  cellular localization; 4/31; 8.8595E-03 |  |
| **Feature 12** |  | cytosol; 7/133; 5.1044E-03 |  |  | macromolecule modification; 4/20; 2.4755E-03 |  |
| **Feature 155** |  |  |  |  | endoplasmic reticulum; 3/23; 1.0942E-03 |  |
| **Feature 122** |  | cellular metabolic process; 6/129; 5.3281E-03  organic substance metabolic process; 6/129; 7.4582E-03  cellular process; 20/129; 9.2316E-03 |  |  |  |  |
| **Feature 20** |  |  | Potential LP motif/cyclin docking site (https://doi.org/10.1016/j.cub.2020.08.099) |  | mitochondrion; 5/49; 2.5361E-03  catalytic activity; 10/49; 4.4137E-03  organonitrogen compound biosynthetic process; 3/49; 8.6938E-03 |  |
| **Feature 190** |  | oxidoreductase activity; 4/102; 5.0191E-03  DNA-binding transcription factor activity, RNA polymerase II-specific; 4/102; 7.1679E-03 |  |  |  |  |
| **Feature 249** |  | regulation of transcription by RNA polymerase II; 8/82; 2.1459E-04  regulation of cellular process; 13/82; 5.3369E-04  site of polarized growth; 4/82; 5.3469E-04  regulation of cellular macromolecule biosynthetic process; 9/82; 7.1375E-04 | Potential LP motif/cyclin docking site (https://doi.org/10.1016/j.cub.2020.08.099) |  | mitochondrion; 3/20; 6.6758E-03 |  |
| **Feature 226** |  | cellular homeostasis; 5/128; 3.1125E-03  homeostatic process; 5/128; 3.1125E-03  transferase activity, transferring phosphorus-containing groups; 8/128; 4.5761E-03  cellular metal ion homeostasis; 4/128; 9.2625E-03 |  |  | membrane protein complex; 3/20; 8.9580E-04  mitochondrion; 3/20; 6.6758E-03 |  |
| **Feature 217** |  | protein modification process; 10/149; 2.3195E-03  cellular protein modification process; 10/149; 2.3195E-03  mediator complex; 3/149; 2.5782E-03  cellular protein metabolic process; 11/149; 3.0639E-03 |  |  | protein glycosylation; 3/38; 1.2113E-04  macromolecule glycosylation; 3/38; 1.2113E-04  glycosylation; 3/38; 2.5475E-04  protein metabolic process; 6/38; 3.5216E-03 |  |
| **Feature 175** |  | protein modification process; 5/40; 2.2609E-03  cellular protein modification process; 5/40; 2.2609E-03  cellular protein metabolic process; 5/40; 5.1849E-03  macromolecule modification; 5/40; 6.0555E-03 |  |  |  |  |
| **Feature 4** |  | protein kinase activity; 7/136; 1.8789E-03  phosphotransferase activity, alcohol group as acceptor; 7/136; 3.9205E-03  protein modification by small protein conjugation or removal; 4/136; 4.6799E-03  kinase activity; 7/136; 5.0936E-03 |  |  | acyltransferase activity; 3/20; 6.4689E-05  transferase activity; 4/20; 6.1571E-03 |  |
| **Feature 1** |  | nitrogen compound metabolic process; 19/85; 6.4457E-04  macromolecule metabolic process; 18/85; 9.5065E-04  primary metabolic process; 20/85; 1.2061E-03  organic substance metabolic process; 20/85; 1.8715E-03 |  |  | protein glycosylation; 4/53; 7.4016E-06  macromolecule glycosylation; 4/53; 7.4016E-06  glycosylation; 4/53; 2.1591E-05  protein metabolic process; 8/53; 1.0235E-03 |  |
| **Feature 179** |  | intrinsic component of membrane; 5/42; 9.9595E-03 |  |  |  |  |
| **Feature 54** |  | protein kinase activity; 8/174; 1.7803E-03  transferase activity, transferring phosphorus-containing groups; 10/174; 2.7743E-03  phosphotransferase activity, alcohol group as acceptor; 8/174; 4.0485E-03  kinase activity; 8/174; 5.4133E-03 |  |  | membrane protein complex; 3/22; 1.1941E-03 |  |
| **Feature 59** |  | regulation of transcription, DNA-templated; 4/27; 4.1953E-03  regulation of nucleic acid-templated transcription; 4/27; 4.1953E-03  regulation of RNA biosynthetic process; 4/27; 4.4131E-03  regulation of RNA metabolic process; 4/27; 6.1558E-03 |  |  | protein modification process; 4/20; 1.0632E-03  cellular protein modification process; 4/20; 1.0632E-03  cellular protein metabolic process; 4/20; 2.1642E-03  macromolecule modification; 4/20; 2.4755E-03 |  |
| **Feature 76** |  | phosphatase activity; 3/83; 5.6984E-03  phosphoric ester hydrolase activity; 3/83; 7.0997E-03 |  |  | mitochondrion; 6/56; 7.0877E-04  catalytic activity; 12/56; 1.1848E-03  phosphatase activity; 3/56; 1.8485E-03  membrane protein complex; 4/56; 2.0117E-03 |  |
| **Feature 72** |  |  | glucose or fucose modification site |  |  |  |
| **Feature 108** |  |  | Contains strong CDK/Cdc28 consensus phosphorylation site [ST]P.[KR] |  | organonitrogen compound metabolic process; 6/27; 2.1674E-03  nitrogen compound metabolic process; 8/27; 3.7942E-03  primary metabolic process; 8/27; 7.3471E-03 |  |
| **Feature 127** |  | mitochondrion; 4/41; 8.2158E-03  transferase activity, transferring phosphorus-containing groups; 4/41; 9.8657E-03 |  |  | ribonucleoprotein complex; 3/20; 8.4733E-03 |  |
| **Feature 46** |  |  | CAAX box prenylation motif |  | glycosylation; 3/20; 3.5607E-05  protein metabolic process; 4/20; 7.2292E-03 |  |
| **Feature 137** |  | cytoskeleton organization; 5/147; 3.3119E-04  organelle organization; 11/147; 6.2644E-04  microtubule cytoskeleton organization; 3/147; 2.4792E-03  microtubule-based process; 3/147; 6.4234E-03 | CDK/Pho85 proline-directed   phosphorylation consensus site |  |  |  |
| **Feature 207** |  | kinase activity; 8/94; 8.8866E-05  phosphotransferase activity, alcohol group as acceptor; 7/94; 4.4390E-04  transferase activity, transferring phosphorus-containing groups; 8/94; 6.2208E-04  protein kinase activity; 6/94; 1.3692E-03 |  |  | catalytic activity; 12/57; 1.3966E-03  mitochondrion; 5/57; 4.9351E-03 |  |
| **Feature 51** |  |  |  |  | catalytic activity; 11/42; 3.0672E-04  intrinsic component of membrane; 5/42; 9.9595E-03 |  |
| **Feature 45** |  |  |  |  | cellular anatomical entity; 13/20; 8.6360E-03 |  |
| **Feature 105** |  |  |  |  | cell wall; 5/29; 6.0738E-08  external encapsulating structure; 5/29; 6.0738E-08  intrinsic component of membrane; 7/29; 2.3662E-05  cellular anatomical entity; 21/29; 1.0054E-04 |  |
| **Feature 152** |  | cytoplasm; 25/222; 4.0854E-03 |  |  |  |  |
| **Feature 227** |  | cellular response to DNA damage stimulus; 4/93; 5.1590E-03 |  |  | external encapsulating structure; 6/82; 4.0719E-07  cell wall; 6/82; 4.0719E-07  fungal-type cell wall; 5/82; 4.1594E-06  anchored component of membrane; 3/82; 2.2804E-04 |  |
| **Feature 163** |  | catabolic process; 10/146; 7.2097E-04  organic substance catabolic process; 9/146; 8.5104E-04  macromolecule catabolic process; 8/146; 1.1277E-03  regulation of organelle organization; 3/146; 1.2632E-03 |  |  | protein-containing complex; 3/126; 3.9677E-03  localization within membrane; 3/126; 6.0210E-03 |  |
| **Feature 184** |  | carbohydrate derivative metabolic process; 3/50; 7.8575E-04  fungal-type cell wall organization; 3/50; 1.3282E-03  cell wall organization; 3/50; 1.6694E-03  external encapsulating structure organization; 3/50; 1.6694E-03 |  |  | mitochondrion; 4/34; 4.1705E-03 |  |
| **Feature 153** |  | protein phosphorylation; 5/153; 8.5331E-04  signal transduction; 7/153; 1.2946E-03  phosphorylation; 5/153; 1.6091E-03  intracellular signal transduction; 6/153; 1.7417E-03 |  |  | ribosomal subunit; 5/288; 2.9664E-03  mitochondrial protein-containing complex; 6/288; 3.5764E-03 |  |
| **Feature 178** |  |  |  |  | transcription coregulator activity; 6/251; 7.6681E-04  transcription coactivator activity; 3/251; 6.1046E-03  transcription regulator activity; 9/251; 9.9197E-03 |  |
| **Feature 188** |  |  |  |  | cellular process; 12/23; 5.7762E-03  cellular aromatic compound metabolic process; 5/23; 7.0942E-03  heterocycle metabolic process; 5/23; 7.0942E-03  RNA metabolic process; 4/23; 7.3316E-03 |  |
| **Feature 104** |  | negative regulation of cellular macromolecule biosynthetic process; 4/82; 9.2981E-04  negative regulation of macromolecule biosynthetic process; 4/82; 9.2981E-04  negative regulation of nitrogen compound metabolic process; 4/82; 1.1892E-03  regulation of RNA metabolic process; 8/82; 1.7773E-03 |  |  |  |  |
| **Feature 70** |  |  |  |  | primary metabolic process; 8/20; 8.4082E-04  protein metabolic process; 5/20; 9.0908E-04  protein modification process; 4/20; 1.0632E-03  cellular protein modification process; 4/20; 1.0632E-03 |  |
| **Feature 113** |  | transcription regulator complex; 3/51; 3.7671E-03  negative regulation of cellular metabolic process; 3/51; 5.1303E-03  negative regulation of macromolecule metabolic process; 3/51; 8.6563E-03 |  |  | protein glycosylation; 3/20; 1.6768E-05  macromolecule glycosylation; 3/20; 1.6768E-05  glycosylation; 3/20; 3.5607E-05  protein metabolic process; 5/20; 9.0908E-04 |  |
| **Feature 96** |  | cell cycle process; 6/88; 9.6695E-04  chromosome organization; 4/88; 2.4066E-03  meiotic cell cycle process; 3/88; 5.2835E-03 |  |  |  |  |
| **Feature 27** |  | mRNA transport; 3/47; 3.4100E-04  mRNA export from nucleus; 3/47; 3.4100E-04  RNA transport; 3/47; 4.8144E-04  RNA export from nucleus; 3/47; 4.8144E-04 |  |  | rRNA processing; 4/42; 5.0344E-04  rRNA metabolic process; 4/42; 6.7473E-04  ncRNA processing; 4/42; 1.4323E-03  ncRNA metabolic process; 4/42; 3.7635E-03 |  |
| **Feature 221** |  |  |  |  |  |  |
| **Feature 176** |  | RNA modification; 6/288; 5.0606E-03  mRNA transport; 4/288; 8.8935E-03  mRNA export from nucleus; 4/288; 8.8935E-03  phosphatidylinositol metabolic process; 3/288; 9.0430E-03 |  |  | nucleolus; 3/21; 1.6880E-03 |  |
| **Feature 19** |  |  |  |  |  |  |
| **Feature 138** |  |  |  |  | mRNA transport; 3/20; 2.5038E-05  mRNA export from nucleus; 3/20; 2.5038E-05  RNA transport; 3/20; 3.5607E-05  RNA export from nucleus; 3/20; 3.5607E-05 |  |
| **Feature 194** |  | regulation of macromolecule metabolic process; 12/119; 2.6254E-03  regulation of metabolic process; 12/119; 3.5120E-03  mRNA catabolic process; 3/119; 5.1233E-03  regulation of gene expression; 10/119; 5.9697E-03 |  |  | chromatin remodeling; 5/177; 5.1053E-04  cellular process; 62/177; 2.1952E-03  establishment of protein localization to vacuole; 3/177; 2.2205E-03  protein localization to vacuole; 3/177; 2.2205E-03 |  |
| **Feature 6** |  |  |  |  |  |  |
| **Feature 241** |  |  |  |  |  |  |
| **Feature 8** |  | omega peptidase activity; 3/200; 1.3375E-03 |  |  |  |  |
| **Feature 242** |  | histone deacetylase complex; 3/77; 2.0525E-03 |  |  |  |  |
| **Feature 150** |  |  |  |  | nucleolus; 4/35; 6.3318E-04  RNA binding; 4/35; 7.0750E-03  intracellular non-membrane-bounded organelle; 4/35; 7.4806E-03  non-membrane-bounded organelle; 4/35; 7.4806E-03 |  |
| **Feature 61** |  | supramolecular complex; 7/276; 2.5925E-04  cytoplasmic stress granule; 3/276; 9.3131E-04  monovalent inorganic cation homeostasis; 3/276; 9.3131E-04  cellular monovalent inorganic cation homeostasis; 3/276; 9.3131E-04 |  |  |  |  |
| **Feature 186** |  | preribosome; 5/147; 2.3184E-03  transcription regulator activity; 7/147; 5.2750E-03  small-subunit processome; 3/147; 9.2698E-03 |  |  | ribonucleoprotein complex; 4/28; 2.7621E-03  nucleolus; 3/28; 3.9318E-03 |  |
| **Feature 47** |  | recombinational repair; 3/305; 4.6274E-03  actin cytoskeleton organization; 3/305; 4.6274E-03  localization; 6/305; 5.5707E-03  transport; 5/305; 5.6361E-03 |  |  | mitochondrion; 3/20; 6.6758E-03 |  |
| **Feature 111** |  | protein deubiquitination; 3/199; 1.3178E-03  protein modification by small protein removal; 3/199; 1.3178E-03  microtubule cytoskeleton organization; 3/199; 5.9219E-03 |  |  | nucleolus; 5/47; 1.7410E-04  intracellular non-membrane-bounded organelle; 6/47; 5.6179E-04  non-membrane-bounded organelle; 6/47; 5.6179E-04  ribonucleoprotein complex; 5/47; 3.0770E-03 |  |
| **Feature 3** |  | double-stranded DNA binding; 9/187; 1.5224E-04  sequence-specific DNA binding; 8/187; 1.0670E-03  transcription regulatory region nucleic acid binding; 7/187; 1.1310E-03  transcription cis-regulatory region binding; 7/187; 1.1310E-03 | Bi-partite cNLS |  | ribonucleoprotein complex; 3/20; 8.4733E-03 |  |
| **Feature 170** |  |  |  |  | ribosomal subunit; 3/48; 6.9637E-04  ribonucleoprotein complex; 5/48; 3.3783E-03 |  |
| **Feature 198** |  | TORC2 complex; 3/211; 3.7009E-03  TOR complex; 3/211; 6.9944E-03  positive regulation of transcription by RNA polymerase II; 5/211; 8.5937E-03 |  |  | mitochondrion; 3/22; 8.7683E-03 |  |
| **Feature 123** |  | movement of cell or subcellular component; 3/149; 1.3410E-03  chromatin organization; 6/149; 6.6843E-03 |  |  | protein-containing complex; 6/20; 7.4409E-03  ribonucleoprotein complex; 3/20; 8.4733E-03 |  |
| **Feature 10** |  | regulation of cellular metabolic process; 6/41; 4.1573E-03  regulation of macromolecule metabolic process; 6/41; 5.4310E-03  regulation of metabolic process; 6/41; 6.4269E-03  regulation of cellular macromolecule biosynthetic process; 5/41; 7.4400E-03 |  |  | ribonucleoprotein complex; 4/24; 1.5294E-03 |  |
| **Feature 248** |  | regulation of cellular macromolecule biosynthetic process; 15/144; 1.9419E-05  regulation of macromolecule biosynthetic process; 15/144; 2.5877E-05  regulation of cellular biosynthetic process; 15/144; 3.4166E-05  regulation of biosynthetic process; 15/144; 3.4166E-05 |  |  | mitochondrion; 6/48; 3.0284E-04 |  |
| **Feature 67** |  | nucleus; 36/249; 2.9346E-05  nucleic acid binding; 25/249; 6.4632E-05  heterocyclic compound binding; 27/249; 1.2279E-04  organic cyclic compound binding; 27/249; 1.2279E-04 | basic nuclear localization signal |  | nucleolus; 4/55; 3.4965E-03  catalytic activity, acting on a nucleic acid; 4/55; 5.3264E-03  RNA binding; 5/55; 7.0115E-03  non-membrane-bounded organelle; 5/55; 7.4941E-03 |  |
| **Feature 11** |  | ncRNA processing; 8/143; 2.1377E-04  rRNA processing; 7/143; 2.1865E-04  rRNA metabolic process; 7/143; 3.6122E-04  RNA binding; 10/143; 1.0436E-03 |  |  | nucleolus; 5/30; 1.8640E-05  intracellular non-membrane-bounded organelle; 5/30; 4.7516E-04  non-membrane-bounded organelle; 5/30; 4.7516E-04  RNA binding; 4/30; 4.0255E-03 | Lysine repeats |
| **Feature 181** |  | rRNA processing; 11/224; 1.6754E-06  rRNA metabolic process; 11/224; 4.0333E-06  ncRNA processing; 12/224; 5.4293E-06  RNA processing; 13/224; 8.9594E-05 |  |  | ribonucleoprotein complex; 6/25; 1.0331E-05  nucleolus; 4/25; 1.6616E-04  non-membrane-bounded organelle; 4/25; 2.1422E-03  intracellular non-membrane-bounded organelle; 4/25; 2.1422E-03 | Lysine repeats |
| **Feature 180** |  | preribosome; 8/212; 3.3055E-05  small-subunit processome; 4/212; 2.9065E-03  phospholipid biosynthetic process; 4/212; 2.9065E-03  nucleus; 27/212; 3.3448E-03 |  |  | nucleolus; 5/35; 4.0728E-05  ribonucleoprotein complex; 6/35; 7.9973E-05  non-membrane-bounded organelle; 5/35; 9.9161E-04  intracellular non-membrane-bounded organelle; 5/35; 9.9161E-04 |  |
| **Feature 129** |  | phospholipid metabolic process; 3/44; 7.0930E-04  nucleus; 9/44; 3.4969E-03  organophosphate metabolic process; 3/44; 3.8866E-03  cellular lipid metabolic process; 3/44; 7.1956E-03 |  |  | ribonucleoprotein complex; 5/21; 6.2903E-05  hydrolase activity, acting on ester bonds; 3/21; 1.5419E-03  nucleolus; 3/21; 1.6880E-03  protein-containing complex; 6/21; 9.6290E-03 |  |
| **Feature 228** |  | RNA biosynthetic process; 4/90; 1.6860E-03  organelle localization; 3/90; 3.2163E-03  non-membrane-bounded organelle; 7/90; 3.7883E-03  intracellular non-membrane-bounded organelle; 7/90; 3.7883E-03 |  |  | nucleolus; 5/28; 1.3072E-05  intracellular non-membrane-bounded organelle; 5/28; 3.3940E-04  non-membrane-bounded organelle; 5/28; 3.3940E-04 | Lysine repeats |
| **Feature 203** |  | rRNA processing; 4/64; 2.4889E-03  rRNA metabolic process; 4/64; 3.2946E-03  nuclear protein-containing complex; 8/64; 4.1611E-03  protein-containing complex; 13/64; 6.5326E-03 |  |  | nucleolus; 7/51; 1.2458E-06  ribonucleoprotein complex; 8/51; 9.2915E-06  intracellular non-membrane-bounded organelle; 7/51; 1.1796E-04  non-membrane-bounded organelle; 7/51; 1.1796E-04 |  |
| **Feature 239** |  |  |  |  | ncRNA metabolic process; 4/29; 9.2575E-04  rRNA processing; 3/29; 2.1761E-03  rRNA metabolic process; 3/29; 2.7042E-03  RNA metabolic process; 5/29; 2.8116E-03 | Lysine repeats |
| **Feature 231** |  | DNA-binding transcription factor activity, RNA polymerase II-specific; 6/136; 4.4595E-04  DNA-binding transcription factor activity; 6/136; 1.4486E-03  process utilizing autophagic mechanism; 3/136; 5.1552E-03  autophagy; 3/136; 5.1552E-03 | PKA phosphorylation consensus site |  | nucleolus; 6/83; 3.1941E-04  ribonucleoprotein complex; 8/83; 3.3411E-04  rRNA processing; 5/83; 7.9500E-04  rRNA metabolic process; 5/83; 1.1340E-03 |  |
| **Feature 156** |  |  |  |  |  |  |
| **Feature 213** |  | nucleic acid metabolic process; 21/141; 4.2024E-06  RNA modification; 7/141; 8.6631E-06  nucleobase-containing compound metabolic process; 21/141; 1.2700E-05  cellular aromatic compound metabolic process; 21/141; 1.7324E-05 | cNLS |  |  |  |
| **Feature 100** |  | small-subunit processome; 4/138; 5.7344E-04  ribonucleoprotein complex; 10/138; 6.0562E-04  signal transduction; 6/138; 3.9092E-03  intracellular signal transduction; 5/138; 6.3013E-03 |  |  | rRNA processing; 5/66; 2.7233E-04  rRNA metabolic process; 5/66; 3.9238E-04  nucleolus; 5/66; 8.6546E-04  ncRNA processing; 5/66; 9.9647E-04 |  |
| **Feature 250** |  |  |  |  |  |  |
| **Feature 173** |  | protein serine/threonine kinase activity; 7/240; 4.7916E-03 |  |  | RNA binding; 5/20; 5.7061E-05  nucleic acid binding; 5/20; 1.4384E-03  organic cyclic compound binding; 5/20; 2.7896E-03  heterocyclic compound binding; 5/20; 2.7896E-03 | R/G repeats |
| **Feature 251** |  | nucleolus; 7/95; 8.2803E-05  nucleic acid metabolic process; 12/95; 2.4763E-03  small-subunit processome; 3/95; 2.6931E-03  nucleobase-containing compound metabolic process; 12/95; 4.3707E-03 | cNLS |  | response to chemical; 4/145; 5.8919E-03 |  |
| **Feature 29** |  |  |  |  |  |  |
| **Feature 182** |  |  |  |  | integral component of organelle membrane; 3/58; 8.4670E-03  intrinsic component of organelle membrane; 3/58; 9.6621E-03 |  |
| **Feature 112** |  |  |  |  | ncRNA metabolic process; 6/110; 6.5014E-03  positive regulation of catalytic activity; 3/110; 9.8507E-03  positive regulation of molecular function; 3/110; 9.8507E-03  nucleotidyltransferase activity; 3/110; 9.8507E-03 |  |
| **Feature 133** |  | regulation of cellular process; 23/165; 3.1186E-05  regulation of biological process; 23/165; 1.4236E-04  biological regulation; 25/165; 7.1872E-04  regulation of nitrogen compound metabolic process; 15/165; 8.4727E-04 | NLxxxL, Clb5 and Clb6 docking site. |  |  |  |
| **Feature 106** |  | transferase activity, transferring phosphorus-containing groups; 8/135; 6.3207E-03 |  |  | protein folding; 3/78; 6.6001E-04  rRNA processing; 4/78; 5.1172E-03  rRNA metabolic process; 4/78; 6.7207E-03 |  |
| **Feature 66** |  | protein modification by small protein conjugation or removal; 3/72; 5.8425E-03  mRNA binding; 3/72; 9.9178E-03 |  |  |  |  |
| **Feature 128** |  | cellular nitrogen compound biosynthetic process; 5/100; 8.0130E-03 |  |  |  |  |
| **Feature 142** |  | nucleoside-triphosphatase regulator activity; 4/125; 1.9341E-03  GTPase regulator activity; 4/125; 1.9341E-03  enzyme regulator activity; 5/125; 4.1236E-03  molecular function regulator; 5/125; 4.1236E-03 |  |  | DNA recombination; 3/94; 6.3606E-03 |  |
| **Feature 17** |  | phosphotransferase activity, alcohol group as acceptor; 6/65; 3.7303E-04  kinase activity; 6/65; 4.8068E-04  positive regulation of transcription by RNA polymerase II; 4/65; 6.1978E-04  protein kinase activity; 5/65; 1.5606E-03 |  |  |  |  |
| **Feature 44** |  | protein dephosphorylation; 3/70; 1.4188E-04  dephosphorylation; 3/70; 2.7870E-04 |  |  |  |  |
| **Feature 135** |  | transporter activity; 5/35; 1.2228E-03  transmembrane transporter activity; 4/35; 6.3084E-03 |  |  | chromatin organization; 3/25; 2.8273E-03  protein-containing complex subunit organization; 3/25; 5.2711E-03 |  |
| **Feature 58** |  | Sm-like protein family complex; 3/97; 2.8594E-03  spliceosomal snRNP complex; 3/97; 2.8594E-03  small nuclear ribonucleoprotein complex; 3/97; 2.8594E-03 |  |  |  |  |
| **Feature 223** |  | catalytic activity; 6/261; 9.8514E-05  protein modification process; 13/261; 8.5653E-03  cellular protein modification process; 13/261; 8.5653E-03  chromatin organization; 8/261; 8.6882E-03 | Gle2-binding-sequence motif |  |  |  |
| **Feature 18** |  | nucleotidyltransferase activity; 4/191; 6.5821E-03  RNA methylation; 3/191; 8.7613E-03  glycerophospholipid biosynthetic process; 3/191; 8.7613E-03 |  |  |  |  |
| **Feature 9** |  | negative regulation of macromolecule metabolic process; 4/80; 4.1577E-03  negative regulation of gene expression; 3/80; 5.1368E-03  negative regulation of metabolic process; 4/80; 6.4374E-03  positive regulation of RNA metabolic process; 4/80; 7.3505E-03 |  |  | nucleobase-containing compound biosynthetic process; 4/67; 2.5381E-03  aromatic compound biosynthetic process; 4/67; 2.9464E-03  heterocycle biosynthetic process; 4/67; 3.3976E-03  organic cyclic compound biosynthetic process; 4/67; 5.0287E-03 |  |
| **Feature 80** |  | helicase activity; 3/159; 1.6226E-03  bounding membrane of organelle; 5/159; 6.4697E-03  adenyl nucleotide binding; 3/159; 8.0080E-03  ATP binding; 3/159; 8.0080E-03 |  |  |  |  |
| **Feature 97** |  | reproductive process; 4/128; 3.7567E-03  protein phosphorylation; 4/128; 3.7567E-03  phosphorylation; 4/128; 6.1030E-03  molecular\_function; 17/128; 6.9080E-03 | acidic Phe motif |  |  |  |
| **Feature 211** |  |  | AGC docking motif |  |  |  |
| **Feature 79** |  | catalytic complex; 4/27; 6.4374E-03 |  |  |  |  |
| **Feature 143** |  | protein targeting; 3/117; 4.8831E-03  DNA repair; 4/117; 5.5035E-03  primary metabolic process; 23/117; 6.2322E-03  metabolic process; 25/117; 7.1319E-03 |  |  |  |  |
| **Feature 229** |  | chromatin remodeling; 4/170; 4.3234E-03  cellular\_component; 60/170; 5.3665E-03  cellular process; 58/170; 5.9891E-03  cellular component assembly; 8/170; 6.1810E-03 |  |  |  | acidic repeats |
| **Feature 234** |  |  |  |  |  |  |
| **Feature 220** |  | nucleic acid phosphodiester bond hydrolysis; 3/53; 3.2964E-04 |  |  |  |  |
| **Feature 125** |  | protein ubiquitination; 4/130; 1.1100E-03  protein modification by small protein conjugation; 4/130; 1.1100E-03  protein-containing complex; 3/130; 2.7487E-03  cellular\_component; 43/130; 3.7673E-03 | FFAT-like motif |  |  |  |
| **Feature 202** |  | cellular response to nutrient levels; 4/154; 5.0624E-04  response to nutrient levels; 4/154; 5.0624E-04  response to external stimulus; 4/154; 5.0624E-04  cellular response to extracellular stimulus; 4/154; 5.0624E-04 |  |  | mannosyltransferase complex; 3/60; 9.1946E-06  mannan polymerase complex; 3/60; 9.1946E-06  carbohydrate derivative biosynthetic process; 3/60; 4.7701E-04  carbohydrate derivative metabolic process; 3/60; 1.3427E-03 |  |
| **Feature 196** |  |  |  |  |  |  |
| **Feature 160** |  | negative regulation of RNA biosynthetic process; 3/107; 5.2618E-03  negative regulation of nucleic acid-templated transcription; 3/107; 5.2618E-03  negative regulation of RNA metabolic process; 3/107; 5.2618E-03  negative regulation of transcription, DNA-templated; 3/107; 5.2618E-03 |  |  |  |  |
| **Feature 110** |  | cellular anatomical entity; 28/122; 2.6318E-03  regulation of GTPase activity; 3/122; 3.7836E-03  cellular\_component; 41/122; 6.8825E-03  macromolecule modification; 9/122; 9.4147E-03 |  |  |  |  |
| **Feature 209** |  | cellular component organization or biogenesis; 7/34; 2.8171E-03  cellular\_component; 7/34; 2.9364E-03  biological\_process; 19/34; 4.3439E-03  cellular process; 16/34; 4.7831E-03 |  |  | cell wall; 4/44; 2.3421E-05  external encapsulating structure; 4/44; 2.3421E-05  fungal-type cell wall; 3/44; 3.9545E-04  active ion transmembrane transporter activity; 3/44; 1.7282E-03 |  |
| **Feature 212** |  | regulation of biological process; 18/139; 1.8050E-03  cytosol; 7/139; 6.5010E-03  regulation of cellular process; 16/139; 6.7786E-03  regulation of transcription by RNA polymerase II; 8/139; 6.7796E-03 |  |  | cytoplasm; 11/65; 2.3897E-03 |  |
| **Feature 208** |  | ATP-dependent chromatin remodeling; 3/246; 2.4636E-03  intrinsic component of plasma membrane; 3/246; 2.4636E-03  negative regulation of cellular protein metabolic process; 3/246; 5.7619E-03  negative regulation of protein metabolic process; 3/246; 5.7619E-03 |  |  |  |  |
| **Feature 69** |  | glycerophospholipid biosynthetic process; 3/123; 2.4999E-03  cellular component organization or biogenesis; 15/123; 4.9293E-03  small-subunit processome; 3/123; 5.6253E-03  phospholipid biosynthetic process; 3/123; 5.6253E-03 |  |  | nucleolus; 4/26; 1.9475E-04  intracellular non-membrane-bounded organelle; 4/26; 2.4889E-03  non-membrane-bounded organelle; 4/26; 2.4889E-03 | acidic repeats |
| **Feature 82** |  | protein-DNA complex subunit organization; 3/111; 7.7960E-03 |  |  |  |  |
| **Feature 89** |  | macromolecule modification; 8/74; 1.3550E-03  chromatin organization; 5/74; 1.4591E-03  cellular process; 31/74; 1.5029E-03  protein modification by small protein conjugation; 3/74; 2.4679E-03 | CKII consensus phosphorylation site |  | CKII consensus phosphorylation sites |  |
| **Feature 16** |  | mitotic cell cycle process; 4/93; 3.5942E-03 |  |  |  | acidic repeat |
| **Feature 5** |  | cellular response to starvation; 3/266; 7.2088E-03  response to starvation; 3/266; 7.2088E-03 |  |  |  | acidic repeat |
| **Feature 21** |  | activation of GTPase activity; 3/143; 4.9373E-04  positive regulation of GTPase activity; 3/143; 4.9373E-04  regulation of catalytic activity; 7/143; 5.7007E-04  regulation of molecular function; 7/143; 5.7007E-04 |  |  |  | acidic repeat |
| **Feature 74** |  |  |  |  | structural constituent of ribosome; 3/31; 3.2052E-04  structural molecule activity; 3/31; 1.8420E-03 | acidic repeat |
| **Feature 81** |  | nucleolus; 13/392; 2.4514E-04  nucleic acid metabolic process; 36/392; 2.4601E-04  rRNA metabolic process; 11/392; 7.6310E-04  nucleobase-containing compound metabolic process; 36/392; 9.9049E-04 |  |  |  | acidic repeat |
| **Feature 88** |  | nucleolus; 12/251; 1.2516E-05  nucleic acid metabolic process; 27/251; 1.0334E-04  rRNA processing; 9/251; 2.4727E-04  nucleobase-containing compound metabolic process; 27/251; 3.4290E-04 |  |  |  | acidic repeat |
| **Feature 151** |  | histone binding; 5/154; 4.8281E-05  rRNA processing; 6/154; 2.2379E-03  rRNA metabolic process; 6/154; 3.3440E-03  RNA processing; 8/154; 5.0079E-03 |  |  |  | acidic repeat |
| **Feature 205** |  | small-subunit processome; 3/104; 3.4917E-03 |  |  |  |  |
| **Feature 164** |  | rRNA processing; 9/116; 4.3315E-07  rRNA metabolic process; 9/116; 8.9301E-07  ncRNA processing; 9/116; 5.5020E-06  ncRNA metabolic process; 9/116; 5.2498E-05 | CKII consensus phosphorylation site |  | cytoplasm; 6/29; 8.8766E-03 |  |
| **Feature 254** |  | preribosome; 7/253; 8.5068E-04  small-subunit processome; 4/253; 5.5689E-03  protein modification by small protein conjugation or removal; 5/253; 7.9467E-03 |  |  | small-subunit processome; 3/75; 1.3572E-03  cytosol; 5/75; 6.8412E-03  protein-containing complex; 14/75; 8.0286E-03 |  |
| **Feature 244** |  | negative regulation of metabolic process; 4/23; 5.2396E-05  negative regulation of biological process; 4/23; 2.3008E-04  negative regulation of cellular biosynthetic process; 3/23; 4.2333E-04  negative regulation of biosynthetic process; 3/23; 4.2333E-04 |  |  |  |  |
| **Feature 183** |  | DNA metabolic process; 7/153; 5.7278E-03 |  |  | cytosol; 3/21; 4.4122E-03 |  |
| **Feature 236** |  | chromosome organization; 9/350; 1.8807E-04  negative regulation of macromolecule metabolic process; 9/350; 1.5041E-03  establishment or maintenance of cell polarity; 3/350; 1.9036E-03  internal peptidyl-lysine acetylation; 4/350; 2.8635E-03 | Polyphosphorylation site (D/E/K repeat) |  | nucleolus; 3/20; 1.4584E-03  intracellular non-membrane-bounded organelle; 3/20; 9.6767E-03  non-membrane-bounded organelle; 3/20; 9.6767E-03 | Polyphosphorylation site (D/E/K repeat) |
| **Feature 240** |  |  |  |  | RNA polymerase complex; 3/48; 6.9637E-04  DNA-directed RNA polymerase complex; 3/48; 6.9637E-04  nuclear DNA-directed RNA polymerase complex; 3/48; 6.9637E-04  transferase complex, transferring phosphorus-containing groups; 3/48; 4.3192E-03 |  |
| **Feature 185** |  | protein ubiquitination; 3/104; 6.4981E-03  protein modification by small protein conjugation; 3/104; 6.4981E-03  GTPase activator activity; 3/104; 8.4339E-03 |  |  | cellular process; 22/52; 5.6036E-03 |  |
| **Feature 126** |  | Golgi apparatus; 3/27; 4.2622E-05 |  |  |  |  |
| **Feature 204** |  | intracellular protein-containing complex; 9/229; 2.6630E-03  negative regulation of cellular amide metabolic process; 3/229; 4.6887E-03  TOR complex; 3/229; 8.8170E-03 |  |  |  |  |
| **Feature 154** |  |  |  |  | fungal-type cell wall; 5/161; 1.1438E-04  cell wall; 5/161; 3.2721E-04  external encapsulating structure; 5/161; 3.2721E-04  cell wall organization; 5/161; 7.5303E-04 |  |
| **Feature 95** |  | DNA-binding transcription factor activity, RNA polymerase II-specific; 5/59; 6.8060E-05  transcription regulator activity; 6/59; 1.9054E-04  DNA-binding transcription factor activity; 5/59; 1.9253E-04 |  |  |  |  |
| **Feature 252** |  | biological regulation; 18/71; 3.3293E-06  regulation of cellular process; 15/71; 5.6366E-06  regulation of biological process; 15/71; 1.6070E-05  regulation of hydrolase activity; 4/71; 2.2323E-04 | CDK consensus phosphorylation site |  | membrane organization; 3/61; 2.3680E-03  organelle organization; 6/61; 3.0926E-03 |  |
| **Feature 191** |  | positive regulation of biological process; 18/491; 1.9066E-04  positive regulation of cellular process; 18/491; 1.9066E-04  regulation of cellular process; 45/491; 5.0392E-04  positive regulation of nucleobase-containing compound metabolic process; 13/491; 7.8300E-04 |  |  | cell surface; 3/40; 5.1539E-05 |  |
| **Feature 237** |  | preribosome; 4/63; 6.8466E-04  small-subunit processome; 3/63; 8.1381E-04  ribonucleoprotein complex; 6/63; 2.0882E-03  ncRNA processing; 4/63; 6.3718E-03 | CKII consensus phosphorylation site |  |  |  |
| **Feature 136** |  | anchored component of membrane; 3/164; 1.7767E-03  cell wall; 4/164; 3.7928E-03  external encapsulating structure; 4/164; 3.7928E-03  protein-containing complex; 6/164; 7.8485E-03 |  |  | fungal-type cell wall; 5/31; 2.7965E-08  external encapsulating structure; 5/31; 8.6540E-08  cell wall; 5/31; 8.6540E-08  cell surface; 3/31; 2.3619E-05 | S/T repeat |
| **Feature 224** |  | endocytosis; 4/249; 5.2546E-03  basal RNA polymerase II transcription machinery binding; 3/249; 5.9660E-03  basal transcription machinery binding; 3/249; 5.9660E-03  DNA-binding transcription factor activity; 7/249; 7.3544E-03 |  |  |  |  |
| **Feature 232** |  | positive regulation of cellular catabolic process; 4/446; 2.7157E-03  positive regulation of catabolic process; 4/446; 2.7157E-03  establishment or maintenance of cell polarity; 3/446; 3.9462E-03  nuclear-transcribed mRNA catabolic process, deadenylation-dependent decay; 3/446; 3.9462E-03 |  |  | plasma membrane; 4/20; 4.6562E-04  protein binding; 3/20; 8.0931E-03 |  |
