# Supplementary material for: Discovering molecular features of intrinsically disordered regions by using evolution for contrastive learning
Source: PLoS Comput Biol. 2022 Jun 29;18(6):e1010238. doi: 10.1371/journal.pcbi.1010238 (PMC9275697; doi:10.1371/journal.pcbi.1010238)
Supplement: S5 File — (TAR.GZ) [file pcbi.1010238.s009.tar.gz › s5/human_table.html]

| Feature | Max Logo | Max Enrichments | Max Annotation | Average Logo | Average Enrichments | Average Annotation |
| --- | --- | --- | --- | --- | --- | --- |
| **Feature 78** |  |  |  |  |  |  |
| **Feature 52** |  | enzyme regulator activity; 7/241; 3.2342E-03 cellular anatomical entity; 108/241; 6.7883E-03 biological\_process; 53/241; 6.7944E-03 external encapsulating structure; 6/241; 8.4596E-03 |  |  | catalytic complex; 3/20; 4.3691E-03 nuclear protein-containing complex; 3/20; 6.2342E-03 |  |
| **Feature 88** |  | regulation of mitotic cell cycle; 5/300; 1.8191E-04 ATPase complex; 3/300; 9.3623E-04 SWI/SNF superfamily-type complex; 3/300; 9.3623E-04 protein kinase binding; 5/300; 1.0478E-03 |  |  |  |  |
| **Feature 17** |  | integral component of membrane; 7/46; 5.9253E-03 intrinsic component of membrane; 7/46; 6.5347E-03 |  |  | carbohydrate derivative biosynthetic process; 3/20; 2.4917E-05 carbohydrate derivative metabolic process; 3/20; 1.3006E-04 cellular lipid metabolic process; 3/20; 1.9242E-04 lipid metabolic process; 3/20; 2.7152E-04 |  |
| **Feature 0** |  | organelle localization; 3/157; 3.3261E-03 positive regulation of transcription by RNA polymerase II; 4/157; 4.0037E-03 positive regulation of transcription, DNA-templated; 4/157; 9.5293E-03 positive regulation of RNA biosynthetic process; 4/157; 9.5293E-03 |  |  | transferase complex; 3/20; 1.4007E-03 catalytic complex; 3/20; 4.3691E-03 |  |
| **Feature 31** |  | macromolecule glycosylation; 4/274; 7.8755E-04 protein glycosylation; 4/274; 7.8755E-04 nuclear protein-containing complex; 14/274; 8.1660E-04 glycosylation; 4/274; 1.7445E-03 |  |  | organonitrogen compound metabolic process; 6/43; 1.8091E-03 protein metabolic process; 5/43; 3.7578E-03 integral component of membrane; 7/43; 4.0369E-03 intrinsic component of membrane; 7/43; 4.4622E-03 |  |
| **Feature 206** |  | axon guidance; 3/143; 3.0287E-04 neuron projection guidance; 3/143; 3.0287E-04 movement of cell or subcellular component; 5/143; 6.8431E-04 cellular localization; 8/143; 7.7247E-04 |  |  | microtubule organizing center; 4/20; 3.9558E-06 |  |
| **Feature 136** |  | microtubule organizing center; 3/26; 3.9045E-04 |  |  | cytoskeleton; 3/27; 2.3386E-04 |  |
| **Feature 69** |  | voltage-gated potassium channel activity; 5/647; 2.7651E-03 DNA-binding transcription factor activity; 6/647; 3.2244E-03 delayed rectifier potassium channel activity; 3/647; 3.2682E-03 transcription regulator activity; 8/647; 4.1558E-03 |  |  |  |  |
| **Feature 225** |  | cell-cell junction organization; 3/303; 3.5824E-04 cell junction organization; 4/303; 7.1137E-04 molecular function regulator; 11/303; 1.1920E-03 signaling receptor activator activity; 5/303; 1.2779E-03 |  |  |  |  |
| **Feature 237** |  |  |  |  | organelle; 7/21; 8.4309E-03 |  |
| **Feature 4** |  | chromatin binding; 5/259; 1.4480E-03 intrinsic component of membrane; 3/259; 1.9292E-03 integral component of membrane; 3/259; 2.7899E-03 Sm-like protein family complex; 3/259; 4.4000E-03 |  |  | membrane protein complex; 3/20; 2.1650E-03 |  |
| **Feature 63** |  | supramolecular fiber organization; 6/312; 7.6035E-05 voltage-gated sodium channel complex; 3/312; 6.6945E-04 sodium channel complex; 3/312; 1.0493E-03 enzyme regulator activity; 8/312; 3.5732E-03 |  |  |  |  |
| **Feature 157** |  | glycerophospholipid metabolic process; 3/298; 9.3528E-03 |  |  |  |  |
| **Feature 2** |  | negative regulation of cellular metabolic process; 6/113; 5.6582E-04 negative regulation of metabolic process; 6/113; 9.2246E-04 positive regulation of nitrogen compound metabolic process; 5/113; 4.1402E-03 negative regulation of cellular process; 7/113; 4.3115E-03 |  |  | intracellular protein-containing complex; 3/36; 5.1856E-03 transferase complex; 3/36; 7.6917E-03 |  |
| **Feature 43** |  |  |  |  | intracellular organelle; 8/27; 4.4174E-03 organelle organization; 3/27; 8.8820E-03 |  |
| **Feature 20** |  | nucleoplasm; 9/246; 2.1581E-05 regulation of cation transmembrane transport; 3/246; 1.4723E-03 nuclear protein-containing complex; 12/246; 2.8005E-03 regulation of ion transmembrane transport; 3/246; 4.6112E-03 |  |  |  |  |
| **Feature 18** |  | nucleolus; 7/249; 2.6539E-04 intracellular non-membrane-bounded organelle; 11/249; 3.4777E-03 non-membrane-bounded organelle; 11/249; 3.4777E-03 nuclear export; 3/249; 4.7703E-03 |  |  | protein kinase binding; 4/20; 1.8893E-07 kinase binding; 4/20; 2.1668E-07 enzyme binding; 4/20; 9.7986E-06 macromolecule metabolic process; 4/20; 9.4159E-03 |  |
| **Feature 34** |  | DNA repair; 3/155; 5.7958E-03 cytoskeletal protein binding; 4/155; 6.2991E-03 |  |  | protein kinase binding; 4/39; 3.0972E-06 kinase binding; 4/39; 3.5472E-06 enzyme binding; 4/39; 1.5041E-04 RNA binding; 4/39; 8.1524E-04 |  |
| **Feature 72** |  | nuclear protein-containing complex; 18/382; 4.0181E-04 membrane; 6/382; 9.6034E-04 ATPase complex; 3/382; 1.8843E-03 SWI/SNF superfamily-type complex; 3/382; 1.8843E-03 |  |  | protein kinase binding; 4/20; 1.8893E-07 kinase binding; 4/20; 2.1668E-07 enzyme binding; 4/20; 9.7986E-06 |  |
| **Feature 59** |  | integrator complex; 5/287; 1.1760E-06 nuclear protein-containing complex; 19/287; 2.5981E-06 snRNA metabolic process; 4/287; 4.6207E-05 nucleolus; 8/287; 9.9091E-05 |  |  |  |  |
| **Feature 30** |  |  |  |  | intracellular organelle; 9/36; 8.7933E-03 |  |
| **Feature 84** |  | organelle; 32/156; 1.6784E-03 nucleus; 17/156; 3.3189E-03 plasma membrane bounded cell projection; 6/156; 4.4969E-03 cell projection; 6/156; 6.0313E-03 |  |  | protein kinase binding; 4/32; 1.3716E-06 kinase binding; 4/32; 1.5717E-06 enzyme binding; 4/32; 6.8244E-05 nucleolus; 3/32; 5.7061E-04 |  |
| **Feature 181** |  | transcription cis-regulatory region binding; 9/115; 2.0798E-03 transcription regulatory region nucleic acid binding; 9/115; 2.0798E-03 RNA polymerase II cis-regulatory region sequence-specific DNA binding; 8/115; 2.3010E-03 cis-regulatory region sequence-specific DNA binding; 8/115; 2.3010E-03 |  |  |  |  |
| **Feature 67** |  | RNA metabolic process; 15/286; 1.6903E-06 RNA processing; 13/286; 1.8331E-06 nucleic acid metabolic process; 17/286; 4.0859E-06 ribonucleoprotein complex; 13/286; 1.3394E-05 |  |  |  |  |
| **Feature 12** |  | voltage-gated sodium channel complex; 3/249; 3.4527E-04 sodium channel complex; 3/249; 5.4346E-04 monovalent inorganic cation homeostasis; 3/249; 1.1271E-03 microtubule-based process; 5/249; 8.1853E-03 |  |  | ribonucleoprotein complex; 3/23; 1.8943E-03 |  |
| **Feature 27** |  | RNA binding; 12/250; 1.7066E-05 keratinization; 3/250; 1.5423E-03 ribonucleoprotein complex; 9/250; 1.6034E-03 nuclear protein-containing complex; 11/250; 8.8303E-03 |  |  |  |  |
| **Feature 81** |  | nucleolus; 12/400; 6.6115E-07 RNA binding; 17/400; 1.4375E-06 cell adhesion; 11/400; 3.3377E-06 biological adhesion; 11/400; 3.3377E-06 |  |  |  |  |
| **Feature 101** |  | nuclear protein-containing complex; 28/421; 8.3845E-09 integrator complex; 5/421; 7.7574E-06 protein-containing complex; 52/421; 2.9021E-05 RNA processing; 12/421; 4.2964E-04 |  |  |  |  |
| **Feature 189** |  | ncRNA processing; 3/54; 2.5067E-03 ncRNA metabolic process; 3/54; 3.0422E-03 cellular component organization; 6/54; 9.4892E-03 cellular component organization or biogenesis; 6/54; 9.9582E-03 |  |  | organelle; 8/21; 1.8885E-03 |  |
| **Feature 250** |  | nuclear protein-containing complex; 20/397; 7.7370E-05 centriolar satellite; 4/397; 5.9074E-04 cytoplasmic microtubule; 3/397; 7.9241E-04 histone acetyltransferase complex; 4/397; 3.0900E-03 |  |  | protein-containing complex; 6/21; 2.2402E-03 nuclear protein-containing complex; 3/21; 7.1716E-03 |  |
| **Feature 123** |  | regulation of gene expression, epigenetic; 4/380; 1.2532E-06 HCN channel complex; 3/380; 3.7599E-05 regulation of gene silencing; 3/380; 3.7599E-05 modification-dependent protein binding; 4/380; 3.4265E-04 |  |  | nucleosome organization; 5/19; 1.9493E-10 protein-DNA complex subunit organization; 5/19; 2.4882E-10 nucleosome assembly; 4/19; 2.1668E-08 protein-DNA complex assembly; 4/19; 2.7057E-08 |  |
| **Feature 142** |  | nuclear protein-containing complex; 16/269; 6.0735E-05 methylation-dependent protein binding; 3/269; 1.2830E-04 methylated histone binding; 3/269; 1.2830E-04 DNA helicase activity; 3/269; 2.5208E-04 |  |  | nucleosome organization; 8/19; 3.5947E-18 protein-DNA complex subunit organization; 8/19; 5.5071E-18 nucleosome assembly; 7/19; 5.3424E-16 protein-DNA complex assembly; 7/19; 8.2114E-16 |  |
| **Feature 26** |  | ribonucleoprotein complex binding; 3/272; 4.4774E-04 nucleolus; 7/272; 4.5294E-04 histone deacetylase complex; 3/272; 1.0368E-03 microtubule motor activity; 3/272; 1.0368E-03 |  |  | nucleosome organization; 5/20; 2.5958E-10 protein-DNA complex subunit organization; 5/20; 3.3132E-10 nucleosome assembly; 4/20; 2.7057E-08 protein-DNA complex assembly; 4/20; 3.3783E-08 |  |
| **Feature 135** |  |  |  |  | nucleosome organization; 9/41; 2.0203E-17 protein-DNA complex subunit organization; 9/41; 3.3106E-17 chromatin organization; 10/41; 1.1220E-14 nucleosome assembly; 7/41; 2.3353E-13 |  |
| **Feature 144** |  | nucleolus; 12/392; 5.3320E-07 RNA metabolic process; 17/392; 4.4650E-06 RNA binding; 16/392; 5.0637E-06 rRNA processing; 8/392; 7.5704E-06 |  |  |  |  |
| **Feature 6** |  | cell cycle process; 8/256; 9.1140E-05 organelle organization; 13/256; 2.3765E-04 microtubule-based process; 7/256; 2.8159E-04 cellular component organization; 19/256; 1.2630E-03 |  |  |  |  |
| **Feature 60** |  | RNA binding; 7/162; 1.9196E-03 |  |  | macromolecule metabolic process; 8/69; 9.1718E-03 |  |
| **Feature 25** |  | intracellular non-membrane-bounded organelle; 18/301; 3.5463E-06 non-membrane-bounded organelle; 18/301; 3.5463E-06 nucleolus; 9/301; 2.0063E-05 intracellular organelle; 50/301; 4.9027E-04 |  |  | nucleosome organization; 8/83; 1.7479E-12 protein-DNA complex subunit organization; 8/83; 2.6643E-12 nucleosome assembly; 7/83; 4.1473E-11 protein-DNA complex assembly; 7/83; 6.3429E-11 |  |
| **Feature 116** |  | plasma membrane bounded cell projection; 5/107; 4.1492E-03 cell projection; 5/107; 5.3633E-03 cilium; 3/107; 8.2214E-03 |  |  |  |  |
| **Feature 13** |  | reproductive process; 7/310; 1.2047E-03 ciliary basal body; 4/310; 1.5470E-03 microtubule organizing center; 7/310; 1.6094E-03 |  |  | membrane-bounded organelle; 7/19; 1.7910E-03 organelle; 7/19; 4.5187E-03 |  |
| **Feature 149** |  | microtubule-based process; 7/267; 3.6345E-04 nucleoplasm; 7/267; 1.3818E-03 microtubule-based movement; 4/267; 1.5862E-03 microtubule binding; 4/267; 2.2204E-03 |  |  | membrane protein complex; 3/20; 2.1650E-03 catalytic complex; 3/20; 4.3691E-03 organelle; 7/20; 6.2501E-03 protein-containing complex; 5/20; 9.6730E-03 |  |
| **Feature 19** |  |  |  |  |  |  |
| **Feature 11** |  | integral component of membrane; 23/136; 1.1479E-07 intrinsic component of membrane; 23/136; 1.5946E-07 intracellular organelle; 3/136; 5.2601E-04 defense response; 3/136; 1.9283E-03 |  |  |  |  |
| **Feature 22** |  | collagen trimer; 3/130; 6.0964E-04 response to chemical; 4/130; 1.7222E-03 extracellular space; 6/130; 5.3034E-03 response to organic substance; 3/130; 7.4186E-03 |  |  |  |  |
| **Feature 70** |  | regulation of cell-cell adhesion; 3/131; 8.1427E-05 response to external stimulus; 5/131; 1.6244E-04 intrinsic component of membrane; 17/131; 2.1186E-04 regulation of cell adhesion; 3/131; 2.3388E-04 |  |  |  |  |
| **Feature 23** |  | integral component of membrane; 11/83; 1.9682E-03 intrinsic component of membrane; 11/83; 2.2712E-03 external encapsulating structure; 4/83; 3.1879E-03 extracellular matrix; 4/83; 3.1879E-03 |  |  |  |  |
| **Feature 41** |  | integral component of membrane; 45/315; 3.6579E-11 intrinsic component of membrane; 45/315; 6.7323E-11 cellular anatomical entity; 154/315; 5.7130E-06 extracellular space; 14/315; 3.2144E-05 |  |  | response to external biotic stimulus; 3/20; 1.8605E-05 response to biotic stimulus; 3/20; 1.8605E-05 response to external stimulus; 3/20; 6.3934E-05 response to stimulus; 3/20; 2.3785E-03 |  |
| **Feature 244** |  | integral component of membrane; 11/70; 4.6264E-04 intrinsic component of membrane; 11/70; 5.3945E-04 catalytic activity; 10/70; 3.4730E-03 glycosyltransferase activity; 3/70; 4.2588E-03 |  |  | extracellular region; 3/41; 1.3097E-03 signaling receptor binding; 3/41; 5.4448E-03 |  |
| **Feature 255** |  | extracellular matrix; 11/194; 1.5619E-07 external encapsulating structure; 11/194; 1.5619E-07 integral component of membrane; 25/194; 6.1394E-06 Golgi apparatus; 8/194; 7.7153E-06 |  |  | extracellular region; 4/39; 5.1166E-05 hydrolase activity; 4/39; 5.3331E-03 |  |
| **Feature 164** |  | response to external biotic stimulus; 3/42; 1.8012E-04 response to biotic stimulus; 3/42; 1.8012E-04 response to external stimulus; 3/42; 6.0564E-04 Golgi apparatus; 3/42; 1.4052E-03 |  |  |  |  |
| **Feature 187** |  | integral component of membrane; 30/168; 3.4225E-10 intrinsic component of membrane; 30/168; 5.3085E-10 hydrolase activity; 13/168; 1.1779E-05 catalytic activity; 23/168; 2.3503E-05 |  |  |  |  |
| **Feature 125** |  | mRNA export from nucleus; 3/378; 6.8638E-04 mRNA transport; 3/378; 6.8638E-04 response to stimulus; 13/378; 1.9505E-03 dynein complex; 3/378; 2.6744E-03 |  |  | response to external biotic stimulus; 3/36; 1.1324E-04 response to biotic stimulus; 3/36; 1.1324E-04 response to external stimulus; 3/36; 3.8301E-04 extracellular region; 3/36; 8.9390E-04 |  |
| **Feature 153** |  | positive regulation of signaling; 7/260; 4.2632E-04 positive regulation of cell communication; 7/260; 4.2632E-04 monooxygenase activity; 3/260; 6.1675E-04 integral component of membrane; 25/260; 9.7932E-04 |  |  |  |  |
| **Feature 183** |  | keratin filament; 6/176; 1.6293E-04 intermediate filament; 6/176; 1.0047E-03 nuclear export; 3/176; 1.7852E-03 reproductive process; 5/176; 2.3547E-03 |  |  | keratin filament; 3/20; 1.0460E-04 intermediate filament; 3/20; 2.8268E-04 polymeric cytoskeletal fiber; 3/20; 5.1970E-04 supramolecular polymer; 3/20; 7.0492E-04 |  |
| **Feature 47** |  | regulation of catalytic activity; 3/60; 9.0243E-03 |  |  | catalytic activity; 10/36; 1.1159E-05 transferase activity; 6/36; 2.4510E-04 kinase activity; 3/36; 4.3225E-03 transferase activity, transferring phosphorus-containing groups; 3/36; 5.8614E-03 |  |
| **Feature 44** |  | ciliary transition zone; 3/67; 1.0883E-05 integral component of membrane; 9/67; 4.5045E-03 intrinsic component of membrane; 9/67; 5.0721E-03 |  |  | mitochondrion; 3/20; 8.3392E-04 |  |
| **Feature 245** |  |  |  |  |  |  |
| **Feature 109** |  | Golgi organization; 3/220; 1.0663E-03 endocytosis; 3/220; 1.4014E-03 synaptic signaling; 3/220; 2.2531E-03 cell-cell signaling; 3/220; 4.7695E-03 |  |  |  |  |
| **Feature 155** |  | establishment of organelle localization; 3/140; 3.7594E-04 enzyme binding; 6/140; 4.2712E-04 organelle localization; 3/140; 2.4037E-03 |  |  | integral component of membrane; 5/24; 4.9715E-03 intrinsic component of membrane; 5/24; 5.3712E-03 |  |
| **Feature 113** |  | centrosome; 4/140; 7.8070E-04 preribosome; 3/140; 1.5540E-03 microtubule organizing center; 4/140; 7.6094E-03 protein ubiquitination; 3/140; 9.8258E-03 |  |  |  |  |
| **Feature 145** |  | secretory granule membrane; 3/135; 6.8059E-04 regulation of response to stimulus; 7/135; 3.5921E-03 cytoplasmic vesicle membrane; 4/135; 4.6970E-03 vesicle membrane; 4/135; 4.9990E-03 |  |  |  |  |
| **Feature 163** |  | single-stranded RNA binding; 3/198; 2.7709E-04 cytoskeleton organization; 6/198; 7.2487E-04 organelle organization; 10/198; 1.3005E-03 microtubule cytoskeleton organization; 4/198; 1.9774E-03 |  |  |  |  |
| **Feature 162** |  | protein kinase binding; 5/140; 3.0002E-05 kinase binding; 5/140; 3.5418E-05 microtubule binding; 4/140; 1.9687E-04 tubulin binding; 4/140; 2.7172E-04 |  |  | glycosyltransferase activity; 3/26; 2.3387E-04 lipid metabolic process; 3/26; 6.0229E-04 metabolic process; 6/26; 3.3965E-03 organonitrogen compound metabolic process; 4/26; 7.4695E-03 |  |
| **Feature 186** |  | glycosylation; 5/196; 3.0719E-05 macromolecule glycosylation; 3/196; 3.4484E-03 protein glycosylation; 3/196; 3.4484E-03 binding; 10/196; 4.1744E-03 |  |  | metabolic process; 10/33; 1.2542E-05 primary metabolic process; 9/33; 2.8868E-05 cellular metabolic process; 9/33; 4.5843E-05 organic substance metabolic process; 9/33; 4.8851E-05 |  |
| **Feature 71** |  | motor activity; 3/235; 9.5301E-04 catalytic activity; 3/235; 2.7210E-03 system development; 3/235; 6.7036E-03 cell-cell adhesion; 3/235; 7.7673E-03 |  |  |  |  |
| **Feature 193** |  | microtubule organizing center; 7/193; 9.3014E-05 centrosome; 4/193; 2.5507E-03 organelle organization; 9/193; 3.8449E-03 intracellular non-membrane-bounded organelle; 9/193; 5.6426E-03 |  |  | receptor complex; 3/29; 9.3785E-05 |  |
| **Feature 32** |  | extracellular exosome; 10/87; 3.6781E-08 extracellular vesicle; 10/87; 4.6171E-08 extracellular organelle; 10/87; 4.6171E-08 extracellular membrane-bounded organelle; 10/87; 4.6171E-08 |  |  | extracellular exosome; 8/18; 7.0643E-12 extracellular membrane-bounded organelle; 8/18; 8.5666E-12 extracellular organelle; 8/18; 8.5666E-12 extracellular vesicle; 8/18; 8.5666E-12 |  |
| **Feature 182** |  | regulation of GTPase activity; 3/134; 3.4604E-03 spliceosomal complex; 3/134; 6.2692E-03 plasma membrane protein complex; 4/134; 6.9062E-03 |  |  |  |  |
| **Feature 39** |  |  |  |  | ribonucleoprotein complex; 3/20; 1.2487E-03 |  |
| **Feature 8** |  | multicellular organism development; 3/180; 4.3771E-04 protein domain specific binding; 3/180; 1.5651E-03 multicellular organismal process; 5/180; 4.7639E-03 |  |  |  |  |
| **Feature 105** |  | Golgi organization; 3/178; 5.7572E-04 collagen trimer; 3/178; 1.5157E-03 organelle organization; 9/178; 2.2383E-03 organelle assembly; 4/178; 2.6085E-03 |  |  |  |  |
| **Feature 74** |  | regulation of signaling receptor activity; 3/296; 1.7049E-04 phosphatidylinositol binding; 4/296; 2.3138E-03 phosphatidylinositol phosphate binding; 3/296; 3.2723E-03 chromatin assembly or disassembly; 3/296; 4.1723E-03 |  |  | regulation of protein phosphorylation; 3/84; 2.1769E-03 regulation of protein metabolic process; 4/84; 3.1862E-03 regulation of phosphorylation; 3/84; 4.1989E-03 regulation of protein modification process; 3/84; 4.7618E-03 |  |
| **Feature 77** |  | cellular process; 99/411; 6.2279E-05 helicase activity; 5/411; 9.4199E-05 DNA helicase activity; 3/411; 8.7696E-04 vesicle-mediated transport; 9/411; 2.0015E-03 |  |  |  |  |
| **Feature 156** |  | positive regulation of phosphorus metabolic process; 5/323; 1.0522E-03 positive regulation of phosphate metabolic process; 5/323; 1.0522E-03 positive regulation of phosphorylation; 5/323; 1.0522E-03 chromosome organization; 5/323; 1.9639E-03 |  |  |  |  |
| **Feature 75** |  | site of polarized growth; 3/305; 7.6060E-05 nucleoplasm; 7/305; 2.9356E-03 |  |  |  |  |
| **Feature 174** |  | cellular aromatic compound metabolic process; 8/96; 4.4350E-04 heterocycle metabolic process; 8/96; 4.7196E-04 organic cyclic compound metabolic process; 8/96; 5.6637E-04 nucleic acid metabolic process; 7/96; 9.4626E-04 |  |  |  |  |
| **Feature 242** |  | cellular metabolic process; 16/116; 5.1073E-04 metabolic process; 16/116; 1.2187E-03 macromolecule metabolic process; 13/116; 1.3953E-03 RNA metabolic process; 6/116; 2.7204E-03 |  |  |  |  |
| **Feature 51** |  | nuclear protein-containing complex; 11/192; 1.1819E-03 cell-cell adhesion; 3/192; 4.4335E-03 nucleoplasm; 5/192; 7.0385E-03 nucleus; 19/192; 7.5374E-03 |  |  | cellular component assembly; 3/24; 2.0984E-03 |  |
| **Feature 223** |  | nuclear protein-containing complex; 12/178; 1.6161E-04 positive regulation of MAPK cascade; 3/178; 7.5876E-04 regulation of MAPK cascade; 3/178; 1.2266E-03 positive regulation of intracellular signal transduction; 4/178; 1.5096E-03 |  |  | hydrolase activity; 3/20; 5.3958E-03 |  |
| **Feature 65** |  | cell junction organization; 3/207; 2.8350E-03 enzyme binding; 6/207; 3.1987E-03 microtubule-based process; 5/207; 3.7807E-03 cellular response to stress; 5/207; 6.6327E-03 |  |  |  |  |
| **Feature 103** |  | protein-containing complex; 50/429; 1.8679E-04 nuclear protein-containing complex; 19/429; 8.0627E-04 chromosome segregation; 3/429; 1.6906E-03 chromatin binding; 6/429; 2.4571E-03 |  |  |  |  |
| **Feature 33** |  | catalytic activity; 8/461; 1.6144E-04 inward rectifier potassium channel activity; 3/461; 2.6074E-04 ligand-gated cation channel activity; 5/461; 7.9497E-04 microtubule organizing center; 9/461; 9.5629E-04 |  |  |  |  |
| **Feature 89** |  | nucleosome assembly; 9/292; 3.3104E-10 protein-DNA complex assembly; 9/292; 5.8836E-10 nucleosome organization; 9/292; 1.6650E-09 protein-DNA complex subunit organization; 9/292; 2.6738E-09 |  |  | myosin complex; 4/20; 1.2748E-08 |  |
| **Feature 221** |  | protein-DNA complex assembly; 5/297; 1.3511E-04 spliceosomal complex; 6/297; 1.5850E-04 regulation of cellular localization; 3/297; 1.7221E-04 nuclear protein-containing complex; 16/297; 1.9269E-04 |  |  | myosin complex; 3/22; 3.5157E-06 |  |
| **Feature 170** |  | inward rectifier potassium channel activity; 3/157; 1.0381E-05 voltage-gated potassium channel activity; 3/157; 1.8355E-03 ligand-gated cation channel activity; 3/157; 2.1576E-03 voltage-gated cation channel activity; 3/157; 2.5126E-03 |  |  | proteasome-mediated ubiquitin-dependent protein catabolic process; 3/42; 1.0337E-04 proteasomal protein catabolic process; 3/42; 1.4609E-04 protein catabolic process; 3/42; 1.8012E-04 proteolysis; 4/42; 2.4377E-04 |  |
| **Feature 50** |  | inositol lipid-mediated signaling; 6/732; 4.7593E-07 phosphatidylinositol-mediated signaling; 6/732; 4.7593E-07 protein-containing complex; 77/732; 1.3957E-04 nuclear protein-containing complex; 28/732; 4.1144E-04 |  |  |  |  |
| **Feature 14** |  | nuclear protein-containing complex; 38/777; 7.1381E-08 nucleic acid metabolic process; 29/777; 2.2814E-05 protein-containing complex; 84/777; 2.3658E-05 nucleobase-containing compound metabolic process; 30/777; 1.4672E-04 |  |  |  |  |
| **Feature 204** |  | nuclear protein-containing complex; 23/487; 6.0052E-05 preribosome; 6/487; 1.0360E-04 protein modification by small protein conjugation; 8/487; 2.1038E-04 ribonucleoprotein complex; 15/487; 2.5186E-04 |  |  |  |  |
| **Feature 143** |  | RNA metabolic process; 12/301; 2.6978E-04 rRNA processing; 5/301; 1.2408E-03 nucleic acid metabolic process; 13/301; 1.2737E-03 brain development; 3/301; 1.3902E-03 |  |  | positive regulation of GTPase activity; 3/76; 3.5512E-04 activation of GTPase activity; 3/76; 3.5512E-04 regulation of GTPase activity; 3/76; 6.7767E-04 positive regulation of hydrolase activity; 3/76; 7.5871E-04 |  |
| **Feature 1** |  | regulation of nucleobase-containing compound metabolic process; 46/580; 6.4891E-04 regulation of metabolic process; 56/580; 7.1884E-04 regulation of macromolecule metabolic process; 54/580; 1.0759E-03 regulation of gene expression; 47/580; 1.8145E-03 |  |  | modification-dependent protein catabolic process; 3/57; 9.7427E-04 modification-dependent macromolecule catabolic process; 3/57; 9.7427E-04 ubiquitin-dependent protein catabolic process; 3/57; 9.7427E-04 proteolysis involved in cellular protein catabolic process; 3/57; 1.3870E-03 |  |
| **Feature 241** |  | intracellular ligand-gated ion channel activity; 3/384; 1.9128E-03 inorganic cation transmembrane transporter activity; 9/384; 2.9184E-03 microtubule cytoskeleton organization; 5/384; 3.5990E-03 protein-containing complex; 41/384; 3.8967E-03 |  |  | protein folding; 3/27; 5.4107E-06 proteasome-mediated ubiquitin-dependent protein catabolic process; 3/27; 2.6919E-05 proteasomal protein catabolic process; 3/27; 3.8160E-05 protein catabolic process; 3/27; 4.7141E-05 |  |
| **Feature 134** |  |  |  |  | chromatin binding; 3/27; 8.9220E-05 |  |
| **Feature 176** |  | inward rectifier potassium channel activity; 3/517; 3.6662E-04 intrinsic component of membrane; 10/517; 5.3011E-04 integral component of membrane; 10/517; 9.7505E-04 antigen receptor-mediated signaling pathway; 3/517; 1.7107E-03 |  |  | chromatin binding; 3/20; 3.5326E-05 |  |
| **Feature 127** |  | integrator complex; 5/436; 9.2070E-06 DNA recombination; 4/436; 1.6001E-03 integral component of membrane; 8/436; 1.8200E-03 cellular response to DNA damage stimulus; 6/436; 2.3060E-03 |  |  |  |  |
| **Feature 168** |  | phosphoric diester hydrolase activity; 3/213; 1.6367E-03 negative regulation of cell cycle process; 3/213; 2.0544E-03 negative regulation of cell cycle; 3/213; 3.6814E-03 ribonucleoprotein complex; 7/213; 8.5646E-03 |  |  | ubiquitin-protein transferase activity; 3/37; 4.7283E-04 ubiquitin-like protein transferase activity; 3/37; 6.3723E-04 |  |
| **Feature 203** |  | small-subunit processome; 3/265; 4.1471E-04 preribosome; 4/265; 8.6395E-04 tRNA modification; 3/265; 1.3494E-03 microtubule-based movement; 4/265; 1.5429E-03 |  |  |  |  |
| **Feature 199** |  | negative regulation of biological process; 10/161; 1.3144E-03 negative regulation of metabolic process; 6/161; 5.4691E-03 negative regulation of cellular process; 8/161; 8.7293E-03 |  |  |  |  |
| **Feature 251** |  | cell cycle process; 9/445; 8.3628E-04 cell-matrix adhesion; 3/445; 1.1060E-03 cell-substrate adhesion; 3/445; 1.1060E-03 endosome membrane; 4/445; 2.9475E-03 |  |  | cilium; 3/91; 5.2534E-03 negative regulation of cellular process; 6/91; 5.9762E-03 negative regulation of biological process; 6/91; 9.0951E-03 |  |
| **Feature 235** |  | brain development; 4/252; 2.7731E-05 nucleus; 29/252; 6.7184E-05 cellular\_component; 136/252; 8.2010E-04 membrane-bounded organelle; 42/252; 1.0019E-03 |  |  | negative regulation of cellular process; 3/20; 5.2513E-03 negative regulation of biological process; 3/20; 6.7227E-03 |  |
| **Feature 49** |  | protein polyubiquitination; 4/350; 1.2196E-03 microtubule-based process; 7/350; 1.7844E-03 biological\_process; 131/350; 1.9693E-03 system development; 4/350; 2.4137E-03 |  |  |  |  |
| **Feature 93** |  | organelle organization; 15/333; 2.9292E-04 regulation of intracellular signal transduction; 7/333; 1.4875E-03 regulation of mitotic cell cycle; 4/333; 2.9665E-03 maturation of SSU-rRNA; 3/333; 3.4969E-03 |  |  | peptide binding; 4/89; 4.5709E-06 amide binding; 4/89; 7.8745E-06 G protein-coupled receptor activity; 5/89; 2.3274E-04 transmembrane signaling receptor activity; 5/89; 1.5871E-03 |  |
| **Feature 184** |  | regulation of microtubule-based process; 5/751; 6.0166E-05 microtubule binding; 7/751; 7.0406E-04 microtubule organizing center; 12/751; 7.7372E-04 tubulin binding; 7/751; 1.1910E-03 |  |  | cytoplasm; 7/52; 1.0651E-03 protein metabolic process; 5/52; 8.4981E-03 |  |
| **Feature 231** |  | membrane-bounded organelle; 6/172; 1.9746E-03 cilium organization; 3/172; 6.9426E-03 regulation of catalytic activity; 5/172; 7.7467E-03 |  |  | myosin complex; 3/20; 2.6070E-06 |  |
| **Feature 68** |  | RNA metabolic process; 6/119; 3.0900E-03 microtubule organizing center; 4/119; 4.2981E-03 RNA splicing; 3/119; 4.9184E-03 centrosome; 3/119; 5.3535E-03 |  |  | protein localization to membrane; 3/59; 2.5221E-04 microtubule organizing center; 4/59; 3.1586E-04 localization within membrane; 3/59; 4.4697E-04 cellular protein localization; 3/59; 4.4789E-03 |  |
| **Feature 216** |  | centriolar satellite; 4/182; 2.8993E-05 microtubule organizing center; 5/182; 3.3718E-03 intracellular transport; 6/182; 6.5933E-03 membrane organization; 3/182; 7.2583E-03 |  |  | membrane-bounded organelle; 11/40; 1.5739E-03 organelle; 12/40; 1.7276E-03 intracellular membrane-bounded organelle; 9/40; 5.6461E-03 intracellular organelle; 10/40; 5.8756E-03 |  |
| **Feature 220** |  | ATP hydrolysis activity; 3/329; 7.8175E-04 microtubule-based process; 7/329; 1.2507E-03 microtubule cytoskeleton organization; 5/329; 1.8393E-03 centriole; 3/329; 2.5114E-03 |  |  |  |  |
| **Feature 64** |  | microtubule cytoskeleton organization; 3/97; 2.3073E-03 |  |  | cytoplasm; 9/67; 2.1438E-04 cellular\_component; 44/67; 2.9392E-04 protein-containing complex; 12/67; 1.7629E-03 biological\_process; 9/67; 2.8601E-03 |  |
| **Feature 236** |  | centriolar satellite; 3/150; 4.6029E-04 positive regulation of cellular protein metabolic process; 4/150; 1.2570E-03 positive regulation of protein metabolic process; 4/150; 1.2570E-03 positive regulation of catalytic activity; 4/150; 2.4514E-03 |  |  | inward rectifier potassium channel activity; 3/80; 1.3549E-06 voltage-gated potassium channel activity; 3/80; 2.5733E-04 ligand-gated cation channel activity; 3/80; 3.0402E-04 voltage-gated cation channel activity; 3/80; 3.5586E-04 |  |
| **Feature 45** |  | intracellular transport; 3/38; 5.0398E-03 establishment of localization in cell; 3/38; 7.3210E-03 RNA binding; 3/38; 8.0170E-03 binding; 10/38; 8.2708E-03 |  |  | protein kinase binding; 4/20; 1.8893E-07 kinase binding; 4/20; 2.1668E-07 enzyme binding; 4/20; 9.7986E-06 |  |
| **Feature 35** |  |  |  |  | protein kinase binding; 4/60; 1.7666E-05 kinase binding; 4/60; 2.0203E-05 nuclear speck; 3/60; 3.7883E-04 hexosyltransferase activity; 3/60; 4.6969E-04 |  |
| **Feature 5** |  | integrator complex; 5/234; 4.2710E-07 nucleocytoplasmic transport; 4/234; 1.8639E-03 nuclear transport; 4/234; 1.8639E-03 regulation of phosphorus metabolic process; 5/234; 3.2956E-03 |  |  | glycosyltransferase activity; 3/43; 1.0494E-03 membrane protein complex; 4/43; 2.3925E-03 |  |
| **Feature 150** |  |  |  |  |  |  |
| **Feature 254** |  | regulation of response to stimulus; 6/90; 1.9923E-03 negative regulation of signal transduction; 3/90; 3.1105E-03 negative regulation of cell communication; 3/90; 3.3579E-03 negative regulation of signaling; 3/90; 3.3579E-03 |  |  | protein kinase binding; 3/79; 1.1614E-03 kinase binding; 3/79; 1.2794E-03 RNA processing; 4/79; 5.7734E-03 negative regulation of macromolecule metabolic process; 4/79; 7.0402E-03 |  |
| **Feature 137** |  | keratin filament; 12/275; 3.6758E-09 intermediate filament; 13/275; 2.1682E-08 polymeric cytoskeletal fiber; 13/275; 2.7467E-07 supramolecular fiber; 13/275; 9.4125E-07 |  |  | keratin filament; 8/43; 1.7090E-11 intermediate filament; 8/43; 2.7423E-10 polymeric cytoskeletal fiber; 8/43; 1.4754E-09 supramolecular polymer; 8/43; 3.4127E-09 |  |
| **Feature 85** |  | glycosylation; 6/292; 1.4781E-05 fucosylation; 3/292; 6.6772E-05 cation transmembrane transport; 7/292; 4.4455E-04 metal ion transport; 4/292; 7.9302E-04 |  |  | organic substance catabolic process; 3/38; 2.6689E-03 cellular catabolic process; 3/38; 2.8558E-03 catabolic process; 3/38; 4.1468E-03 |  |
| **Feature 46** |  | regulation of developmental process; 3/129; 8.4809E-03 |  |  |  |  |
| **Feature 99** |  | keratin filament; 20/426; 1.3592E-15 intermediate filament; 22/426; 1.6302E-14 polymeric cytoskeletal fiber; 22/426; 1.7368E-12 supramolecular polymer; 22/426; 1.6346E-11 |  |  | polymeric cytoskeletal fiber; 24/31; 4.9585E-46 intermediate filament; 24/31; 1.4697E-48 keratin filament; 23/31; 7.4466E-50 supramolecular fiber; 24/31; 8.6324E-45 |  |
| **Feature 120** |  | calcium channel activity; 3/443; 5.8242E-03 adherens junction; 3/443; 5.8242E-03 modification-dependent protein binding; 3/443; 7.7778E-03 |  |  | oxidoreductase activity; 4/46; 4.8038E-05 extracellular space; 5/46; 2.3814E-04 extracellular region; 3/46; 1.8303E-03 |  |
| **Feature 102** |  | anatomical structure morphogenesis; 3/78; 2.5924E-03 hydrolase activity, acting on ester bonds; 4/78; 3.6920E-03 cell projection organization; 3/78; 4.6244E-03 cellular developmental process; 4/78; 5.5191E-03 |  |  | catalytic activity; 8/37; 5.6115E-04 |  |
| **Feature 117** |  | animal organ development; 3/120; 2.5319E-03 |  |  | intermediate filament; 6/42; 2.7735E-07 polymeric cytoskeletal fiber; 6/42; 9.5260E-07 supramolecular polymer; 6/42; 1.7623E-06 supramolecular fiber; 6/42; 1.7623E-06 |  |
| **Feature 132** |  | transcription regulator activity; 13/153; 7.2323E-04 DNA-binding transcription factor activity, RNA polymerase II-specific; 11/153; 1.5041E-03 DNA-binding transcription factor activity; 11/153; 2.3259E-03 regulation of transcription by RNA polymerase II; 13/153; 4.2848E-03 |  |  |  |  |
| **Feature 83** |  | mRNA export from nucleus; 3/224; 1.4655E-04 mRNA transport; 3/224; 1.4655E-04 nuclear export; 4/224; 2.2577E-04 establishment of RNA localization; 3/224; 1.1234E-03 |  |  | nucleosome organization; 8/28; 1.4636E-16 protein-DNA complex subunit organization; 8/28; 2.2407E-16 nucleosome assembly; 7/28; 1.2449E-14 protein-DNA complex assembly; 7/28; 1.9121E-14 |  |
| **Feature 228** |  | endocytosis; 3/137; 3.5275E-04 plasma membrane protein complex; 5/137; 1.0404E-03 membrane protein complex; 6/137; 9.4540E-03 |  |  |  |  |
| **Feature 48** |  | regulation of RNA splicing; 3/196; 7.6246E-04 phosphoprotein phosphatase activity; 4/196; 1.1217E-03 protein tyrosine phosphatase activity; 3/196; 2.4276E-03 microtubule-based movement; 3/196; 6.1856E-03 |  |  | regulation of cellular macromolecule biosynthetic process; 4/17; 7.1921E-03 regulation of cellular biosynthetic process; 4/17; 7.2394E-03 regulation of macromolecule biosynthetic process; 4/17; 7.2869E-03 regulation of biosynthetic process; 4/17; 7.3826E-03 |  |
| **Feature 118** |  | plasma membrane protein complex; 3/75; 8.7267E-03 |  |  |  |  |
| **Feature 121** |  | centriole; 4/369; 2.0000E-04 system development; 5/369; 2.8616E-04 central nervous system development; 3/369; 1.0922E-03 cilium movement; 3/369; 2.4965E-03 |  |  |  |  |
| **Feature 76** |  | nucleoplasm; 11/457; 1.1835E-04 biological\_process; 171/457; 3.7215E-04 cellular process; 101/457; 1.6105E-03 carbohydrate derivative biosynthetic process; 6/457; 1.8381E-03 |  |  |  |  |
| **Feature 96** |  | microtubule organizing center; 6/264; 3.4044E-03 chromatin remodeling; 3/264; 4.6422E-03 metalloendopeptidase activity; 3/264; 6.7031E-03 voltage-gated potassium channel activity; 3/264; 7.9067E-03 |  |  |  |  |
| **Feature 36** |  | microtubule binding; 4/176; 4.7143E-04 tubulin binding; 4/176; 6.4737E-04 cytoskeletal protein binding; 5/176; 1.4695E-03 catalytic activity, acting on DNA; 3/176; 8.2269E-03 |  |  |  |  |
| **Feature 212** |  |  |  |  | negative regulation of cellular process; 3/20; 5.2513E-03 negative regulation of biological process; 3/20; 6.7227E-03 |  |
| **Feature 87** |  |  |  |  |  |  |
| **Feature 195** |  | PcG protein complex; 4/470; 3.1361E-04 DNA helicase activity; 3/470; 1.2969E-03 cell projection; 12/470; 3.8676E-03 microtubule organizing center; 8/470; 4.3464E-03 |  |  | keratin filament; 5/58; 7.0613E-06 intermediate filament; 5/58; 3.6518E-05 monocarboxylic acid metabolic process; 3/58; 5.5686E-05 polymeric cytoskeletal fiber; 5/58; 9.8660E-05 |  |
| **Feature 194** |  | protein kinase binding; 6/199; 1.1466E-05 kinase binding; 6/199; 1.4010E-05 enzyme binding; 8/199; 7.1290E-05 response to abiotic stimulus; 3/199; 3.5993E-03 |  |  | chemokine receptor binding; 3/90; 1.6650E-05 cytokine receptor binding; 3/90; 7.6699E-05 G protein-coupled receptor binding; 3/90; 5.8362E-04 small molecule metabolic process; 4/90; 2.0685E-03 |  |
| **Feature 79** |  | glycoprotein complex; 3/621; 6.3144E-04 dystrophin-associated glycoprotein complex; 3/621; 6.3144E-04 nuclear transport; 6/621; 2.3903E-03 nucleocytoplasmic transport; 6/621; 2.3903E-03 |  |  | negative regulation of nucleobase-containing compound metabolic process; 3/18; 1.5199E-04 negative regulation of cellular process; 4/18; 2.5753E-04 negative regulation of biological process; 4/18; 3.6175E-04 negative regulation of nitrogen compound metabolic process; 3/18; 4.4043E-04 |  |
| **Feature 115** |  | intrinsic component of synaptic membrane; 4/580; 1.1202E-03 integral component of synaptic membrane; 4/580; 1.1202E-03 positive regulation of canonical Wnt signaling pathway; 3/580; 1.2401E-03 regulation of cytoskeleton organization; 5/580; 1.7070E-03 |  |  |  |  |
| **Feature 129** |  | extracellular exosome; 18/683; 4.9291E-04 transcription coregulator activity; 9/683; 7.4403E-04 vesicle cargo loading; 3/683; 8.3684E-04 extracellular membrane-bounded organelle; 18/683; 9.8813E-04 |  |  | extracellular membrane-bounded organelle; 3/32; 5.5213E-03 extracellular organelle; 3/32; 5.5213E-03 extracellular vesicle; 3/32; 5.5213E-03 |  |
| **Feature 16** |  | nuclear speck; 6/257; 2.5684E-05 nuclear body; 6/257; 6.3712E-04 anatomical structure morphogenesis; 5/257; 1.9984E-03 nucleoplasm; 6/257; 5.4081E-03 |  |  | extracellular exosome; 6/20; 5.5052E-08 extracellular organelle; 6/20; 6.3451E-08 extracellular vesicle; 6/20; 6.3451E-08 extracellular membrane-bounded organelle; 6/20; 6.3451E-08 |  |
| **Feature 208** |  | intermediate filament; 15/300; 7.0833E-10 polymeric cytoskeletal fiber; 15/300; 1.4185E-08 supramolecular fiber; 15/300; 6.0615E-08 supramolecular polymer; 15/300; 6.0615E-08 |  |  | extracellular exosome; 5/34; 3.2701E-05 extracellular vesicle; 5/34; 3.6652E-05 extracellular organelle; 5/34; 3.6652E-05 extracellular membrane-bounded organelle; 5/34; 3.6652E-05 |  |
| **Feature 190** |  | regulation of DNA recombination; 3/321; 7.2751E-04 protein localization to nucleus; 3/321; 2.3409E-03 positive regulation of cellular component biogenesis; 3/321; 6.5271E-03 transcription corepressor activity; 3/321; 7.9895E-03 |  |  |  |  |
| **Feature 53** |  | histone binding; 9/616; 1.6279E-06 ribonucleoprotein complex; 21/616; 2.6683E-06 nucleoplasm; 14/616; 2.3328E-05 spliceosomal complex; 8/616; 2.4206E-04 |  |  | RNA binding; 3/20; 1.2487E-03 |  |
| **Feature 82** |  | metal ion transport; 5/350; 1.2533E-04 zinc ion transport; 3/350; 2.8024E-04 zinc ion transmembrane transport; 3/350; 2.8024E-04 RNA binding; 12/350; 4.1889E-04 |  |  | cell adhesion; 6/55; 2.6861E-07 biological adhesion; 6/55; 2.6861E-07 integral component of plasma membrane; 8/55; 3.1740E-07 intrinsic component of plasma membrane; 8/55; 3.7399E-07 |  |
| **Feature 112** |  | histone acetylation; 4/570; 6.5559E-04 internal protein amino acid acetylation; 4/570; 6.5559E-04 histone deacetylase complex; 4/570; 6.5559E-04 peptidyl-lysine acetylation; 4/570; 6.5559E-04 |  |  | negative regulation of cellular process; 4/19; 3.2163E-04 negative regulation of biological process; 4/19; 4.5117E-04 positive regulation of cellular process; 3/19; 6.5471E-03 positive regulation of biological process; 3/19; 9.6162E-03 |  |
| **Feature 91** |  | mitotic spindle; 3/483; 1.4041E-03 organonitrogen compound metabolic process; 5/483; 6.1482E-03 extracellular matrix; 9/483; 8.7095E-03 external encapsulating structure; 9/483; 8.7095E-03 |  |  |  |  |
| **Feature 152** |  | preribosome; 6/468; 8.3073E-05 extracellular matrix organization; 4/468; 5.5699E-03 extracellular structure organization; 4/468; 5.5699E-03 external encapsulating structure organization; 4/468; 5.5699E-03 |  |  | collagen trimer; 7/135; 1.7504E-10 extracellular matrix structural constituent; 3/135; 3.2370E-05 extracellular matrix; 7/135; 5.7536E-05 external encapsulating structure; 7/135; 5.7536E-05 |  |
| **Feature 21** |  | adherens junction; 3/134; 1.8344E-04 cell-cell junction; 4/134; 5.2305E-04 cell junction; 6/134; 1.1231E-03 anchoring junction; 4/134; 1.7695E-03 |  |  | chromatin binding; 3/49; 5.3483E-04 intracellular protein-containing complex; 4/49; 1.3236E-03 ribonucleoprotein complex; 4/49; 1.9317E-03 nuclear protein-containing complex; 5/49; 2.3797E-03 |  |
| **Feature 146** |  | intracellular signal transduction; 7/348; 6.5058E-03 |  |  |  |  |
| **Feature 54** |  | protein serine/threonine kinase activity; 10/643; 5.0255E-04 synaptic vesicle membrane; 4/643; 2.4734E-03 exocytic vesicle membrane; 4/643; 2.4734E-03 transport vesicle membrane; 4/643; 4.8923E-03 |  |  | G protein-coupled receptor activity; 5/72; 8.5196E-05 signaling receptor activity; 6/72; 2.7820E-04 molecular transducer activity; 6/72; 2.7820E-04 transmembrane signaling receptor activity; 5/72; 6.0804E-04 |  |
| **Feature 106** |  | collagen trimer; 4/214; 1.4422E-04 protein phosphatase regulator activity; 3/214; 1.2940E-03 phosphatase regulator activity; 3/214; 2.0820E-03 extracellular matrix; 6/214; 4.8073E-03 |  |  | polymeric cytoskeletal fiber; 11/64; 2.5702E-12 supramolecular fiber; 11/64; 8.2663E-12 supramolecular polymer; 11/64; 8.2663E-12 supramolecular complex; 11/64; 9.4824E-11 |  |
| **Feature 248** |  |  |  |  | cellular developmental process; 4/68; 3.3765E-03 cell differentiation; 3/68; 7.5610E-03 protein binding; 9/68; 8.5372E-03 |  |
| **Feature 148** |  | supramolecular polymer; 11/254; 1.5701E-05 supramolecular fiber; 11/254; 1.5701E-05 supramolecular complex; 11/254; 1.2254E-04 polymeric cytoskeletal fiber; 9/254; 1.9807E-04 |  |  | extracellular vesicle; 5/25; 7.5931E-06 extracellular organelle; 5/25; 7.5931E-06 extracellular membrane-bounded organelle; 5/25; 7.5931E-06 vesicle; 5/25; 1.1907E-04 |  |
| **Feature 207** |  | synaptic signaling; 4/381; 9.6881E-04 cell-cell signaling; 4/381; 2.6618E-03 signaling; 4/381; 3.2831E-03 cell communication; 4/381; 4.8073E-03 |  |  | polymeric cytoskeletal fiber; 13/47; 2.7300E-17 intermediate filament; 12/47; 1.0511E-16 supramolecular polymer; 13/47; 1.1298E-16 supramolecular fiber; 13/47; 1.1298E-16 |  |
| **Feature 247** |  | collagen trimer; 7/410; 3.9017E-07 catalytic activity; 5/410; 2.2916E-05 keratinization; 4/410; 4.5790E-04 dendritic spine; 3/410; 2.3090E-03 |  |  |  |  |
| **Feature 141** |  | immune response-regulating signaling pathway; 3/318; 4.0070E-03 nuclear transport; 4/318; 5.6151E-03 nucleocytoplasmic transport; 4/318; 5.6151E-03 |  |  |  |  |
| **Feature 61** |  | maturation of SSU-rRNA; 3/315; 2.9871E-03 |  |  | respiratory chain complex; 3/244; 3.0243E-03 ribonucleoprotein complex assembly; 3/244; 3.0243E-03 ribonucleoprotein complex subunit organization; 3/244; 3.7206E-03 protein-containing complex subunit organization; 6/244; 3.9209E-03 |  |
| **Feature 80** |  | enzyme inhibitor activity; 6/489; 1.0598E-04 phosphatase inhibitor activity; 4/489; 1.0872E-04 phosphatase regulator activity; 5/489; 2.1381E-04 postsynapse; 4/489; 1.2930E-03 |  |  | G protein-coupled receptor activity; 8/238; 1.1571E-04 molecular transducer activity; 10/238; 8.2473E-04 signaling receptor activity; 10/238; 8.2473E-04 peptide receptor activity; 4/238; 8.6208E-04 |  |
| **Feature 188** |  | regulation of cell adhesion; 3/310; 2.8543E-03 regulation of lymphocyte activation; 3/310; 2.8543E-03 cellular process; 71/310; 3.1794E-03 cellular\_component; 110/310; 3.6478E-03 |  |  |  |  |
| **Feature 185** |  | extracellular matrix; 14/402; 1.1524E-06 external encapsulating structure; 14/402; 1.1524E-06 collagen trimer; 4/402; 1.5692E-03 collagen-containing extracellular matrix; 7/402; 1.8103E-03 |  |  | extracellular space; 3/20; 1.7750E-03 |  |
| **Feature 196** |  | regulation of gene expression, epigenetic; 3/446; 2.3630E-04 catalytic activity, acting on DNA; 6/446; 5.3001E-04 negative regulation of nucleobase-containing compound metabolic process; 8/446; 4.6462E-03 cell projection; 11/446; 7.2520E-03 |  |  | regulation of nucleobase-containing compound metabolic process; 6/33; 4.3095E-03 regulation of nitrogen compound metabolic process; 6/33; 8.4925E-03 regulation of primary metabolic process; 6/33; 8.8848E-03 regulation of cellular metabolic process; 6/33; 9.9229E-03 |  |
| **Feature 133** |  |  |  |  | cytoplasm; 15/205; 1.8688E-03 histone acetyltransferase complex; 3/205; 3.9131E-03 protein acetyltransferase complex; 3/205; 4.5853E-03 acetyltransferase complex; 3/205; 4.5853E-03 |  |
| **Feature 24** |  | negative regulation of cellular component organization; 5/185; 5.8422E-06 negative regulation of organelle organization; 4/185; 2.0877E-05 negative regulation of supramolecular fiber organization; 3/185; 1.4345E-04 negative regulation of cytoskeleton organization; 3/185; 1.4345E-04 |  |  | protein transport; 3/52; 3.4172E-03 establishment of protein localization; 3/52; 4.0355E-03 nitrogen compound transport; 3/52; 9.9660E-03 |  |
| **Feature 159** |  | inward rectifier potassium channel activity; 3/165; 1.2055E-05 ligand-gated cation channel activity; 4/165; 1.4249E-04 presynapse; 3/165; 1.6164E-04 ligand-gated ion channel activity; 4/165; 3.6904E-04 |  |  | myosin complex; 3/74; 1.4149E-04 catalytic complex; 5/74; 8.1850E-03 |  |
| **Feature 3** |  | actin filament organization; 3/190; 2.2219E-03 |  |  | cellular component assembly; 3/37; 7.2770E-03 |  |
| **Feature 180** |  | histone demethylase activity; 3/209; 1.1928E-04 demethylase activity; 3/209; 1.1928E-04 protein demethylase activity; 3/209; 1.1928E-04 ciliary transition zone; 3/209; 3.2495E-04 |  |  |  |  |
| **Feature 239** |  | cell cycle process; 6/195; 7.9313E-04 secretory vesicle; 4/195; 1.4500E-03 mitotic cell cycle process; 4/195; 1.4500E-03 endosomal transport; 3/195; 1.9688E-03 |  |  |  |  |
| **Feature 92** |  |  |  |  |  |  |
| **Feature 232** |  | Golgi cisterna; 3/539; 4.1491E-04 Golgi apparatus subcompartment; 5/539; 3.2290E-03 histone binding; 5/539; 3.9669E-03 organelle subcompartment; 5/539; 4.8178E-03 |  |  |  |  |
| **Feature 249** |  | microtubule-based process; 6/202; 5.1144E-04 establishment of organelle localization; 3/202; 1.0953E-03 centriolar satellite; 3/202; 1.0953E-03 microtubule cytoskeleton organization; 4/202; 2.1271E-03 |  |  | microtubule organizing center; 4/39; 6.2039E-05 |  |
| **Feature 28** |  |  |  |  |  |  |
| **Feature 98** |  | histone deacetylase complex; 3/293; 1.2860E-03 transcription coregulator activity; 5/293; 4.3697E-03 DNA repair; 4/293; 4.8325E-03 cellular localization; 10/293; 7.0453E-03 |  |  | myosin complex; 3/31; 1.0182E-05 cellular\_component; 23/31; 7.9537E-04 protein binding; 6/31; 4.7614E-03 |  |
| **Feature 58** |  |  |  |  | cell adhesion; 3/21; 1.4341E-04 biological adhesion; 3/21; 1.4341E-04 negative regulation of cellular process; 4/21; 4.8280E-04 negative regulation of biological process; 4/21; 6.7545E-04 |  |
| **Feature 175** |  | nucleoplasm; 11/486; 2.0308E-04 transport vesicle; 5/486; 3.0065E-04 synaptic vesicle; 4/486; 5.7424E-04 elongin complex; 3/486; 7.3833E-04 |  |  | nucleosome organization; 3/27; 1.8110E-05 protein-DNA complex subunit organization; 3/27; 2.0794E-05 chromatin organization; 3/27; 4.1735E-04 protein-containing complex subunit organization; 3/27; 5.9643E-04 |  |
| **Feature 172** |  | microtubule-based process; 7/291; 6.0880E-04 microtubule cytoskeleton organization; 5/291; 1.0671E-03 centriole; 3/291; 1.7670E-03 cell cycle process; 6/291; 5.9049E-03 |  |  | intermediate filament; 26/86; 6.2437E-38 polymeric cytoskeletal fiber; 27/86; 4.5488E-37 keratin filament; 23/86; 3.8766E-36 supramolecular polymer; 27/86; 1.1562E-35 |  |
| **Feature 197** |  | centrosome; 4/96; 1.8572E-04 microtubule organizing center; 4/96; 1.9774E-03 chromosome organization; 3/96; 2.4567E-03 regulation of biological quality; 5/96; 3.1401E-03 |  |  | organelle organization; 5/69; 4.8765E-03 cellular component organization; 7/69; 8.5704E-03 cellular component organization or biogenesis; 7/69; 9.0476E-03 |  |
| **Feature 230** |  |  |  |  |  |  |
| **Feature 107** |  |  |  |  |  |  |
| **Feature 10** |  | potassium ion transport; 3/295; 9.0976E-03 potassium ion transmembrane transport; 3/295; 9.0976E-03 |  |  |  |  |
| **Feature 151** |  | integrator complex; 5/412; 6.9783E-06 centriole; 4/412; 3.0560E-04 |  |  | ciliary basal body; 3/81; 3.1538E-04 |  |
| **Feature 15** |  | negative regulation of mitotic cell cycle phase transition; 4/226; 5.1769E-06 negative regulation of cell cycle process; 5/226; 5.2589E-06 negative regulation of cell cycle; 5/226; 1.5477E-05 negative regulation of mitotic cell cycle; 4/226; 4.5824E-05 |  |  |  |  |
| **Feature 243** |  | cell adhesion molecule binding; 3/261; 5.4400E-03 dendrite; 3/261; 6.4944E-03 |  |  | catalytic activity; 5/20; 3.1935E-03 |  |
| **Feature 179** |  | cellular metal ion homeostasis; 3/83; 1.7722E-03 cellular cation homeostasis; 3/83; 2.6689E-03 cellular chemical homeostasis; 3/83; 2.6689E-03 metal ion homeostasis; 3/83; 2.6689E-03 |  |  |  |  |
| **Feature 62** |  | nucleus; 9/67; 7.5915E-03 |  |  | Golgi apparatus; 3/48; 2.0699E-03 |  |
| **Feature 154** |  | cytoskeletal protein binding; 6/199; 3.5125E-04 microtubule binding; 4/199; 7.4875E-04 tubulin binding; 4/199; 1.0249E-03 ciliary basal body; 3/199; 4.2193E-03 |  |  | cilium; 3/105; 7.8066E-03 |  |
| **Feature 73** |  | nuclear membrane protein complex; 3/367; 3.3862E-05 nuclear membrane microtubule tethering complex; 3/367; 3.3862E-05 meiotic nuclear membrane microtubule tethering complex; 3/367; 3.3862E-05 protein kinase binding; 7/367; 3.8098E-05 |  |  |  |  |
| **Feature 219** |  | double-stranded DNA binding; 12/154; 6.3803E-04 RNA polymerase II transcription regulatory region sequence-specific DNA binding; 11/154; 1.2581E-03 transcription cis-regulatory region binding; 11/154; 1.4550E-03 transcription regulatory region nucleic acid binding; 11/154; 1.4550E-03 |  |  | mitochondrion; 4/84; 8.1765E-03 |  |
| **Feature 111** |  | integrator complex; 5/251; 6.0517E-07 single-stranded RNA binding; 3/251; 5.5634E-04 detection of stimulus; 3/251; 1.1535E-03 macromolecule metabolic process; 21/251; 3.8368E-03 |  |  | vesicle; 4/20; 5.9381E-04 extracellular vesicle; 3/20; 1.4007E-03 extracellular membrane-bounded organelle; 3/20; 1.4007E-03 extracellular organelle; 3/20; 1.4007E-03 |  |
| **Feature 7** |  | microtubule organizing center; 4/115; 3.8043E-03 intracellular transport; 5/115; 4.1134E-03 centrosome; 3/115; 4.8668E-03 establishment of localization in cell; 5/115; 7.2476E-03 |  |  | elongin complex; 3/90; 4.8124E-06 peptide binding; 3/90; 2.0668E-04 G protein-coupled receptor activity; 5/90; 2.4524E-04 transcription elongation factor complex; 3/90; 2.5291E-04 |  |
| **Feature 42** |  | sodium channel complex; 3/271; 6.9614E-04 nucleoside-triphosphatase regulator activity; 5/271; 1.9970E-03 GTPase regulator activity; 5/271; 1.9970E-03 GTPase activator activity; 5/271; 1.9970E-03 |  |  |  |  |
| **Feature 97** |  | microtubule organizing center; 6/167; 3.2032E-04 microtubule-based movement; 3/167; 3.9575E-03 microtubule-based process; 4/167; 9.8548E-03 |  |  | chromatin binding; 3/92; 3.3101E-03 active transmembrane transporter activity; 3/92; 7.3177E-03 |  |
| **Feature 108** |  |  |  |  | microtubule organizing center; 3/20; 1.7533E-04 organelle organization; 3/20; 3.7570E-03 |  |
| **Feature 167** |  | cytoskeletal protein binding; 12/605; 2.1235E-05 microtubule binding; 7/605; 1.8798E-04 anatomical structure morphogenesis; 9/605; 2.0689E-04 regulation of cysteine-type endopeptidase activity; 4/605; 2.4891E-04 |  |  |  |  |
| **Feature 233** |  | import into nucleus; 3/263; 9.4028E-04 protein import into nucleus; 3/263; 9.4028E-04 protein localization to nucleus; 3/263; 1.3202E-03 protein import; 3/263; 3.7381E-03 |  |  |  |  |
| **Feature 128** |  |  |  |  | hydrolase activity; 3/20; 5.3958E-03 |  |
| **Feature 211** |  | Notch signaling pathway; 3/277; 7.4209E-04 histone deacetylase complex; 3/277; 1.0930E-03 protein-containing complex localization; 3/277; 2.0703E-03 phosphatidylinositol phosphate binding; 3/277; 2.7107E-03 |  |  | small molecule metabolic process; 6/61; 1.6124E-06 cellular metabolic process; 13/61; 1.8350E-05 mitochondrion; 6/61; 2.2625E-05 phosphate-containing compound metabolic process; 6/61; 3.9402E-05 |  |
| **Feature 198** |  | cell junction; 3/38; 4.3911E-03 |  |  | ribonucleoprotein complex; 6/18; 2.4368E-08 extracellular exosome; 5/18; 1.1600E-06 extracellular membrane-bounded organelle; 5/18; 1.3048E-06 extracellular vesicle; 5/18; 1.3048E-06 |  |
| **Feature 95** |  | protein kinase binding; 7/166; 1.9761E-07 kinase binding; 7/166; 2.5212E-07 3',5'-cyclic-nucleotide phosphodiesterase activity; 3/166; 6.0060E-05 cyclic-nucleotide phosphodiesterase activity; 3/166; 6.0060E-05 |  |  | intermediate filament; 7/20; 2.7743E-11 polymeric cytoskeletal fiber; 7/20; 1.2266E-10 supramolecular polymer; 7/20; 2.5802E-10 supramolecular fiber; 7/20; 2.5802E-10 |  |
| **Feature 218** |  | integrator complex; 5/308; 1.6675E-06 translation regulator activity; 4/308; 7.5649E-04 RNA helicase activity; 3/308; 1.0107E-03 ATPase, acting on RNA; 3/308; 1.0107E-03 |  |  |  |  |
| **Feature 202** |  | cellular protein localization; 4/125; 5.4127E-03 cellular macromolecule localization; 4/125; 5.7192E-03 |  |  |  |  |
| **Feature 110** |  | nuclear body; 5/171; 6.8285E-04 nucleolus; 5/171; 1.7850E-03 |  |  |  |  |
| **Feature 160** |  | voltage-gated cation channel activity; 3/132; 1.5304E-03 voltage-gated channel activity; 3/132; 1.7703E-03 voltage-gated ion channel activity; 3/132; 1.7703E-03 gated channel activity; 4/132; 2.1417E-03 |  |  | extracellular space; 5/28; 2.0388E-05 |  |
| **Feature 165** |  | neuromuscular synaptic transmission; 3/335; 1.0067E-04 biological\_process; 129/335; 5.2858E-04 cellular process; 76/335; 3.4906E-03 cell differentiation; 7/335; 3.5509E-03 |  |  |  |  |
| **Feature 224** |  | helicase activity; 3/302; 5.5084E-03 external encapsulating structure; 7/302; 6.6200E-03 extracellular matrix; 7/302; 6.6200E-03 cell junction organization; 3/302; 8.1467E-03 |  |  | extracellular space; 3/20; 1.7750E-03 developmental process; 3/20; 8.6107E-03 |  |
| **Feature 201** |  |  |  |  |  |  |
| **Feature 55** |  | plasma membrane protein complex; 4/117; 4.2819E-03 cell-cell junction; 3/117; 4.2966E-03 protein-containing complex binding; 3/117; 6.0027E-03 |  |  | macromolecule metabolic process; 4/20; 9.4159E-03 |  |
| **Feature 215** |  | protein-containing complex localization; 4/464; 7.3210E-04 heart development; 3/464; 1.2493E-03 single-stranded DNA binding; 3/464; 1.2493E-03 microtubule cytoskeleton organization; 6/464; 1.4147E-03 |  |  | protein kinase binding; 3/50; 3.0316E-04 kinase binding; 3/50; 3.3460E-04 enzyme binding; 3/50; 5.0730E-03 |  |
| **Feature 130** |  | RNA binding; 8/161; 3.6425E-04 proton transmembrane transporter activity; 3/161; 5.6620E-04 |  |  | nuclear protein-containing complex; 3/22; 8.1881E-03 |  |
| **Feature 191** |  | synaptic signaling; 3/146; 6.9035E-04 cell-cell signaling; 3/146; 1.4901E-03 signaling; 3/146; 1.7529E-03 kinase regulator activity; 3/146; 2.0429E-03 |  |  | extracellular exosome; 10/41; 1.5803E-11 extracellular membrane-bounded organelle; 10/41; 2.0060E-11 extracellular vesicle; 10/41; 2.0060E-11 extracellular organelle; 10/41; 2.0060E-11 |  |
| **Feature 209** |  |  |  |  |  |  |
| **Feature 192** |  | nuclear transport; 4/88; 4.4822E-05 nucleocytoplasmic transport; 4/88; 4.4822E-05 nitrogen compound transport; 5/88; 9.3281E-04 intracellular transport; 5/88; 1.2757E-03 |  |  | response to external stimulus; 3/41; 5.6394E-04 extracellular region; 3/41; 1.3097E-03 hydrolase activity, acting on ester bonds; 3/41; 4.4363E-03 |  |
| **Feature 166** |  | microtubule binding; 4/205; 8.3682E-04 tubulin binding; 4/205; 1.1445E-03 cellular\_component; 68/205; 2.7476E-03 cytokine-mediated signaling pathway; 3/205; 2.7580E-03 |  |  |  |  |
| **Feature 38** |  | keratinization; 3/183; 6.2423E-04 intermediate filament; 6/183; 1.2289E-03 polymeric cytoskeletal fiber; 6/183; 3.5293E-03 supramolecular fiber; 6/183; 5.8800E-03 |  |  | polymeric cytoskeletal fiber; 19/20; 4.2681E-40 supramolecular polymer; 19/20; 3.8299E-39 supramolecular fiber; 19/20; 3.8299E-39 intermediate filament; 18/20; 9.6190E-39 |  |
| **Feature 56** |  | chromosome organization involved in meiotic cell cycle; 3/298; 5.8526E-04 chromosome organization; 5/298; 1.3764E-03 regulation of mitotic cell cycle phase transition; 3/298; 3.3353E-03 reproductive process; 6/298; 4.8184E-03 |  |  | supramolecular fiber; 25/32; 7.5399E-47 supramolecular polymer; 25/32; 7.5399E-47 intermediate filament; 24/32; 5.8537E-48 polymeric cytoskeletal fiber; 25/32; 3.7686E-48 |  |
| **Feature 173** |  | keratinization; 3/80; 5.3926E-05 |  |  | polymeric cytoskeletal fiber; 14/19; 1.8775E-26 supramolecular polymer; 14/19; 8.9787E-26 supramolecular fiber; 14/19; 8.9787E-26 intermediate filament; 13/19; 3.4707E-25 |  |
| **Feature 214** |  | intermediate filament; 14/145; 4.0346E-13 keratin filament; 12/145; 2.1089E-12 polymeric cytoskeletal fiber; 14/145; 7.8522E-12 supramolecular polymer; 14/145; 3.3812E-11 |  |  | polymeric cytoskeletal fiber; 16/26; 3.8118E-28 supramolecular polymer; 16/26; 2.3208E-27 supramolecular fiber; 16/26; 2.3208E-27 intermediate filament; 15/26; 2.7659E-27 |  |
| **Feature 178** |  | microtubule-based process; 4/151; 6.9617E-03 microtubule cytoskeleton organization; 3/151; 7.9673E-03 cytoskeleton organization; 4/151; 9.3416E-03 |  |  | keratin filament; 31/118; 1.9772E-49 polymeric cytoskeletal fiber; 35/118; 2.0451E-47 intermediate filament; 34/118; 2.2925E-49 supramolecular polymer; 35/118; 1.7327E-45 |  |
| **Feature 126** |  | inward rectifier potassium channel activity; 3/397; 1.6708E-04 axon; 4/397; 8.2978E-04 centriole; 3/397; 4.2803E-03 regulation of cellular macromolecule biosynthetic process; 31/397; 5.4272E-03 |  |  | keratin filament; 11/34; 2.0797E-18 polymeric cytoskeletal fiber; 12/34; 1.6431E-17 supramolecular fiber; 12/34; 6.0968E-17 supramolecular polymer; 12/34; 6.0968E-17 |  |
| **Feature 119** |  | phosphatase activity; 3/77; 3.5025E-03 nuclear protein-containing complex; 6/77; 3.5498E-03 phosphoric ester hydrolase activity; 3/77; 7.1504E-03 cytoskeleton organization; 3/77; 8.5960E-03 |  |  | keratin filament; 4/19; 1.5789E-06 intermediate filament; 4/19; 6.0672E-06 polymeric cytoskeletal fiber; 4/19; 1.3840E-05 supramolecular polymer; 4/19; 2.0919E-05 |  |
| **Feature 147** |  | ribonucleoprotein complex; 7/148; 1.1405E-03 ion channel activity; 5/148; 1.8019E-03 channel activity; 5/148; 2.7942E-03 passive transmembrane transporter activity; 5/148; 2.7942E-03 |  |  | catalytic activity; 6/29; 3.4998E-03 supramolecular complex; 3/29; 3.9887E-03 |  |
| **Feature 210** |  | kinase activity; 7/158; 6.2029E-04 protein serine/threonine kinase activity; 5/158; 6.9754E-04 transferase activity, transferring phosphorus-containing groups; 7/158; 1.1847E-03 protein kinase activity; 6/158; 1.1890E-03 |  |  |  |  |
| **Feature 177** |  | phosphoprotein phosphatase activity; 3/63; 4.3752E-04 protein localization to organelle; 3/63; 1.6181E-03 phosphatase activity; 3/63; 1.9765E-03 catalytic activity, acting on a protein; 6/63; 3.4683E-03 |  |  | extracellular exosome; 16/106; 2.9607E-14 extracellular vesicle; 16/106; 4.3349E-14 extracellular organelle; 16/106; 4.3349E-14 extracellular membrane-bounded organelle; 16/106; 4.3349E-14 |  |
| **Feature 238** |  | cellular anatomical entity; 75/160; 6.3027E-03 microtubule cytoskeleton organization; 3/160; 9.3333E-03 |  |  |  |  |
| **Feature 104** |  | keratinization; 5/131; 8.4230E-08 multicellular organismal process; 6/131; 1.4171E-04 integral component of synaptic membrane; 3/131; 1.7155E-04 intrinsic component of synaptic membrane; 3/131; 1.7155E-04 |  |  | keratinization; 5/20; 4.6099E-12 multicellular organismal process; 5/20; 1.0516E-07 |  |
| **Feature 227** |  | microtubule binding; 6/196; 2.5985E-06 tubulin binding; 6/196; 4.3182E-06 cytoskeletal protein binding; 7/196; 3.7400E-05 intracellular protein-containing complex; 8/196; 6.7691E-04 |  |  | mitochondrion; 3/40; 6.3026E-03 |  |
| **Feature 229** |  | Golgi organization; 5/385; 1.7340E-05 keratinization; 4/385; 3.6021E-04 mRNA export from nucleus; 3/385; 7.2430E-04 mRNA transport; 3/385; 7.2430E-04 |  |  | protein-containing complex; 6/25; 5.7831E-03 |  |
| **Feature 86** |  | membrane-bounded organelle; 4/141; 1.7445E-03 organelle; 6/141; 2.4448E-03 intracellular membrane-bounded organelle; 4/141; 9.5441E-03 |  |  |  |  |
| **Feature 9** |  | positive regulation of GTPase activity; 4/217; 6.0959E-04 activation of GTPase activity; 4/217; 6.0959E-04 regulation of GTPase activity; 4/217; 1.4126E-03 positive regulation of hydrolase activity; 4/217; 1.6336E-03 |  |  | nuclear protein-containing complex; 4/19; 4.0684E-04 protein-containing complex; 5/19; 7.6791E-03 |  |
| **Feature 169** |  | regulation of actin filament organization; 3/302; 1.4036E-03 regulation of actin filament-based process; 3/302; 1.9656E-03 regulation of actin cytoskeleton organization; 3/302; 1.9656E-03 regulation of supramolecular fiber organization; 3/302; 4.4144E-03 |  |  |  |  |
| **Feature 114** |  |  |  |  |  |  |
| **Feature 90** |  | nucleoplasm; 7/288; 2.1284E-03 zinc ion binding; 3/288; 3.0278E-03 anatomical structure morphogenesis; 5/288; 3.2743E-03 protein kinase activity; 7/288; 5.8489E-03 |  |  |  |  |
| **Feature 205** |  | GTPase activator activity; 4/128; 8.5660E-04 GTPase regulator activity; 4/128; 8.5660E-04 nucleoside-triphosphatase regulator activity; 4/128; 8.5660E-04 enzyme activator activity; 4/128; 2.4076E-03 |  |  |  |  |
| **Feature 124** |  | cytosol; 8/101; 3.2350E-04 cysteine-type peptidase activity; 3/101; 5.1116E-04 cellular component organization or biogenesis; 11/101; 6.5317E-04 cellular component organization; 10/101; 2.1775E-03 |  |  | plasma membrane bounded cell projection; 3/22; 1.2679E-03 cell projection; 3/22; 1.5099E-03 |  |
| **Feature 226** |  | dendrite self-avoidance; 3/341; 1.0615E-04 neuron recognition; 3/341; 2.5943E-04 cell recognition; 3/341; 8.6792E-04 phospholipid binding; 5/341; 1.8500E-03 |  |  |  |  |
| **Feature 246** |  | cilium assembly; 3/122; 1.6219E-03 plasma membrane bounded cell projection assembly; 3/122; 2.0984E-03 cell projection assembly; 3/122; 2.0984E-03 cilium organization; 3/122; 2.6537E-03 |  |  |  |  |
| **Feature 234** |  | biological regulation; 3/81; 7.6066E-03 |  |  |  |  |
| **Feature 139** |  | establishment of localization in cell; 8/133; 7.7540E-05 cellular localization; 9/133; 8.6325E-05 intracellular transport; 7/133; 2.1824E-04 intracellular protein transport; 4/133; 2.9749E-03 |  |  |  |  |
| **Feature 217** |  | catalytic activity; 3/209; 7.2600E-03 microtubule binding; 3/209; 9.4477E-03 |  |  | cellular component assembly; 3/20; 1.2196E-03 |  |
| **Feature 140** |  | mRNA binding; 4/191; 1.9551E-03 molecular\_function; 29/191; 3.5558E-03 |  |  |  |  |
| **Feature 122** |  | protein kinase activity; 8/177; 5.2846E-05 phosphotransferase activity, alcohol group as acceptor; 8/177; 1.0849E-04 protein serine/threonine kinase activity; 6/177; 1.3528E-04 kinase activity; 8/177; 2.1989E-04 |  |  | protein kinase binding; 3/48; 2.6848E-04 kinase binding; 3/48; 2.9636E-04 lipid metabolic process; 3/48; 3.6199E-03 enzyme binding; 3/48; 4.5216E-03 |  |
| **Feature 213** |  | cell adhesion; 10/136; 1.0043E-09 biological adhesion; 10/136; 1.0043E-09 cell projection membrane; 4/136; 5.3629E-05 WW domain binding; 3/136; 5.7403E-05 |  |  | organic substance metabolic process; 5/19; 2.9527E-03 metabolic process; 5/19; 4.0549E-03 macromolecule metabolic process; 4/19; 7.7925E-03 |  |
| **Feature 29** |  | active transmembrane transporter activity; 12/856; 2.1946E-04 positive regulation of cell adhesion; 4/856; 4.3131E-04 active ion transmembrane transporter activity; 9/856; 7.2663E-04 regulation of cell adhesion; 5/856; 7.6965E-04 |  |  | protein kinase binding; 4/20; 1.8893E-07 kinase binding; 4/20; 2.1668E-07 enzyme binding; 4/20; 9.7986E-06 protein binding; 5/20; 3.0787E-03 |  |
| **Feature 240** |  | integrator complex; 5/456; 1.1463E-05 nuclear transport; 6/456; 4.7979E-04 nucleocytoplasmic transport; 6/456; 4.7979E-04 nuclear protein-containing complex; 20/456; 6.0634E-04 |  |  |  |  |
| **Feature 253** |  | nucleus; 23/232; 2.6087E-03 DNA-binding transcription repressor activity; 3/232; 7.4976E-03 DNA-binding transcription repressor activity, RNA polymerase II-specific; 3/232; 7.4976E-03 |  |  |  |  |
| **Feature 37** |  | protein kinase activity; 11/303; 1.5498E-05 phosphotransferase activity, alcohol group as acceptor; 11/303; 4.0501E-05 kinase activity; 11/303; 1.0321E-04 transferase activity, transferring phosphorus-containing groups; 11/303; 2.7379E-04 |  |  | membrane protein complex; 3/20; 2.1650E-03 |  |
| **Feature 100** |  | cation transport; 4/98; 2.8212E-03 inorganic cation transmembrane transport; 3/98; 7.7561E-03 ion transport; 4/98; 8.2698E-03 inorganic ion transmembrane transport; 3/98; 9.7167E-03 |  |  |  |  |
| **Feature 161** |  | inorganic molecular entity transmembrane transporter activity; 8/174; 2.8782E-04 inorganic cation transmembrane transporter activity; 7/174; 4.0179E-04 cation transmembrane transporter activity; 7/174; 5.9980E-04 symporter activity; 3/174; 7.1018E-04 |  |  | organonitrogen compound metabolic process; 8/47; 7.6631E-05 small molecule metabolic process; 4/47; 1.7523E-04 primary metabolic process; 9/47; 5.3735E-04 cellular metabolic process; 9/47; 8.2030E-04 |  |
| **Feature 57** |  | transferase complex; 14/338; 2.7601E-05 cellular anatomical entity; 88/338; 4.3550E-05 intracellular protein-containing complex; 12/338; 1.1532E-04 catalytic complex; 16/338; 1.9391E-04 |  |  | enzyme binding; 3/20; 3.4290E-04 |  |
| **Feature 252** |  | nuclear protein-containing complex; 21/149; 7.3859E-13 RNA polymerase II transcription regulator complex; 7/149; 4.0063E-09 catalytic activity, acting on a nucleic acid; 9/149; 1.8270E-07 transcription regulator complex; 7/149; 4.4114E-07 |  |  | histone methyltransferase complex; 3/23; 1.5954E-06 methyltransferase complex; 3/23; 3.2865E-06 nuclear protein-containing complex; 5/23; 6.2914E-05 catalytic complex; 4/23; 5.4198E-04 |  |
| **Feature 138** |  | regulation of transport; 3/68; 1.7499E-03 regulation of localization; 3/68; 7.5610E-03 extracellular space; 4/68; 9.6828E-03 |  |  |  |  |
| **Feature 222** |  | cytoplasmic ribonucleoprotein granule; 5/391; 2.8365E-04 ribonucleoprotein granule; 5/391; 6.1840E-04 tubulin binding; 5/391; 1.7341E-03 actin filament organization; 4/391; 1.8370E-03 |  |  |  |  |
| **Feature 200** |  | supramolecular fiber organization; 5/253; 2.8996E-04 actin filament organization; 4/253; 3.5951E-04 cellular macromolecule localization; 7/253; 5.9302E-04 macromolecule localization; 8/253; 2.4116E-03 |  |  | cell-cell junction; 3/20; 2.2678E-05 anchoring junction; 3/20; 5.9739E-05 cell junction; 3/20; 6.6496E-04 |  |
| **Feature 131** |  | positive regulation of cell adhesion; 4/807; 3.4308E-04 connexin complex; 5/807; 5.8601E-04 transporter activity; 26/807; 7.7860E-04 regulation of endocytosis; 3/807; 1.3694E-03 |  |  | binding; 7/20; 4.8846E-03 |  |
| **Feature 66** |  | glycosyltransferase activity; 4/166; 7.4867E-03 secretory vesicle; 3/166; 8.5665E-03 |  |  |  |  |
| **Feature 94** |  | intrinsic component of membrane; 46/215; 7.4932E-18 integral component of membrane; 45/215; 2.1096E-17 cellular anatomical entity; 112/215; 2.7396E-06 binding; 7/215; 2.8380E-05 |  |  | response to external stimulus; 3/24; 1.1226E-04 response to stimulus; 3/24; 4.0581E-03 |  |
| **Feature 171** |  | integral component of membrane; 19/124; 6.6031E-06 intrinsic component of membrane; 19/124; 8.5740E-06 exopeptidase activity; 3/124; 4.3555E-05 organelle membrane; 8/124; 2.0746E-04 |  |  | glycosyltransferase activity; 3/28; 2.9273E-04 transferase activity; 4/28; 4.9485E-03 |  |
| **Feature 40** |  | microtubule-based process; 6/110; 1.7860E-05 motor activity; 3/110; 1.0211E-04 microtubule cytoskeleton organization; 4/110; 2.1740E-04 centrosome; 4/110; 3.1332E-04 |  |  | centrosome; 3/35; 1.5197E-04 microtubule-based process; 3/35; 7.0776E-04 microtubule organizing center; 3/35; 9.4844E-04 organelle organization; 4/35; 2.2520E-03 |  |
| **Feature 158** |  | translation factor activity, RNA binding; 3/143; 6.6679E-05 RNA binding; 8/143; 1.6223E-04 translation regulator activity, nucleic acid binding; 3/143; 2.2232E-04 establishment of organelle localization; 3/143; 4.0009E-04 |  |  | binding; 7/20; 4.8846E-03 |  |
